# Reliably quantifying the evolving worldwide dynamic state of the COVID-19 outbreak from death records, clinical parametrization, and demographic data

**DOI:** 10.1038/s41598-021-99273-1

**Published:** 2021-10-07

**Authors:** Jose M. G. Vilar, Leonor Saiz

**Affiliations:** 1 Biofisika Institute (CSIC, UPV/EHU), University of the Basque Country (UPV/EHU), P.O. Box 644, 48080 Bilbao, Spain; 2 Basque Foundation for Science, IKERBASQUE, 48011 Bilbao, Spain; 3 Department of Biomedical Engineering, University of California, 451 E. Health Sciences Drive, Davis, CA 95616 USA

**Keywords:** Infectious diseases, Applied mathematics, Scientific data, Ecological epidemiology, Population dynamics, Computational models

## Abstract

The dynamic characterization of the COVID-19 outbreak is critical to implement effective actions for its control and eradication but the information available at a global scale is not sufficiently reliable to be used directly. Here, we develop a quantitative approach to reliably quantify its temporal evolution and controllability through the integration of multiple data sources, including death records, clinical parametrization of the disease, and demographic data, and we explicitly apply it to countries worldwide, covering 97.4% of the human population, and to states within the United States (US). The validation of the approach shows that it can accurately reproduce the available prevalence data and that it can precisely infer the timing of nonpharmaceutical interventions. The results of the analysis identified general patterns of recession, stabilization, and resurgence. The diversity of dynamic behaviors of the outbreak across countries is paralleled by those of states and territories in the US, converging to remarkably similar global states in both cases. Our results offer precise insights into the dynamics of the outbreak and an efficient avenue for the estimation of the prevalence rates over time.

## Introduction

The global spread of the COVID-19 outbreak has had a major global impact. As of January 21, 2021, there have been 2.50 million reported deaths and 122.1 million confirmed cases^1^. Massive travel restrictions, imposed quarantines, lockdowns, and other nonpharmaceutical interventions (NPIs) around the world have been able to slow down the progression of the outbreak, but their gradual lifting has already led to its deadly resurgence in multiple areas^2^. Currently, it is still unclear how best to proceed to balance the economic, societal, ethical, and health trade-offs present. The most widespread quantification, based on the basic reproduction number, is insufficient to address the different trade-offs^3^. It indicates whether the outbreak is growing up or dying out, but it misses crucial information, including the timescales of the processes, the latent potential for a resurgence of the outbreak, and the possibility of actively controlling and tracing the infectious population.

Obtaining a detailed dynamic characterization of the outbreak, however, has proved to be particularly challenging and has been achieved only over small controlled populations. This characterization involved testing for the causative virus SARS-CoV-2, identifying contacts and the infection time, and following up the clinical evolution^4–7^. These analyses have led to a precise mathematical characterization of the clinical evolution of the disease, including the distributions of times from infection to symptom onset, from symptom onset to death, and from primary to secondary infections.

The available epidemiological information, however, has not been sufficiently reliable to be used directly and the detailed characterization is not feasible at a global scale. Methods based on compartmental models^8^ or Bayesian approaches^9^ have relied on location-specific information that is not readily available at a global scale. These methods have typically pivoted toward using either the number of reported COVID-19 cases over time or the reported death counts over time.

Focusing on the number of cases is challenging because they represent only an undetermined fraction of the actual infections and this fraction depends on the testing capacity, which was remarkably low in the early stages of the pandemic, and on how the testing capacity evolves over time. In addition, there are a large fraction of asymptomatic infections, some of which are uncovered through contact tracing approaches while others remain undetected^8^. Moreover, it is not straightforward to determine when a positive testing person was infected, which depends on the location-specific testing procedures as well. Therefore, the connection between reported cases and the actual number of infections depends on the testing policies of each location, how they are implemented, and how they change over time.

An alternative approach is to use daily death counts. In this case, there is detailed statistical information on the disease progression over time through its different stages from infection to potential death, as well as on the age-stratified death ratios. The main drawbacks of this approach are on the mathematical side because it requires the solution of an inverse problem (finding the infectious population that would lead to the observed death curves) and on the fact that death counts are typically much lower than the number of infections, which make them prone to the inherent random fluctuations of the death process. These challenges are usually overcome by restricting the analyses to locations with large numbers of deaths and by imposing constraints on the reproduction number based on the information available about NPIs^9^.

Here, we provide a general quantitative approach for reliably quantifying the temporal evolution of the COVID-19 outbreak infectious and infected population utilizing multiple data sources, including daily death records, clinical parametrization of the disease, and location-specific demographic data. Explicitly, we use the customary infection-age structured mathematical description^4,10–12^, which relies on the detailed statistical characterization of the disease progression over time, to develop an approach to obtain explicit estimates of the number of infectious and infected individuals in terms of the epidemiological death curves. We find that there is a general time delay between the infectious population and the daily deaths and a different time delay between the infected population and the cumulative number of deaths, which depend on the clinical parameters of the disease. The major location-specific contribution, besides the death counts, is the age structure of the population, which determines the infection fatality rate (IFR) and therefore the proportionality factors between infectious population and daily deaths and between infected population and cumulative deaths. We validated the approach with prevalence data of the infectious (PCR-RT testing) and infected (antibody testing) populations at a global scale and for states within the US, as well as with the timings of the peak infectiousness against the dates of the major country-wide lockdowns in Europe. To further quantify the temporal evolution and controllability of the COVID-19 outbreak we also obtained the temporal evolution of the growth rate of the infectious population. We consider explicitly countries in the world and states and territories within the United States (US) with at least 30 COVID-19 reported deaths as of January 21, 2021, which covers 97.4% of the world and 99.9% of the US population.

## Results

### Optimal dynamical constraints

The approach considers the dynamics of the infectious population, ${n}_{I}(t)$, at time $t$ described through the expression

1 $$\begin{array}{c}\frac{d{n}_{I}\left(t\right)}{dt}={k}_{G}\left(t\right){n}_{I}\left(t\right),\end{array}$$

which establishes the definition of the (per capita) growth rate ${k}_{G}(t)$. As this expression results from the definition of the per capita growth rate, ${k}_{G}\left(t\right)\equiv \frac{1}{{n}_{I}\left(t\right)}\frac{d{n}_{I}\left(t\right)}{dt}$ , it is completely general and independent of the underlying dynamics of the infection.

The underlying infection dynamics dictates the relationship of ${k}_{G}\left(t\right)$ and ${n}_{I}\left(t\right)$ with the different epidemiological quantities. Based on an infection-age structured mathematical description^10,11^ (See “Methods: Infection-age structured dynamics”), we have developed an approach to uncover the optimal dynamic constraints for these relationships in terms of delays and scaling factors (See “Methods: Dynamical constraints”).

We find that ${n}_{I}(t)$ is optimally related to the rate of increase of the expected cumulative deaths, ${n}_{D}$, at a later time $t+{\tau }_{D}$ according to

2 $$\begin{array}{c}{n}_{I}\left(t\right)=\frac{{\tau }_{G}}{IFR}\frac{d{n}_{D}\left(t+{\tau }_{D}\right)}{dt},\end{array}$$

where $IFR$ is the infection fatality rate and ${\tau }_{G}$ is the average generation time. Note that the first derivative of the expected cumulative number of deaths is obtained from the expected number of daily deaths as $\frac{d{n}_{D}\left(t\right)}{dt}={n}_{D}\left(t\right)-{n}_{D}(t-1)$ (See “Methods: Dynamical constraints implementation with discrete time”) and that expected deaths are obtained from raw death counts reported by the Johns Hopkins University Center for Systems Science and Engineering^1^ (See “Methods: Expected deaths”).

The preceding equation for ${n}_{I}\left(t\right)$ explicitly takes into account that, on average, an infectious individual within ${n}_{I}(t)$ has been infected at time $t-\frac{{\tau }_{G}^{2}+{\sigma }_{G}^{2} }{2{\tau }_{G}}$ and potentially dies with probability $IFR$ at a time ${\tau }_{I}+{\tau }_{OD}$ after infection. Here, ${\sigma }_{G}^{2}$ is the variance of the generation time, and ${\tau }_{I}$ and ${\tau }_{OD}$ are the incubation and symptom onset-to-death average times, respectively. The values of these characteristic times have been estimated in days as ${\tau }_{G}=6.5$, ${\sigma }_{G}=4.2$, ${\tau }_{I}=6.4$, and ${\tau }_{OD}=17.8$ from precise follow up of specific groups of patients^5,13,14^, which leads to ${\tau }_{D}={\tau }_{I}+{\tau }_{OD}-\frac{{\tau }_{G}^{2}+{\sigma }_{G}^{2} }{2{\tau }_{G}}=19.6$ (See “Methods: Dynamical constraints” and “Methods: Clinical parameters ”). Therefore, the value of ${\tau }_{D}$ can be interpreted as the average number of days from infection to death (${\tau }_{I}+{\tau }_{OD}$) minus the average number of days that an individual remains infectious after infection ($\frac{{\tau }_{G}^{2}+{\sigma }_{G}^{2} }{2{\tau }_{G}}$).

A general assumption of the approach is that the $IFR$ for each age group remains the same for all locations^5^ and that the overall $IFR$ for each location is obtained as the average over its specific population's age distribution (See “Methods: Infection fatality rate $(IFR)$”).

The growth rate is obtained directly from Eqs. (1) and (2) as

3 $$\begin{array}{c}{k}_{G}\left(t\right)=\frac{d}{dt}{\mathrm{ln}}\frac{d{n}_{D}\left(t+{\tau }_{D}\right)}{dt} ,\end{array}$$

which is related to the time-varying reproduction number, ${R}_{t}$, through the Euler–Lotka equation (See “Methods: Reproduction number”).

The expected cumulative number of infected individuals at a time $t$, ${n}_{T}\left(t\right)$, follows from

4 $$\begin{array}{c}{n}_{T}\left(t\right)=\frac{1}{IFR}{n}_{D}\left(t+{\tau }_{I}+{\tau }_{OD}\right) ,\end{array}$$

which is obtained also from the dynamical constraints (See “Methods: Dynamical constraints”).

To compare with prevalence studies, we used the dynamical constraints (See “Methods: Dynamical constraints”) to obtain the relationship of the expected number of seropositive individuals, ${n}_{SP}\left(t\right)$, with the infected population,

5 $$\begin{array}{c}{n}_{T}\left(t\right)={n}_{SP}\left(t+{\tau }_{SP}\right) ,\end{array}$$

where ${\tau }_{SP}$ is the average seroconversion time after infection. We also obtained the relationship of the expected number of positive reverse transcription polymerase chain reaction (RT-PCR) testing individuals, ${n}_{TP}(t)$, with the infectious population,

6 $$\begin{array}{c}{n}_{I}\left(t\right)=\frac{{\tau }_{G}}{\Delta {t}_{TP}}{n}_{TP}\left(t+{\tau }_{TP}-\frac{{\tau }_{G}^{2}+{\sigma }_{G}^{2} }{2{\tau }_{G}}\right),\end{array}$$

where ${\tau }_{TP}$ is the average time for testing positive after infection and $\Delta {t}_{TP}$ is the average number of days of positive testing. The values of these additional characteristic times have been estimated in days as ${\tau }_{SP}=13.4$, ${\tau }_{TP}=14.4$, and $\Delta {t}_{TP}=20$ from clinical studies^14–17^ (See “Methods: Clinical parameters”).

### Implementation

**Figure 1:**
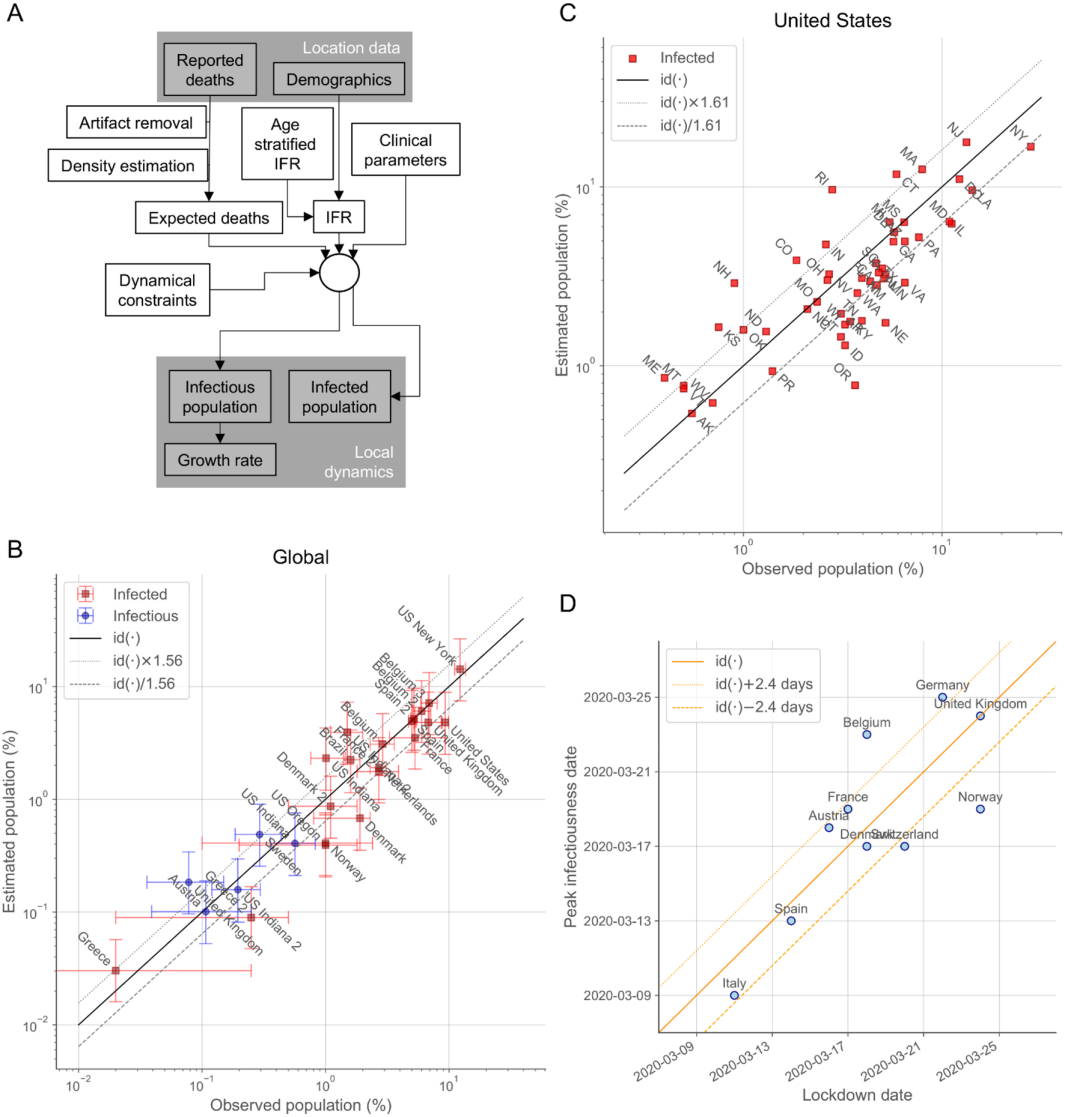
The approach consistently estimates prevalence and the timing of NPIs. The approach (**A**) has been validated with prevalence data of the infectious (PCR-RT testing) and infected (antibody testing) populations at a global scale (**B**) and for states within the US (**C**). The continuous black lines (**B**,**C**) represent the perfect prediction (identity function denoted by ${\mathrm{id}}(\cdot )$) with the parallel dotted/dashed lines indicating the fold accuracy. Global data were obtained from sources described in Supplementary Table S1. Several locations have estimates for multiple date ranges. State data correspond to two studies with specimen samples taken primarily on the first two weeks of July^20^ and of August, 2020^21^. The inferred timings of the peak infectiousness are plotted against the dates of the major country-wide lockdowns in Europe^9^ (**D**). The continuous orange line represents perfect concordance and the parallel dotted/dashed lines indicate the mean absolute error.

Equations (1–4) completely characterize the dynamics of the outbreak from the expected cumulative deaths, the age structure of the population, and the general clinical parameters of the infection. We used a workflow (Fig. 1A) that incorporates explicitly the death counts compiled by the Johns Hopkins University Center for Systems Science and Engineering^1^, the age structure reported by the United Nations for countries^18^ and the US Census for states and territories^19^, previously estimated aged-stratified $IFR$^5^, and other previously estimated clinical parameters^5,13–17^. The expected number of daily deaths was inferred using density estimation from the death curves after preprocessing to minimize reporting artifacts (See “Methods: Inference and extrapolation”). Equations (5) and (6) were used to set the appropriate scale and delay to compare with seroprevalence studies.

### Validation against prevalence data and NPIs

To validate the approach, we contrasted the estimated infectious and infected populations with the results from available antibody seroprevalence and RT-PCR testing studies for countries in the world and locations in the US with at least 30 reported deaths (Fig. 1B). The observed and estimated values agree with each other within the 95% credibility intervals (CrI), with a 1.56-fold accuracy over almost a 1,000-fold variation and a correlation coefficient ($\rho$) on a logarithmic scale of $\rho =$ 0.94.

We combined into a separate analysis (Fig. 1C) the data of two additional comprehensive antibody seroprevalence studies within the US, which included 49^20^ and 38^21^ states with non-zero prevalence values. For the states present in the two studies, we considered their average values. The estimated values agree with the observed prevalence with 1.61-fold accuracy and $\rho =$ 0.80. The agreement for the combined data is better than for the data of each of the studies independently (1.65-fold accuracy and $\rho =$ 0.73 for one study^20^ and 1.74-fold accuracy and $\rho =$ 0.77 for the other study^21^) and better than the agreement of both studies with each other ($\rho =$ 0.55 for the 38 states common to both studies), indicating that the estimations of the approach fall within the observed variability of the prevalence studies. Indeed, the approach is effectively unbiased collectively, with the (geometric) average of the estimations being just a factor 1.12 (globally) and 1.13 (US) larger than that of the observations.

Overall, the validation of the approach shows that it can reproduce the available prevalence studies over a factor 615.0 between minimum and maximum values for countries and states with 72.0% (globally) and 77.6% (US) of the estimates within a factor 2 of the observed values, and with 100.0% (globally) and 93.9% (US) within a factor 3. There are no countries and only three states with values outside the factor 3 boundary. In the cases of Rhode Island and New Hampshire, the estimated infectious population is larger than the ones reported by one study^21^, which is consistent with the observed age-specific seroprevalence biased to older populations. In the case of Oregon, the estimated infectious population is smaller than the ones reported^20,21^, which is consistent with the observed age-specific seroprevalence biased to younger populations^21^. In the case of Oregon, in addition, only 338 of the 1123 excess deaths during the outbreak before the collection of the specimens for analysis were attributed to COVID-19^22^.

We also validated the ability of the approach to capture the effects of NPIs (Fig. 1D). The inferred timings of the peak infectiousness (maximum infectious population) are concordant with the dates of the major early country-wide lockdowns in Europe^9^, with an overall average deviation of 0.0 days between lockdown and peak dates and a mean absolute deviation of 2.4 days. This concordance also indicates that there is only a small variability in the average timing between infection and death among those countries.

### Outbreak progression motifs

We analyzed explicitly the time evolution of the growth rate, the infectious population, the cumulative number of infections, and the relationship between growth rate and infectious population for all countries and states with at least 30 deaths (Supplementary Fig. S1 and Table 1).

**Table 1:** Key estimates of the outbreak.

	World	United States
Highest local maximum of the infectious population	7.35 million on July 17, 2020	1.42 million on March 28, 2020
Current infectious population	14.46 million on January 21, 2021	2.42 million on January 21, 2021
Infected population	375.0 million as of January 21, 2021	52.0 million as of January 21, 2021
Underreporting factor	3.1 (only 122.1 million cases reported)	2.1 (only 24.6 million cases reported)
New infections per week	15.70 million	2.62 million
Growth rate: 10% quantile	− 0.015/days (45.7-day half-life)	− 0.020/days (34.5-day half-life)
Growth rate: median	0.000/days	0.000/days
Growth rate: 90% quantile	0.026/days (27.1-day doubling time)	0.011/days (64.5-day doubling time)
Population considered (locations with at least 30 deaths)	97.4%	99.9%
Number of locations considered	154	53
Number of locations with more than 10% of the population infected	44	43
Number of locations with more than 20% of the population infected	8	11
Number of locations with less than 7 actively infectious individuals per 100,000 population	22	0
Number of locations that reached 1,000 actively infectious individuals per 100,000 population	27	14
Correlation coefficient between infectious/infected population estimates and observations	0.94	0.80
Infectious/infected population estimates within a factor 2 of the observations	72.0%	77.6%
Infectious/infected population estimates within a factor 3 of the observations	100.0%	93.9%

**Figure 2:**
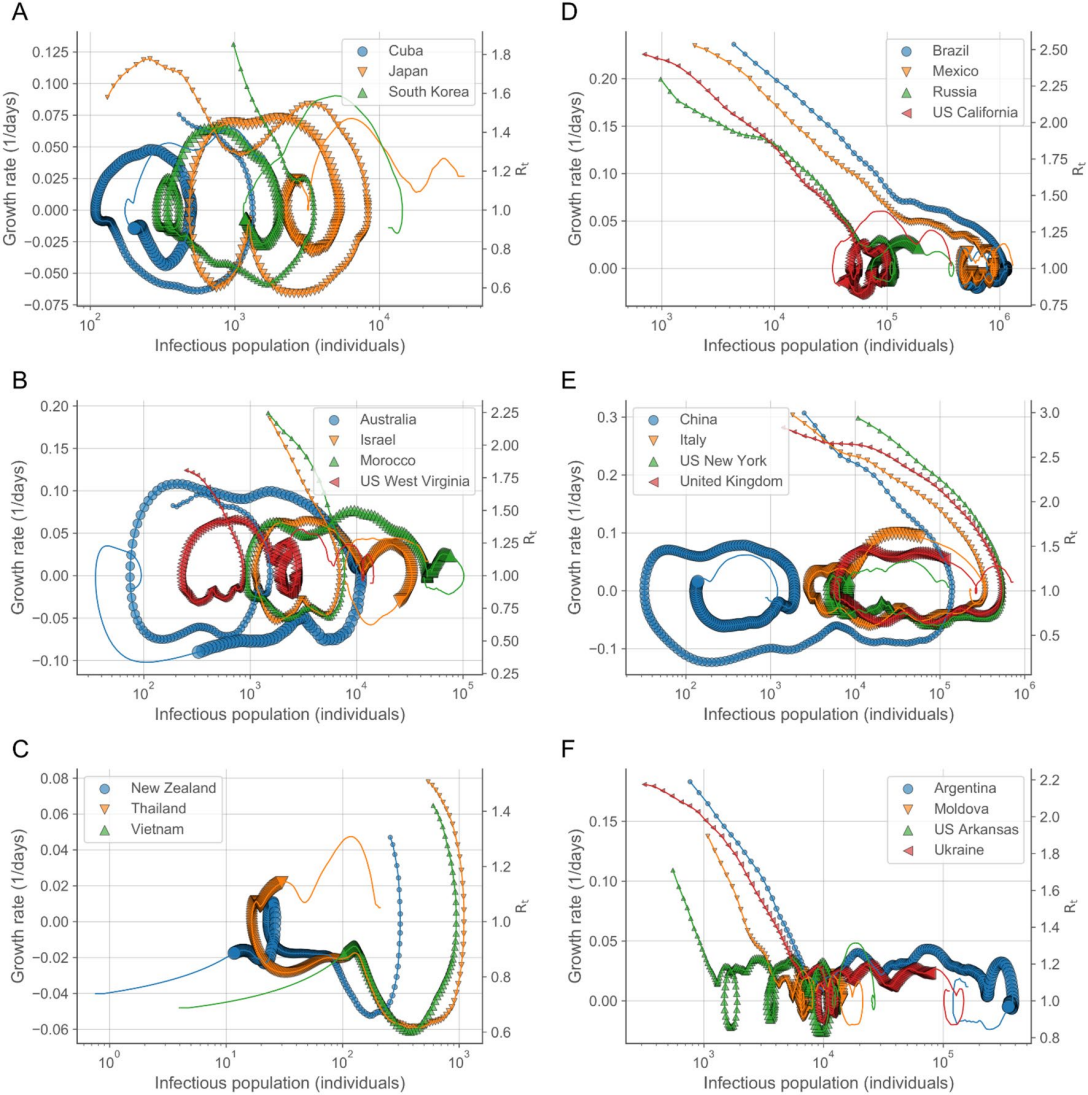
Trajectories in the growth rate-infectious population space followed prototypical types of behavior in the first-middle stages of the outbreak. Each day is indicated by a symbol increasing in size with time. The largest symbol corresponds to October 1, 2020. Complete trajectories, up to December 30, 2020, are indicated by the colored lines. The right axes indicate the growth rate in the scale of the reproduction number as ${R}_{t}=1+{k}_{G}\left(t\right){\tau }_{G}$ (See “Methods: Reproduction number”). In all cases in the figure, the trajectory starts with positive growth rate (${R}_{t}>1$) at the smallest symbol and moves rightwards increasing the infectious population until the growth rate becomes negative (${R}_{t}<1$), at which point the infectious population starts to decrease and the trajectory moves leftwards. After the growth rate changes to negative values, the trajectories exhibit multiple types of differentiated behavior that can be classified in several groups depending on the extent of the initial and subsequent growth. If the growth rate reverts to positive values, the trajectory would move rightwards again. Common types of behavior include initial containment (**A**–**C**) with subsequent minor increases (**A**), amplified resurgence (**B**), and a sustained regression (**C**) of the outbreak; and initial amplification (**D**–**F**) with slow convergence to zero growth (**D**), convergence to negative growth (**E**), and fast convergence to initial sustained residual positive growth (**F**). The prefix "US " has been added to the name of the locations in the US. Confidence intervals for the growth rates and infectious population are provided in Supplementary Fig. S1.

The characterization of the dynamics in terms of the growth rate and infectious population (Supplementary Fig. S1) shows that there are prototypical types of behavior, or motifs (highlighted with representative examples in Fig. 2). Locations either contained (Fig. 2A–C) or amplified (Fig. 2E,F) the outbreak in its initial stages. In the case of initial containment, the growth rate switched rapidly to negative values. Subsequently, the behavior branched into contained sporadic resurgences below the initial maximum infectiousness (Fig. 2A), uncontrolled resurgence of the infectious population over the initial maximum infectiousness (Fig. 2B), and sustained decrease of the outbreak (Fig. 2C). The specific behavior depended on the success of the measures implemented, e.g. targeted control and moderate lockdowns, and their subsequent relaxation^23^. In the case of initial amplification, the dynamics proceeded in diverse ways, including an increasing infectious population converging to zero growth (Fig. 2D), a fast-evolving infectious population switching from positive to negative growth (Fig. 2E), and fast convergence to subsequent sustained residual growth (Fig. 2F). In general, the locations that reached a substantial negative growth rate are those that implemented long-term strict lockdowns, whereas zero or small positive growth rates correspond to intermediate measures with partial restrictions^23^. In many cases, lifting restrictions has led to fast switching from negative to positive sustained growth, as illustrated by United Kingdom, and Italy (Fig. 2E), indicating the inability of these locations to contain the outbreak even at low values of the infectious population.

### Global dynamics

**Figure 3:**
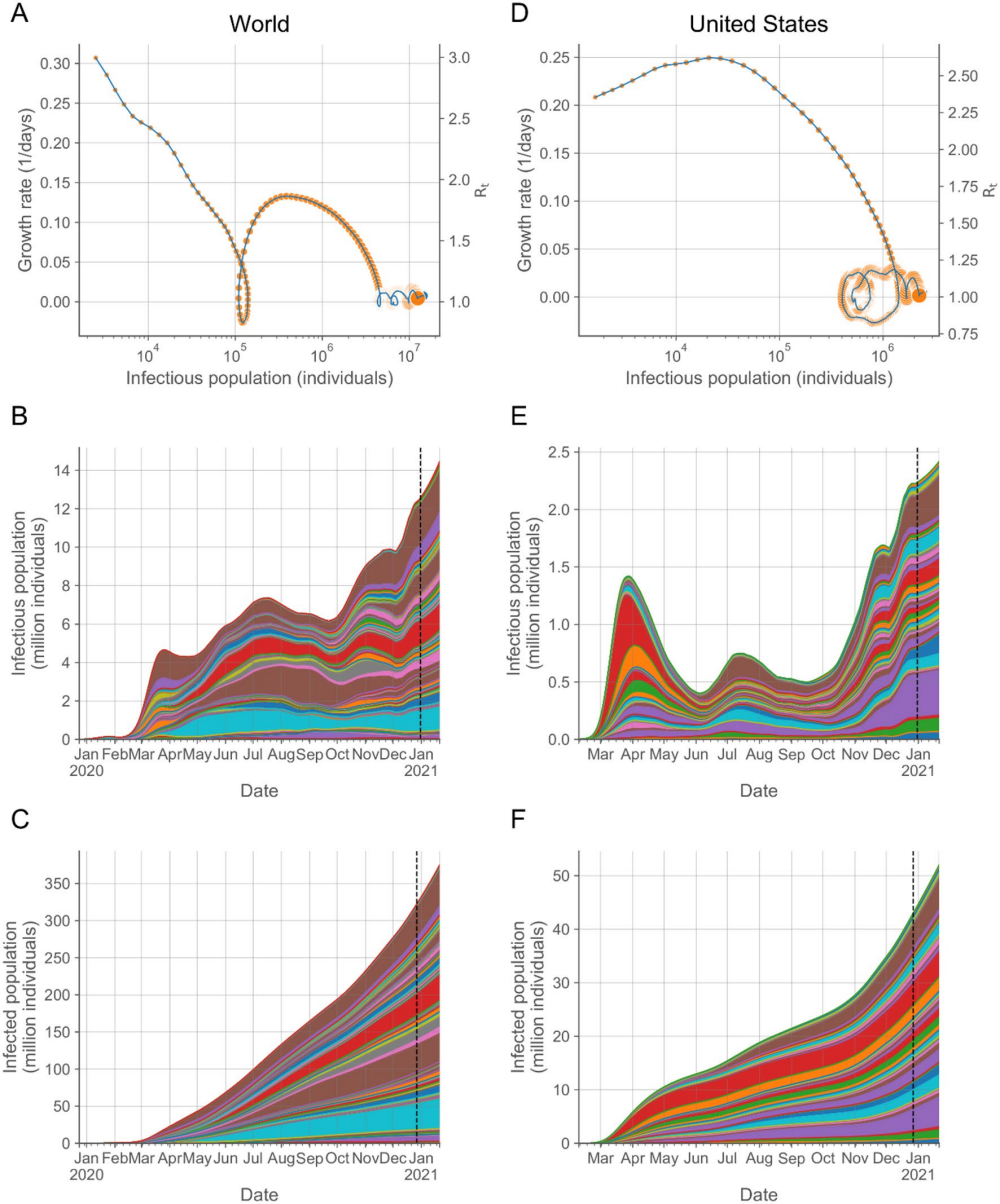
Characterization of the temporal evolution of the COVID-19 outbreak. Trajectory in the growth rate-infectious population space (**A**,**D**) and infectious (**B**,**E**) and infected (**C**,**F**) populations over time of the aggregate values for countries in the world (**A**–**C**) and locations in the US (**D**–**F**). In the trajectories (**A**,**D**), each day is indicated by a symbol increasing in size with time, ending with the largest symbol on December 30, 2020. The blue line at the end indicates the extrapolation to current time (January 21, 2021). The right axes indicate the growth rate in the scale of the reproduction number as ${R}_{t}=1+{k}_{G}\left(t\right){\tau }_{G}$ (See “Methods: Reproduction number”). Each colored region in the area plots represents the contribution of a country (**B**,**C**) or a state (**E**,**F**) to the overall infectious and infected populations. Countries and states are arranged in alphabetical order from bottom to top. The vertical dashed lines (**B**,**C**,**E**,**F**) indicate the transition from inference to extrapolation. Individual data for all the countries and states are provided in Supplementary Fig. S1.

At a global scale, the outbreak is characterized by two early local maxima of the overall worldwide infectious population on January 25, 2020 and March 26, 2020, coincidental with the regression of the outbreak in China, initially, and in Europe, afterward, reaching the highest local maximum of 7.35 million active infections (Fig. 3A,B) on July 17, 2020 with a subsequent increasing trend since September 22, 2020. In the US, there has been a local maximum of the infectious population on March 28, 2020 with 1.42 million active infections, a smaller local maximum on July 14, 2020, and a subsequent increasing trend since September 15, 2020 (Fig. 3D,E). The estimated infected population is 375.0 million, growing at a rate of 15.70 million new infections per week, for the World (Fig. 3C) and 52.0 million, growing at a rate of 2.62 million new infections per week, for the US (Fig. 3F), which is a factor 3.1 for countries in the World and 2.1 for locations in the US higher than the corresponding reported cases^1^.

### State of the outbreak across locations and its controllability

**Figure 4:**
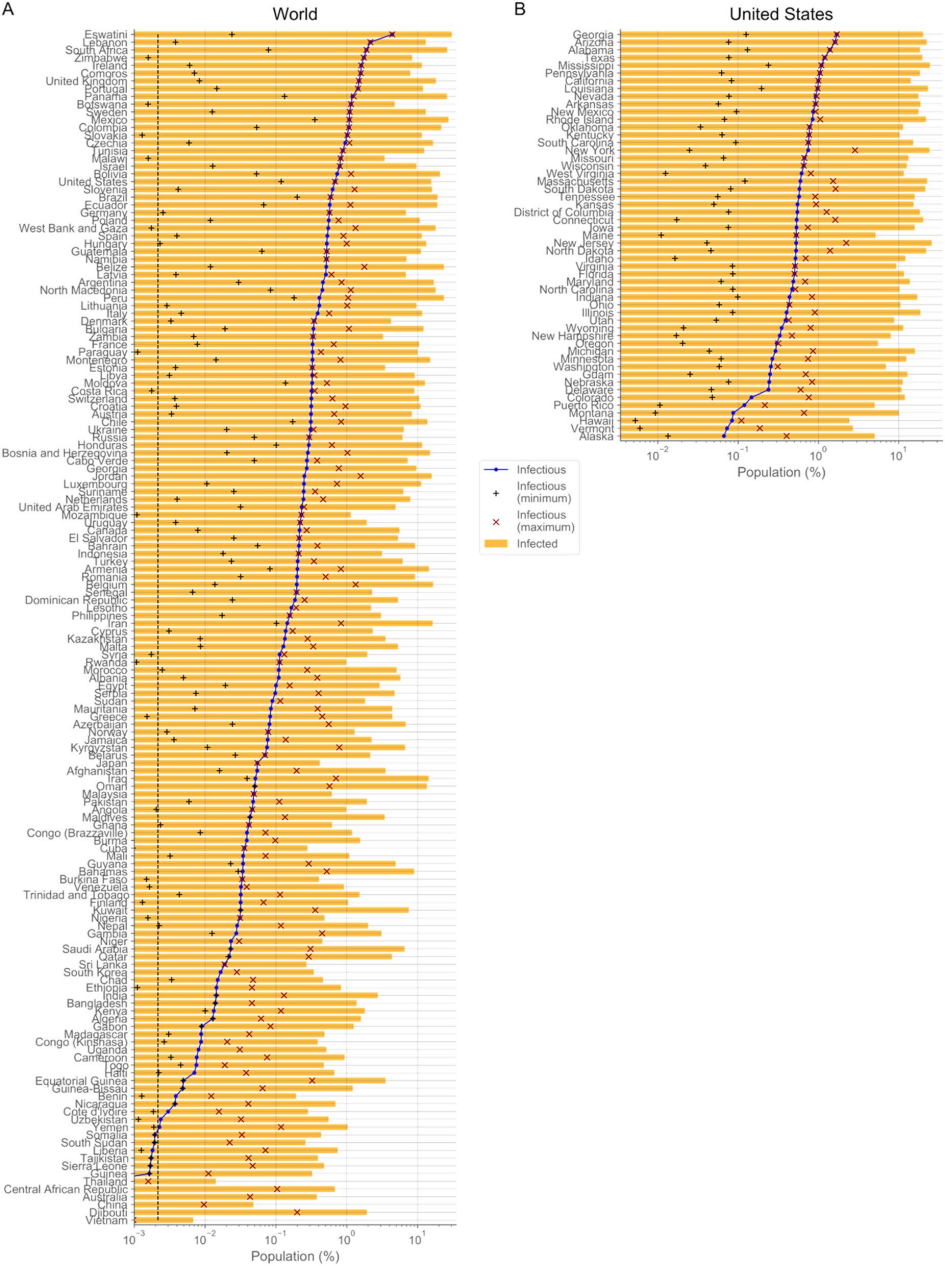
Per capita state of the outbreak. The percentage of infectious and infected populations on the last day estimated (January 21, 2021) and of the minimum and maximum infectious population reached are shown for countries in the world (**A**) and locations in the US (**B**). The minimum was computed for values after May 1, 2020. Countries and US locations have been sorted according to their per capita infectious population. The vertical dashed line (**A**) indicates the time-averaged infectious population of South Korea from March 1 to October 1, 2020.

At a local scale, the per capita infectious population of countries and states has only exceptionally crossed the 1% value (27 out of 154 countries and 14 out of 53 states) (Fig. 4). The infected populations have surpassed the 10% of the total population only for 44 countries and 43 states (Fig. 4), with 8 countries and 11 states reaching the 20% value. These results confirm that relying on herd immunity is not a realistic option. Controlling the outbreak by actively tracing and quarantining newly and potentially infected individuals has been successfully implemented, until early October, 2020, in South Korea, with the use of large resources and with occasional outbreaks that required short-term extended human-interaction restrictions, and almost successfully in Japan, with voluntary business closures and other restrictions^23^. These countries always remained below 7 actively infectious cases per 100,000 individuals (0.007% of their population) until early October, 2020, with average values of 2.2 (South Korea) and 2.6 (Japan) from March 1 to October 1, 2020. Only 0 of the states and 22 of the countries analyzed have had a per capita number of infectious individuals below the average value of South Korea since May 1, 2020, which indicates that 54.1% of the World (72.6% excluding China) and 97.9% of the US human population reside in countries or states that have not allowed targeted controllability of the outbreak with the resources used by South Korea. This inability to control the outbreak as soon as restrictions are lifted, even at low values of the infectious population but above the South Korea average value, is illustrated by United Kingdom and Italy (Fig. 2E).

### From local to global dynamics

**Figure 5:**
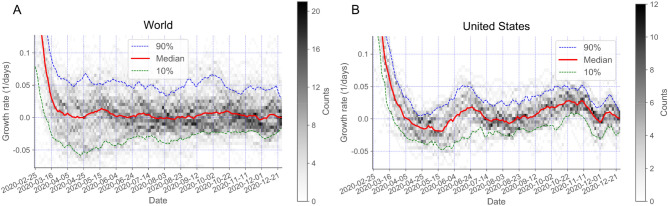
The temporal evolution of the distribution of growth rates shows a double stabilization of the outbreak. The temporal evolution of the median (red line), first decile (green line), and tenth decile (blue line) of the growth rates are plotted over the gray-coded histogram for countries in the world (**A**) and states and territories of the US (**B**). The median converges towards zero values and the deciles move closer to each other in a fluctuating manner for countries in the World and locations in the US. The double stabilization means that growth rates have mainly moved towards zero values both at local scales and at a global scale. At local scales, growth rates for most countries and states, whether positive or negative, decreased in absolute value, leading to a slowdown of the dynamics. At World and US global scales, positive and negative values for each of the locations have converged towards statistically compensating each other, decreasing further the overall net growth of the infectious population.

The collective properties of the individual local dynamics, as quantified by the distribution of growth rates across countries and states over time (Fig. 5), shows a progressive double stabilization of the outbreak. The double stabilization means that growth rates have mainly moved from large initial values towards zero values both at local scales and at a global scale. At local scales, growth rates for most countries, whether positive or negative, decreased in absolute value, leading to a slowdown of the dynamics. At a global scale, positive and negative values have converged towards statistically compensating each other, decreasing further the overall net growth of the infectious population. Specifically, the latest estimates of the growth rates on December 30, 2020 for countries in the world have a median value of 0.000/days, with 80% of the countries within the narrow range of values from − 0.015/days to 0.026/days, which implies a very slow local dynamics with half-lives and doubling times of 45.7 and 27.1 days, respectively. Similarly, the median value of the growth rates for locations in the US is 0.000/days, with 80% of locations ranging from − 0.020/days (34.5 days half-life) to 0.011/days (64.5 days doubling time).

## Discussion

The dynamical constraints we have obtained through a detailed infection-age mathematical description of the outbreak allowed us to find the optimal time delays and scaling factors to connect the evolution of the reported death counts over time with those of the infectious and infected populations. Overall, integrating these constraints through a workflow with essential preprocessing steps showed that the approach can consistently infer the precise timing of NPIs and estimate prevalence data across countries in the world and locations in the US. A prominent feature of the approach is its ability to provide reliable results even for low death counts, which overcomes the major limitations of choosing between unreliable infection case data (highly dependent on testing rates) or noisy death counts as input to the inference problem^9^.

The approach assumes a general age-stratified $IFR$. In general, these quantities are expected to depend potentially on specific features of the population and the medical care facilities available. The available studies show a minimal variability among different countries and other locations that reported on prevalence^24^. It also assumes an age-uniform exposure (attack rate), which is consistent with data for other respiratory diseases^5^ and holds to a large extent when there is information available for COVID-19^20,25,26^. We have also assumed a constant generation interval typical of non-confinement locations, which has been observed to shorten in some cases by NPIs^27^. Prevalence studies can also depend on the diminishing antibody levels after infection^15,28^, collecting and processing specimens for analysis^29^, and potential biases towards specific population groups^20^. In addition, there might be a degree of under-reporting of COVID-19 deaths, as suggested by excess mortality not attributable to other causes than COVID-19^22^. Our results show that all of these potential deviations on the assumptions, on the data, and on prevalence studies collectively have only a restricted impact on the approach, with 72.0% (globally) and 77.6% (US) of the estimates within a factor 2 of the observed values and 100.0% (globally) and 93.9% (US), within a factor 3. This accuracy of the estimations is highly remarkable in the context of the observed prevalence spread over a factor 615.0 between minimum and maximum values, from 0.02 to 12.30%.

The analysis in terms of the growth rate–infectious population trajectories has revealed universal types of behavior of the outbreak for countries around the world and locations within the US. This information can be used to anticipate the response to enacting, modifying, or lifting NPIs. The most marked example is the response to strict lockdowns across countries in Europe (e.g. United Kingdom, Italy, Belgium, Spain, France, Germany, Austria, Netherlands, and Switzerland) and Northeast states in the US (e.g. New York, New Jersey, Massachusetts, Pennsylvania, and District of Columbia). They followed the same type of behavior (fast decrease of the growth rate from high to sustained negative values) until major restrictions were lifted in the European countries^23^, turning the growth rate of their infectious populations into highly sustained positive values. Our results show that those European countries had small actively infectious populations but not as small as required for targeted controllability. They also show that most Northeast states in the US are in a similar resurgence path but at much earlier stages, with many of their NPIs still in place and with markedly smaller growth rates, which makes their reaching as deadly a resurgence as in Europe still avoidable.

At a global scale, the outbreak has reached a net growth rate fluctuating near zero values but with a high infectious population. A similar state has also been reached in the US. This type of fluctuating states, with long stagnant overall infectious population periods and median growth rate close to zero, is expected of bounded unsynchronized fluctuating populations^30^, such as those from uncoordinated locations aiming at just preventing an unrestricted expansion of the outbreak rather than at its eradication. This widespread feature is present for both countries in the world and locations within the US. Considering the NPIs implemented^23^, our results show that there have been locations with interventions to move the growth rate towards zero values and that there have been locations switching on and off severe measures to decrease temporarily the active infectious population. Despite not growing substantially since reaching its highest local maximum of 7.35 million active infections on July 17, 2020, the high value of the global infectious population attained is currently leading to 15.70 million new infections per week that replace the same ballpark number of individuals that stop being infectious. This high turnover makes the control of any potential resurgence extremely costly.

At a local scale, our results show a highly variable temporal evolution of the infectious populations, both over time for each location and across locations. Having an up-to-date estimate of the infectiousness of populations would allow policymakers to better implement travel planning among locations. The approach has proven to accurately track the effects of local NPIs. We also expect it to play a fundamental role in evaluating the progress of vaccination efforts, especially considering the challenges present, such as waning immunity levels and pathogen evolution^31^.

## Methods

### Infection-age structured dynamics

For the description of the dynamics, we follow the customary infection-age structured approach (for details see for instance Refs.^4,10–12^). Explicitly, we consider the infection-age structured dynamics of the number of individuals ${u}_{I}\left(t,\tau \right)$ at time $t$ who were infected at time $t-\tau$ given by

7 $$\begin{array}{c}\frac{\partial }{\partial t}{u}_{I}\left(t,\tau \right)+\frac{\partial }{\partial \tau }{u}_{I}\left(t,\tau \right)=0\end{array}$$

with boundary condition

8 $$\begin{array}{c}{u}_{I}\left(t,0\right)=j\left(t\right).\end{array}$$

Here, $\tau$ is the time elapsed after infection, referred to as infection age, and $j\left(t\right)={\int }_{0}^{\infty }{k}_{I}(t,\tau ){u}_{I}\left(t,\tau \right)d\tau$ is the incidence, with ${k}_{I}(t,\tau )$ being the rate of secondary transmissions per single primary case.

The solution is obtained through the method of characteristics^32^ as

9 $$\begin{array}{c}{u}_{I}\left(t,\tau \right)=j\left(t-\tau \right)\end{array}$$

for $t\ge \tau$ and ${u}_{I}\left(t,\tau \right)=0$ for $t<\tau$. The resulting renewal equation, $j\left(t\right)={\int }_{0}^{\infty }{k}_{I}\left(t,\tau \right)j\left(t-\tau \right)d\tau$, is used as the basis for the definitions of the reproduction number ${R}_{t}={\int }_{0}^{\infty }{k}_{I}\left(t,\tau \right)d\tau$ and the probability density of the generation time ${f}_{GT}\left(\tau \right)=\frac{{k}_{I}\left(t,\tau \right)}{{R}_{t}} .$

The infectious population is given by

10 $$\begin{array}{c}{n}_{I}\left(t\right)={\int }_{0}^{\infty }{P}_{I}\left(\tau \right){u}_{I}\left(t,\tau \right)d\tau ,\end{array}$$

which considers that an individual remains potentially infectious after a time $\tau$ from infection with probability

11 $$\begin{array}{c}{P}_{I}\left(\tau \right)={\int }_{\tau }^{\infty }{f}_{GT}\left(l\right)dl.\end{array}$$

Therefore, in terms of the incidence [substituting Eq. (9) in Eq. (10)], we have

12 $$\begin{array}{c}{n}_{I}\left(t\right)={\int }_{0}^{\infty }{P}_{I}\left(\tau \right)j\left(t-\tau \right)d\tau . \end{array}$$

Additionally, we consider the expected cumulative number of infections, ${n}_{T}\left(t\right)$, expressed in terms of the overall accumulated incidence as

13 $$\begin{array}{c}{n}_{T}\left(t\right)={\int }_{0}^{t}j\left(s\right)ds , \end{array}$$

and the dynamics of the expected cumulative deaths, ${n}_{D}\left(t\right)$,

14 $$\begin{array}{c}\frac{d}{dt}{n}_{D}\left(t\right)=IFR{\int }_{0}^{t}{f}_{OD}\left(t-l\right) {\int }_{0}^{l}{f}_{I}\left(l-s\right)j\left(s\right)dsdl, \end{array}$$

which takes into account that deaths occur with probability given by the infection fatality rate, $IFR$, at times after infection given by the convolution of the probability density functions of the incubation, ${f}_{I}$, and symptom onset-to-death, ${f}_{OD}$, times.

Similarly, the variation of the expected number of seropositive individuals at a time $t$, ${n}_{SP}\left(t\right)$, is expressed as

15 $$\begin{array}{c}\frac{d}{dt}{n}_{SP}\left(t\right)={\int }_{0}^{t}{f}_{SP}\left(t-s\right)j\left(s\right)ds,\end{array}$$

where ${f}_{SP}$ is the probability density function of the seroconversion time after infection, and the expected number of individuals with positive RT-PCR testing ${n}_{PT}(t)$, as

16 $$\begin{array}{c}{n}_{TP}\left(t\right)={\int }_{0}^{\infty }{P}_{TP}\left(\tau \right){u}_{I}\left(t,\tau \right)d\tau , \end{array}$$

where ${P}_{TP}\left(\tau \right)$ is the probability that an infected individual would test positive at a time $\tau$ after infection.

### Dynamical constraints

To obtain a closed set of equations for the different epidemiological quantities, we developed an approach to optimally simplify the convolutions. Explicitly, for the expressions involving an integral ${\int }_{0}^{\infty }A\left(\tau \right)j\left(t-\tau \right)d\tau$ of a function $A$ with the incidence $j$, we perform a series expansion of the incidence around the infection-age time ${\tau }_{A}$,

17 $$\begin{array}{c}j\left(t-\tau \right)=j\left(t-{\tau }_{A}\right)+{j^\prime}\left(t-{\tau }_{A}\right)\left({\tau }_{A}-\tau \right)+O\left({j^{\prime\prime}}\right),\end{array}$$

with the value of ${\tau }_{A}$ chosen as

18 $$\begin{array}{c}{\tau }_{A}=\frac{{\int }_{0}^{\infty }\tau A\left(\tau \right)d\tau }{{\int }_{0}^{\infty }A\left(\tau \right)d\tau } . \end{array}$$

The specific value of ${\tau }_{A}$ leads directly to a first-order approximation,

19 $$\begin{array}{c}{\int }_{0}^{\infty }A\left(\tau \right)j\left(t-\tau \right)d\tau =j\left(t-{\tau }_{A}\right){\int }_{0}^{\infty }A\left(\tau \right)d\tau +O\left({j^{\prime\prime}}\right),\end{array}$$

because ${\int }_{0}^{\infty }A\left(\tau \right)\left({\tau }_{A}-\tau \right)d\tau =0$ by the definition of ${\tau }_{A}$.

Using this approach, we obtain

20 $$\begin{array}{c}{n}_{I}\left(t\right)=j\left(t-\frac{{\tau }_{G}^{2}+{\sigma }_{G}^{2} }{2{\tau }_{G}}\right){\tau }_{G}+O\left({j^{\prime\prime}}\right) \end{array}$$

from Eq. (12), where ${\tau }_{G}={\int }_{0}^{\infty }\tau {f}_{GT}\left(\tau \right)d\tau$ and ${\sigma }_{G}^{2}={\int }_{0}^{\infty }{(\tau -{\tau }_{G})}^{2}{f}_{GT}\left(\tau \right)d\tau$ are the average and variance of the generation time, respectively, and

21 $$\begin{array}{c}\frac{d}{dt}{n}_{D}\left(t\right)=IFR j\left(t-{\tau }_{I}-{\tau }_{OD}\right)+O\left({j^{\prime\prime}}\right)\end{array}$$

from Eq. (14), where ${\tau }_{I}={\int }_{0}^{\infty }\tau {f}_{I}\left(\tau \right)d\tau$ and ${\tau }_{OD}={\int }_{0}^{\infty }\tau {f}_{OD}\left(\tau \right)d\tau$ are the incubation and symptom onset-to-death average times, respectively. These expressions lead straightforwardly to

22 $$\begin{array}{c}\frac{d}{dt}{n}_{D}\left(t\right)=\frac{IFR}{{\tau }_{G}} {n}_{I}\left(t+\frac{{\tau }_{G}^{2}+{\sigma }_{G}^{2} }{2{\tau }_{G}}-{\tau }_{I}-{\tau }_{OD}\right)\end{array}$$

and

23 $$\begin{array}{c}{n}_{D}\left(t\right)=IFR {n}_{T}\left(t-{\tau }_{I}-{\tau }_{OD}\right),\end{array}$$

up to $\mathcal{O}\left({j}^{{^{\prime}}{^{\prime}}}\right)$. Note that we have used ${\int }_{0}^{\infty }2\tau {P}_{I}\left(\tau \right)d\tau ={\int }_{0}^{\infty }d\left({\tau }^{2}{P}_{I}\left(\tau \right)\right)-{\int }_{0}^{\infty }{\tau }^{2}d{P}_{I}\left(\tau \right)={\int }_{0}^{\infty }{\tau }^{2}{f}_{GT}\left(\tau \right)d\tau$ and ${\int }_{0}^{\infty }{P}_{I}\left(\tau \right)d\tau ={\int }_{0}^{\infty }d\left(\tau {P}_{I}\left(\tau \right)\right)-{\int }_{0}^{\infty }\tau d{P}_{I}\left(\tau \right)={\int }_{0}^{\infty }\tau {f}_{GT}\left(\tau \right)d\tau$.

These expressions are used to estimate the infectious population ${n}_{I}(t)$ from the daily deaths, $\frac{d}{dt}{n}_{D}$, at time $t+{\tau }_{I}+{\tau }_{OD}-\frac{{\tau }_{G}^{2}+{\sigma }_{G}^{2} }{2{\tau }_{G}}$ and the cumulative infected population ${n}_{T}\left(t\right)$ from the cumulative deaths, ${n}_{D}$, at time $t+{\tau }_{I}+{\tau }_{OD}$, leading to

24 $$\begin{array}{c}{n}_{I}\left(t\right)=\frac{{\tau }_{G}}{IFR}\frac{d}{dt}{n}_{D}\left(t+{\tau }_{I}+{\tau }_{OD}-\frac{{\tau }_{G}^{2}+{\sigma }_{G}^{2} }{2{\tau }_{G}}\right),\end{array}$$

25 $$\begin{array}{c}{n}_{T}\left(t\right)=\frac{1}{IFR}{n}_{D}\left(t+{\tau }_{I}+{\tau }_{OD}\right).\end{array}$$

Similarly, we obtain

26 $$\begin{array}{c}\frac{d}{dt}{n}_{SP}\left(t\right)=j\left(t-{\tau }_{SP}\right)+O\left({j^{\prime\prime}}\right)\end{array}$$

27 $$\begin{array}{c}{n}_{TP}\left(t\right)=j\left(t-{\tau }_{TP}\right){\Delta t}_{TP}+O\left({j^{\prime\prime}}\right),\end{array}$$

where ${\tau }_{SP}$ is the average seroconversion time after infection and $\Delta {t}_{TP}$ is the average number of days an individual tests positive, which up to $\mathcal{O}\left({j^{\prime\prime}}\right)$ leads to

28 $$\begin{array}{c}{n}_{SP}\left(t\right)={n}_{T}\left(t-{\tau }_{SP}\right),\end{array}$$

29 $$\begin{array}{c}\frac{d}{dt}{n}_{D}\left(t\right)=\frac{IFR}{\Delta {t}_{TP}}{n}_{TP}\left(t+{\tau }_{TP}-{\tau }_{I}-{\tau }_{OD}\right).\end{array}$$

Combining Eqs. ($28$) and ($29$) with Eqs. ($24$) and ($25$) leads to

30 $$\begin{array}{c}{n}_{I}\left(t\right)=\frac{{\tau }_{G}}{\Delta {t}_{TP}}{n}_{TP}\left(t+{\tau }_{TP}-\frac{{\tau }_{G}^{2}+{\sigma }_{G}^{2} }{2{\tau }_{G}}\right),\end{array}$$

31 $$\begin{array}{c}{n}_{T}\left(t\right)={n}_{SP}\left(t+{\tau }_{SP}\right),\end{array}$$

which is used to validate the values of the estimated infectious population ${n}_{I}(t)$ from RT-PCR testing results, ${n}_{TP}$, at time $t+{\tau }_{TP}-\frac{{\tau }_{G}^{2}+{\sigma }_{G}^{2} }{2{\tau }_{G}}$ and the cumulative infected population ${n}_{T}\left(t\right)$ from seropositivity testing, ${n}_{SP}$, at time $t+{\tau }_{SP}$.

### Expected deaths

The raw cumulative death counts over time, ${n}_{W}\left(t\right)$, are obtained from the Johns Hopkins University Center for Systems Science and Engineering^1^ for countries and for US locations.

The daily death counts $\Delta {n}_{W}\left(t\right)={n}_{W}\left(t\right)-{n}_{W}\left(t-1\right)$ are considered to contain reporting artifacts if they are negative or if they are unrealistically large. This last condition is defined explicitly as larger than 4 times its previous 14-day average value plus 10 deaths, $\Delta {n}_{W}\left(t\right)>10+4\times \frac{1}{14}\left({n}_{W}\left(t\right)-{n}_{W}\left(t-14\right)\right)$, from a non-sparse reporting schedule with at least 2 consecutive non-zero values before and after the time $t$, $\Delta {n}_{W}\left(t\right)\ne \frac{1}{5}\left({n}_{W}\left(t+2\right)-{n}_{W}\left(t-3\right)\right)$.

Reporting artifacts identified at time $t$ are considered to be the result of previous miscounting. The excess or lack of deaths are imputed proportionally to previous death counts. Explicitly, death counts are updated as

32 $$\begin{array}{c}{n}_{W}\left(t-1-i\right)\leftarrow {n}_{W}\left(t-1-i\right)\frac{{n}_{W}{\left(t-1\right)}_{estimated}}{{n}_{W}\left(t-1\right)}\end{array}$$

with ${n}_{W}{\left(t-1\right)}_{estimated}={n}_{W}\left(t\right)-\frac{1}{7}\left({n}_{W}\left(t-1\right)-{n}_{W}\left(t-8\right)\right)$ for all $i\ge 0$. In this way, $\Delta {n}_{W}\left(t\right)$ is assigned its previous seven-day average value.

The expected daily deaths, $\Delta {n}_{D}(t)$, are obtained through a density estimation multiscale functional, ${f}_{de}$, as $\Delta {n}_{D}(t)={f}_{de}\left(\Delta {n}_{W}\left(t\right)\right)$, which leads to the estimation of the expected cumulative deaths at time $t$ as ${n}_{D}\left(t\right)={n}_{W}\left({t}_{0}\right)+{\sum }_{s={t}_{0}+1}^{t}\Delta {n}_{D}(s)$. Specifically,

33 $$\begin{array}{c}{f}_{de}\left(\Delta {n}_{W}\left(t\right)\right)=\left(1-{r}_{1}\right)d{d}_{0}+{r}_{1}\left(\left(1-{r}_{2}\right)d{d}_{1}+{r}_{2}d{d}_{2}\right)\end{array}$$

with

34 $$\begin{array}{c}{r}_{1} = {e}^{-0.3d{d}_{1}},\end{array}$$

35 $$\begin{array}{c}{r}_{2} = {e}^{-3d{d}_{2}},\end{array}$$

36 $$\begin{array}{c}d{d}_{0}={ma}_{14}\left({ma}_{14}\left(\Delta {n}_{W}\left(t\right)\right)\right),\end{array}$$

37 $$\begin{array}{c}d{d}_{1}={rg}_{12}\left({ma}_{14}\left(\Delta {n}_{W}\left(t\right)\right)\right),\end{array}$$

38 $$\begin{array}{c}d{d}_{2}={rg}_{48}\left({ma}_{14}\left(\Delta {n}_{W}\left(t\right)\right)\right),\end{array}$$

where ${ma}_{14}\left(\cdot \right)$ is a centered moving average with window size of 14 days and ${rg}_{\sigma }\left(\cdot \right)$ is a centered rolling average through a Gaussian window with standard deviation $\sigma$. The specific value of the window size has been chosen to mitigate weekly reporting effects. The values of the standard deviations of the Gaussian windows have been selected to achieve a smooth representation of the expected death estimation for each country as shown in the bottom panels of Supplementary Fig. S1.

### Reporting delays

We consider an average delay of two days between reporting a death and its occurrence. This value is obtained by comparing the daily death counts reported for Spain^1^ and their actual values^33^ from February 15 to March 31, 2020. The values of the root-mean-squared deviation between reported and actual deaths shifted by 0, 1, 2, 3, and 4 days are 77.9, 58.4, 38.5, 58.7, and 88.6 deaths respectively.

### Infection fatality rate ($IFR$)

The infection fatality rate is computed assuming homogeneous attack rate as

39 $$\begin{array}{c}IFR=\frac{1}{{\sum }_{a}{g}_{a}}{\sum }_{a}{IFR}_{a}{g}_{a} ,\end{array}$$

where ${\mathrm{IFR}}_{a}$ is the previously estimated $IFR$ for the age group $a$^5^ and ${g}_{a}$ is the population in the age group $a$ as reported by the United Nations for countries^18^ and the US Census for states^19^.

### Clinical parameters

We obtained the values of the average ${\tau }_{G}$ and standard deviation ${\sigma }_{G}$ of the generation time from Ref.^13^, of the averages of the incubation ${\tau }_{I}$ and symptom onset-to-death ${\tau }_{OD}$ times from Refs.^5,14^, and of the average number of days $\Delta {t}_{TP}$ of positive testing by an infected individual from Refs.^15,17^. The average time at which an individual tested positive after infection ${\tau }_{TP}$ was computed as ${\tau }_{TP}={\tau }_{I}-2+\Delta {t}_{TP}/2$, where we have assumed that on average an individual started to test positive 2 days before symptom onset. The average seroconversion time after infection ${\tau }_{SP}$ was estimated as ${\tau }_{I}$ plus the 7 days of 50% seroconversion after symptom onset reported in Ref.^16^.

### Dynamical constraints implementation with discrete time

We implemented the dynamical constraints to compute the infectious and infected population as outlined in the main text and as detailed in the previous section of this document, using days as time units. Time delays were rounded to days to assign daily values.

The first derivative of the cumulative number of deaths is computed as

40 $$\begin{array}{c}\frac{d{n}_{D}\left(t\right)}{dt}=\Delta {n}_{D}\left(t\right),\end{array}$$

with $$\Delta {n}_{D}\left(t\right)={n}_{D}\left(t\right)-{n}_{D}(t-1)$$.

The growth rate was computed explicitly from the discrete time series as the centered 7-day difference

41 $$\begin{array}{c}{k}_{G}\left(t\right)=\frac{1}{7}\left({\mathrm{ln}}\left(\Delta {n}_{D}\left(t+4\right)+\Delta {n}_{D}\left(t+3\right)\right)-{\mathrm{ln}}\left(\Delta {n}_{D}\left(t-3\right)+\Delta {n}_{D}\left(t-4\right)\right)\right).\end{array}$$

The 7-day value was chosen to mitigate reporting artifacts.

### Confidence and credibility intervals

Confidence intervals associated with death counts were computed using bootstrapping with 10,000 realizations^34^. These confidence intervals were combined with the credibility intervals of the $IFR$ in infectious and infected populations assuming independence and additivity on a logarithmic scale.

### Fold accuracy

The fold accuracy, ${F}_{A}$, is explicitly computed as

42 $$\begin{array}{c}{\mathrm{log}}{F}_{A}=\frac{1}{N}{\sum }_{i=1}^{N}\left|{\mathrm{log}}{x}_{i}^{obs}-{\mathrm{log}}{x}_{i}^{est}\right|,\end{array}$$

where $\left|\cdot \right|$ is the absolute value function, ${x}_{i}^{obs}$ is the ${i}^{th}$ observation, ${x}_{i}^{est}$ is its corresponding estimation, and $N$ is the total number of observations.

### Inference and extrapolation

Because of the delay between infections and deaths, inference for the values of the growth rate and infectious populations ends on December 30, 2020 and for the values of the infected populations ends on December 26, 2020. Extrapolation to the current time (January 21, 2021) is carried out assuming the last growth rate computed.

### Reproduction number

The quantities ${R}_{t}$ and ${k}_{G}\left(t\right)$ are related to each other through the Euler–Lotka equation, ${R}_{t}^{-1}={\int }_{0}^{\infty }{f}_{GT}\left(\tau \right){e}^{-{k}_{G}\left(t\right)\tau }d\tau ,$ which considers $j\left(t-\tau \right)\simeq {e}^{-{k}_{G}\left(t\right)\tau }j\left(t\right)$ in the renewal equation $j\left(t\right)={\int }_{0}^{\infty }{k}_{I}\left(t,\tau \right)j\left(t-\tau \right)d\tau$. Generation times can generally be described through a gamma distribution ${f}_{GT}\left(\tau \right)=\frac{{\beta }^{\alpha }}{\Gamma \left(\alpha \right)}{\tau }^{\alpha -1}{e}^{-\beta \tau }$ with $\alpha ={\tau }_{G}^{2}/{\sigma }_{G}^{2}$ and $\beta ={\tau }_{G}/{\sigma }_{G}^{2}$, which leads to ${R}_{t}={\left(1+{k}_{G}(t)/\beta \right)}^{\alpha }$ for ${k}_{G}(t)>-\beta$ and ${R}_{t}=0$ for ${k}_{G}\left(t\right)\le -\beta$. In the case of the exponentially distributed limit ($\alpha \simeq 1$) or small values of ${k}_{G}(t)/\beta$, it simplifies to ${R}_{t}=1+{k}_{G}\left(t\right){\tau }_{G}$ for ${k}_{G}\left(t\right)>-1/{\tau }_{G}$ and ${R}_{t}=0$ for ${k}_{G}\left(t\right)\le -1/{\tau }_{G}$. Global prevalence data were obtained from multiple data sources^35–42^, as described in Supplementary Table S1.

## Supplementary Information

Supplementary Information.
