# Supplementary material for: Reliably quantifying the evolving worldwide dynamic state of the COVID-19 outbreak from death records, clinical parametrization, and demographic data
Source: Sci Rep. 2021 Oct 7;11:19952. doi: 10.1038/s41598-021-99273-1 (PMC8497510; doi:10.1038/s41598-021-99273-1)
Supplement: Supplementary file 1 — Supplementary Information. [file 41598_2021_99273_MOESM1_ESM.pdf]

SUPPLEMENTARY INFORMATION: Reliably quantifying the evolving worldwide dynamic state of the COVID-19 outbreak from death records, clinical parametrization, and demographic data

Jose M. G. Vilar[1,2] and Leonor Saiz[3]

[1] Biofisika Institute (UPV/EHU, CSIC), University of the Basque Country (UPV/EHU), P.O. Box 644, 48080 Bilbao, Spain

[2] IKERBASQUE, Basque Foundation for Science, 48011 Bilbao, Spain

[3] Department of Biomedical Engineering, University of California, 451 E. Health Sciences Drive, Davis, CA 95616, USA

Supplementary references

References for the supplementary information are provided in the main text of the manuscript.

Supplementary table

Table S1. Sources for the prevalence data at a global scale. The sources include press releases, online preprints, and publications. Seroprevalence data for Belgium and Spain does not include late estimates that show clear effects of diminishing antibody levels after infection. Prevalence data for England was used as a proxy for the United Kingdom. One of the data points for France [35] is a theoretical estimate based on detailed data.

Location	Data source
**Austria**	'https://www.sora.at/nc/news-presse/news/news-einzelansicht/news/covid-19-praevalenz-1006.html'
**Belgium**	[28]
**Brazil**	[36]
**Denmark**	[26] and 'https://files.ssi.dk/Forelobige resultater fra seropraevalensundersogelse af COVID19-20052020'
**Greece**	[37]
**France**	[35] and [38]
**Indiana State**	[39] and 'https://news.iu.edu/stories/2020/06/iupui/releases/17-fairbanks-isdh-second-phase-covid-19-testing-indiana-research.html'
**Netherlands**	[40]
**New York State**	'https://www.governor.ny.gov/news/amid-ongoing-covid-19-pandemic-governor-cuomo-announces-results-completed-antibody-testing'
**Norway**	'https://www.fhi.no/en/publ/2020/seroprevalence-of-sars-cov-2-in-the-norwegian-population--measured-in-resid'
**Oregon State**	[41]
**Spain**	[25] and 'https://www.mscbs.gob.es/ciudadanos/ene-covid/home.htm'
**Sweden**	'http://outbreaknewstoday.com/sweden-releases-results-of-covid-19-prevalence-investigation-76786/'
**United Kingdom**	'https://www.ons.gov.uk/peoplepopulationandcommunity/healthandsocialcare/conditionsanddiseases/bulletins/coronaviruscovid19infectionsurveypilot/28may2020'
**United States**	[20]

Supplementary figure

(The 70-page long figure is appended after the caption)

Fig. S1. Characterization of the temporal evolution of the COVID-19 outbreak for each of the countries in the World and states and territories in the US. The figure includes 208 locations. The data for each location is shown in a column of 5 panels with its name on the top.

The 1st panel from the top shows the trajectory in the growth rate-infectious population space. Each day is indicated by a symbol increasing in size with time. The largest symbol corresponds to the last day of the estimation (December 30, 2020). The blue line at the end indicates the extrapolation to the current time (January 21, 2021) assuming for the growth rate its last estimated value.

The 2nd panel from the top shows the temporal evolution of the growth rate (orange circles) and its extrapolation (blue line without circles). The shaded blue region indicates the 95% confidence intervals (CI).

The 3rd panel from the top shows the temporal evolution of the infectious population (orange circles) and its extrapolation (blue line without circles). The shaded blue region indicates the 95% CI assuming a certain IFR. The dashed lines indicate the overall 95% credibility intervals (CrI) taking into account the uncertainty in the estimates of the IFR.

The 4th panel from the top shows the temporal evolution of the infected population (orange circles) and its extrapolation (blue line without circles). The shaded blue region indicates the 95% CI assuming a certain IFR. The dashed lines indicate the overall 95% CrI taking into account the uncertainty in the estimates of the IFR.

The 5th panel from the top shows the raw reported daily deaths (blue circles), the processed daily deaths to mitigate reporting artifacts (orange circles), and the expected deaths (black curve).

Countries are arranged in alphabetical order. Locations in the US are arranged in alphabetical order after the data for the "United States" as a country. The prefix "US " has been added to the name of the locations in the US. The analyses for the locations "World" and "United States" have been computed with their overall deaths and demographics in contrast to Fig. 3 in the main text, which considered the cumulative contributions of countries and states. Death counts for Spain between April 1 and November 4, 2020 were obtained from "https://www.mscbs.gob.es" [33] because of missing updates in JHU CSSE COVID-19 data [1]. Death counts for China before January 22, not present in JHU CSSE COVID-19 data [1], were obtained from the European Centre for Disease Prevention and Control [42].

The following locations were not included in the analysis because of lack of demographic information in the United Nations or US Census data: Andorra, Diamond Princess, Dominica, Holy See, Kosovo, Liechtenstein, Marshall Islands, Monaco, MS Zaandam, Saint Kitts and Nevis, Samoa, San Marino, US Diamond Princess, US Grand Princess, US Northern Mariana Islands, and US Virgin Islands.

The following locations were not included in the analysis because they did not have at least 30 reported COVID-19 deaths: Antigua and Barbuda, Barbados, Bhutan, Brunei, Burundi, Cambodia, Eritrea, Fiji, Grenada, Iceland, Laos, Mauritius, Micronesia, Mongolia, New Zealand, Papua New Guinea, Saint Lucia, Saint Vincent and the Grenadines, Sao Tome and Principe, Seychelles, Singapore, Solomon Islands, Taiwan, Tanzania, Timor-Leste, US American Samoa, and Vanuatu.

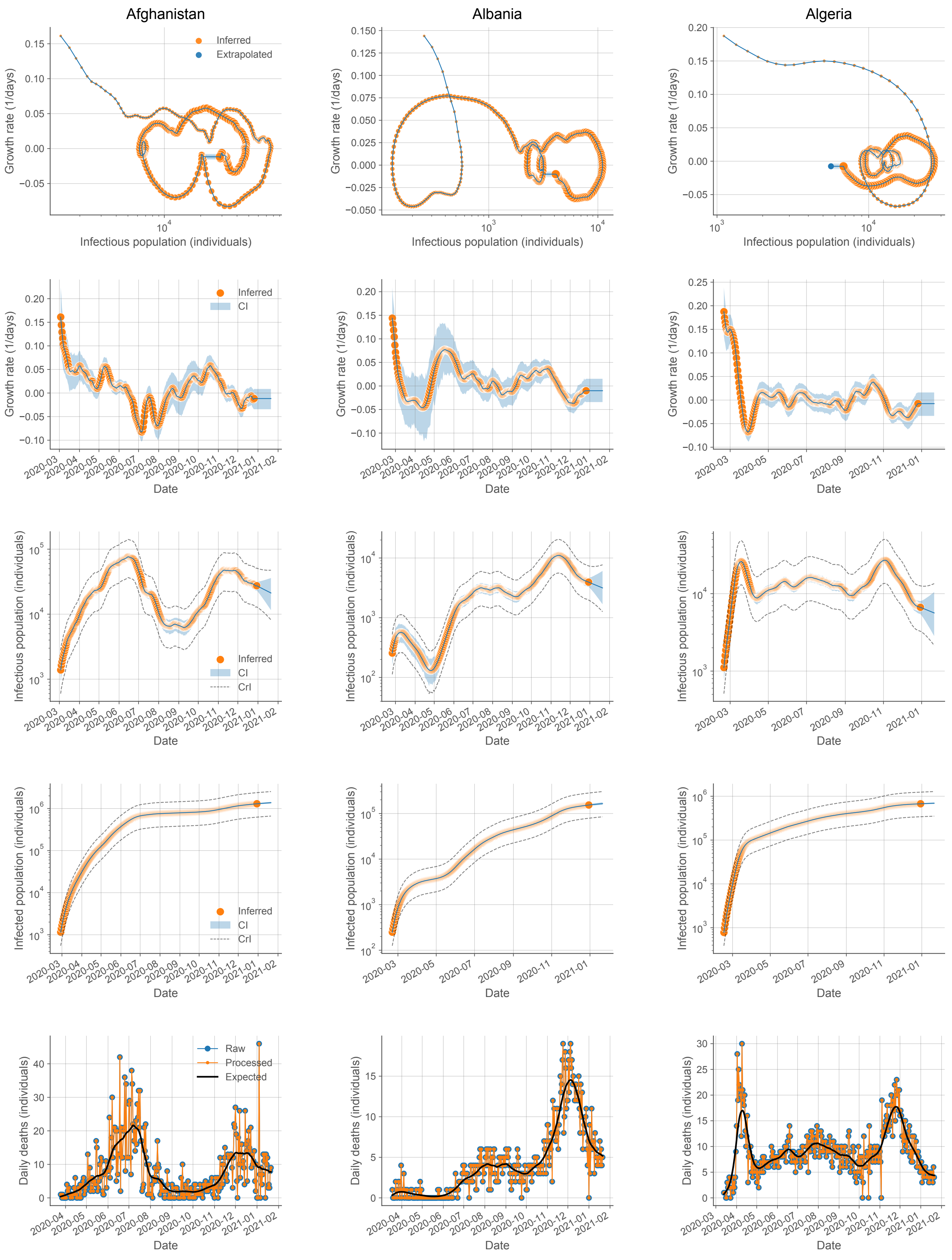

Afghanistan
Albania
Algeria
Growth rate (1/days)
Infectious population (individuals)
Inferred
Extrapolated
Date
CI
CrI
Infected population (individuals)
Daily deaths (individuals)
Raw
Processed
Expected

Figure S1.1

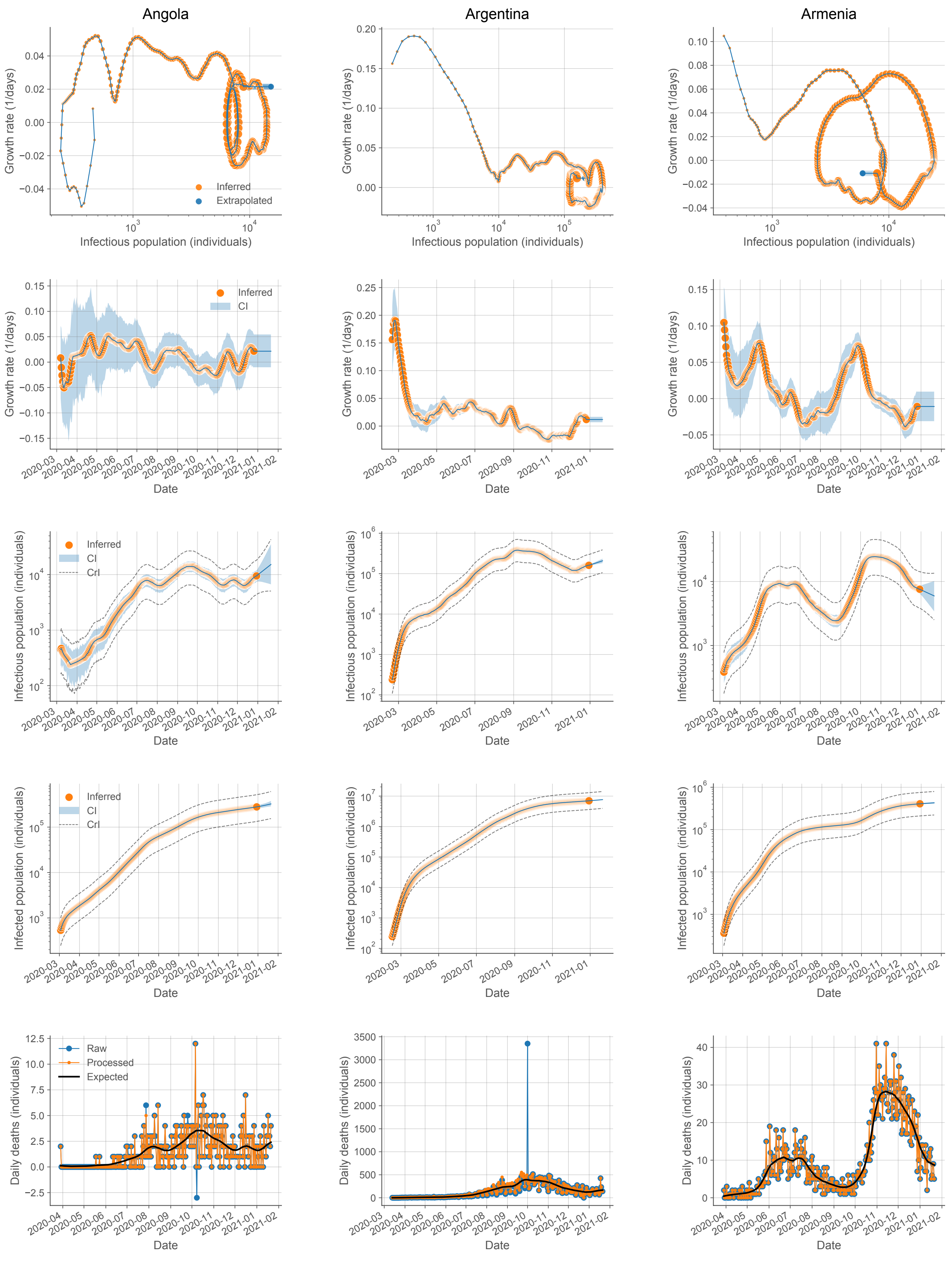

Angola
Argentina
Armenia
Growth rate (1/days)
Infectious population (individuals)
Inferred
Extrapolated
CI
CrI
Date
Infected population (individuals)
Daily deaths (individuals)
Raw
Processed
Expected

Figure S1.2

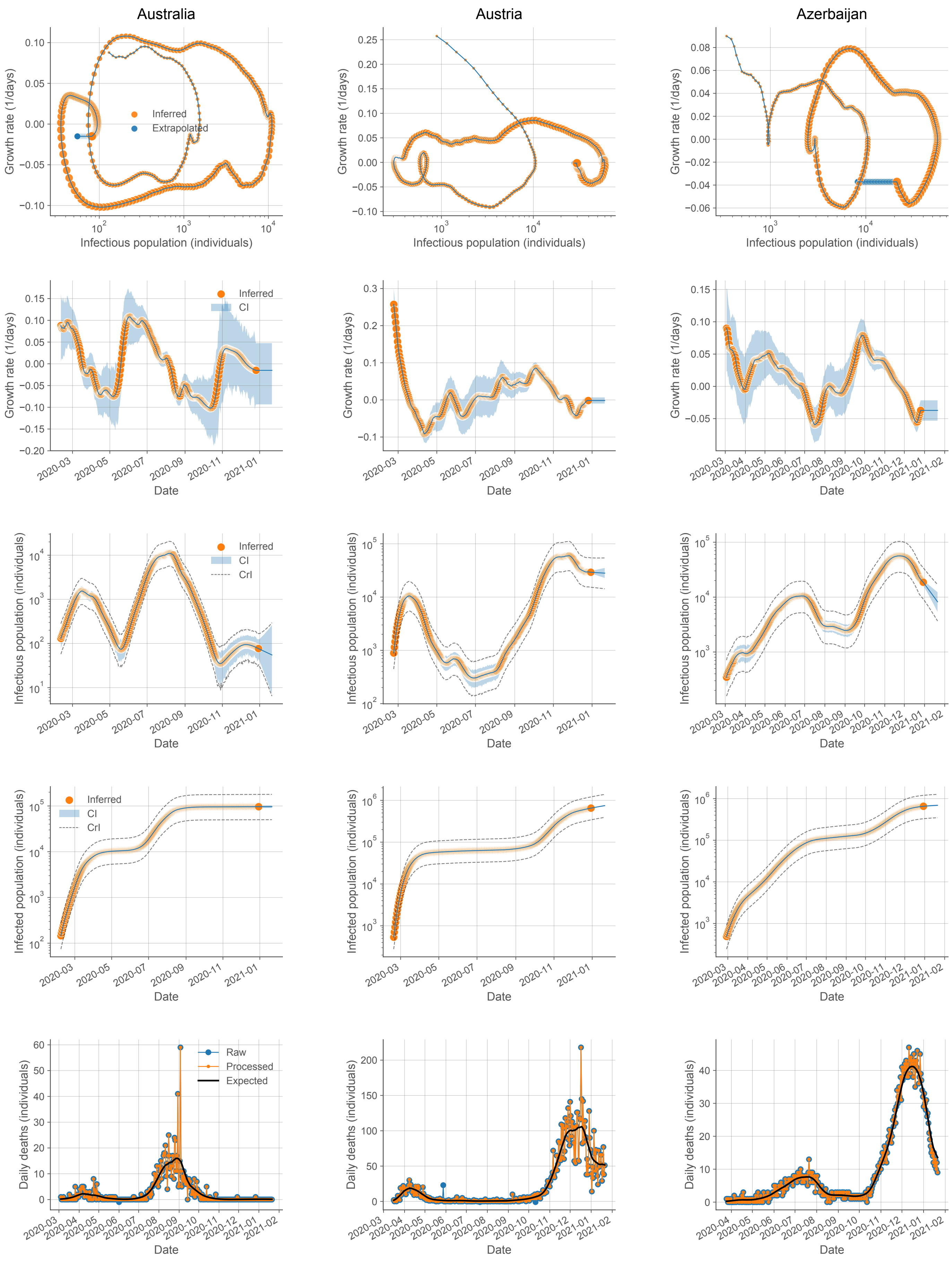
Australia
Austria
Azerbaijan
Growth rate (1/days)
Infectious population (individuals)
Inferred
Extrapolated
CI
CrI
Date
Infected population (individuals)
Daily deaths (individuals)
Raw
Processed
Expected

Figure S1.3

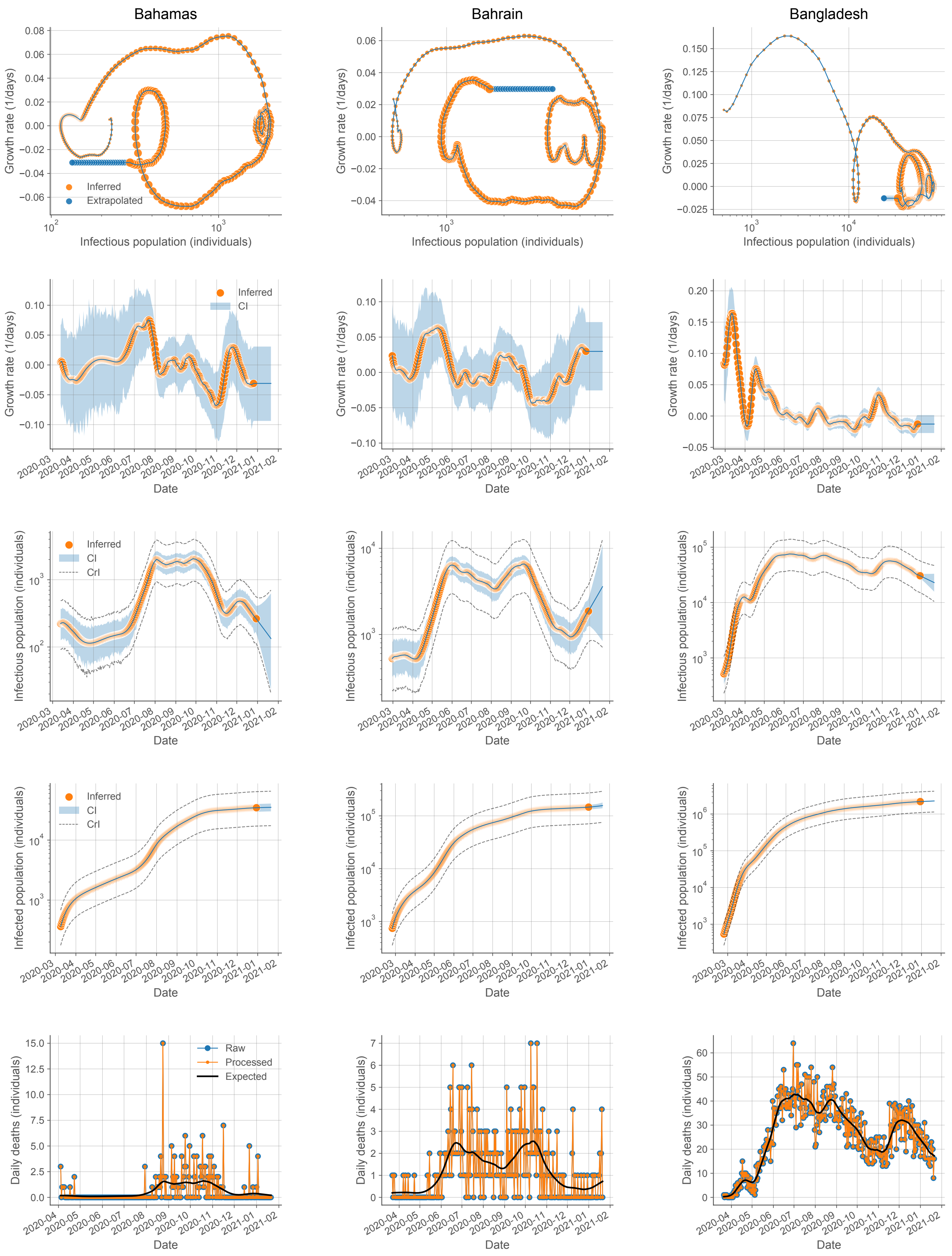
Bahamas
Bahrain
Bangladesh
Growth rate (1/days)
Infectious population (individuals)
Inferred
Extrapolated
CI
CrI
Date
Infected population (individuals)
Daily deaths (individuals)
Raw
Processed
Expected

Figure S1.4

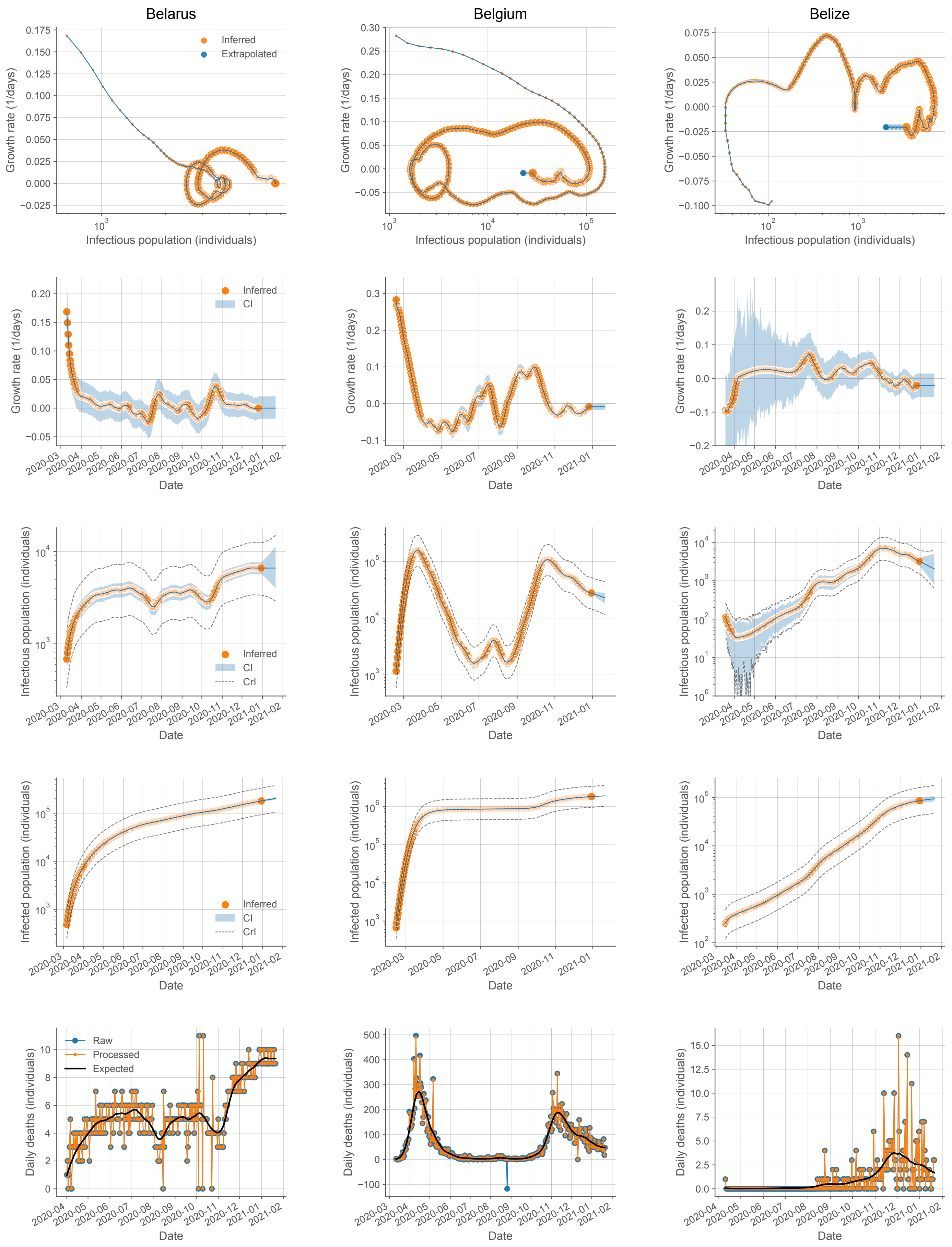

Belarus
Belgium
Belize
Growth rate (1/days)
Infectious population (individuals)
Inferred
Extrapolated
CI
CrI
Date
Infected population (individuals)
Daily deaths (individuals)
Raw
Processed
Expected

Figure S1.5

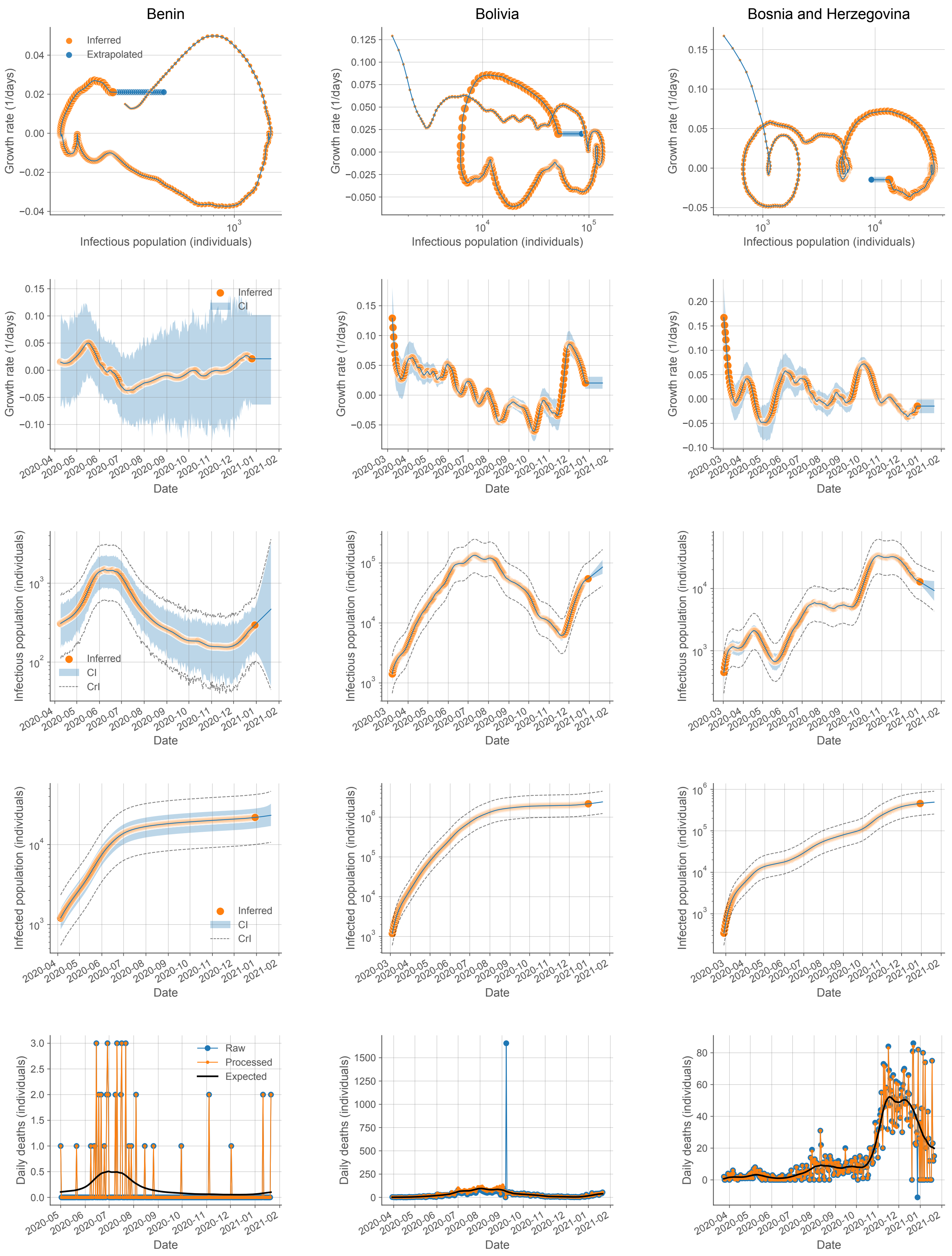
Benin
Bolivia
Bosnia and Herzegovina
Growth rate (1/days)
Infectious population (individuals)
Infected population (individuals)
Daily deaths (individuals)
Date
Inferred
Extrapolated
CI
CrI
Raw
Processed
Expected

Figure S1.6

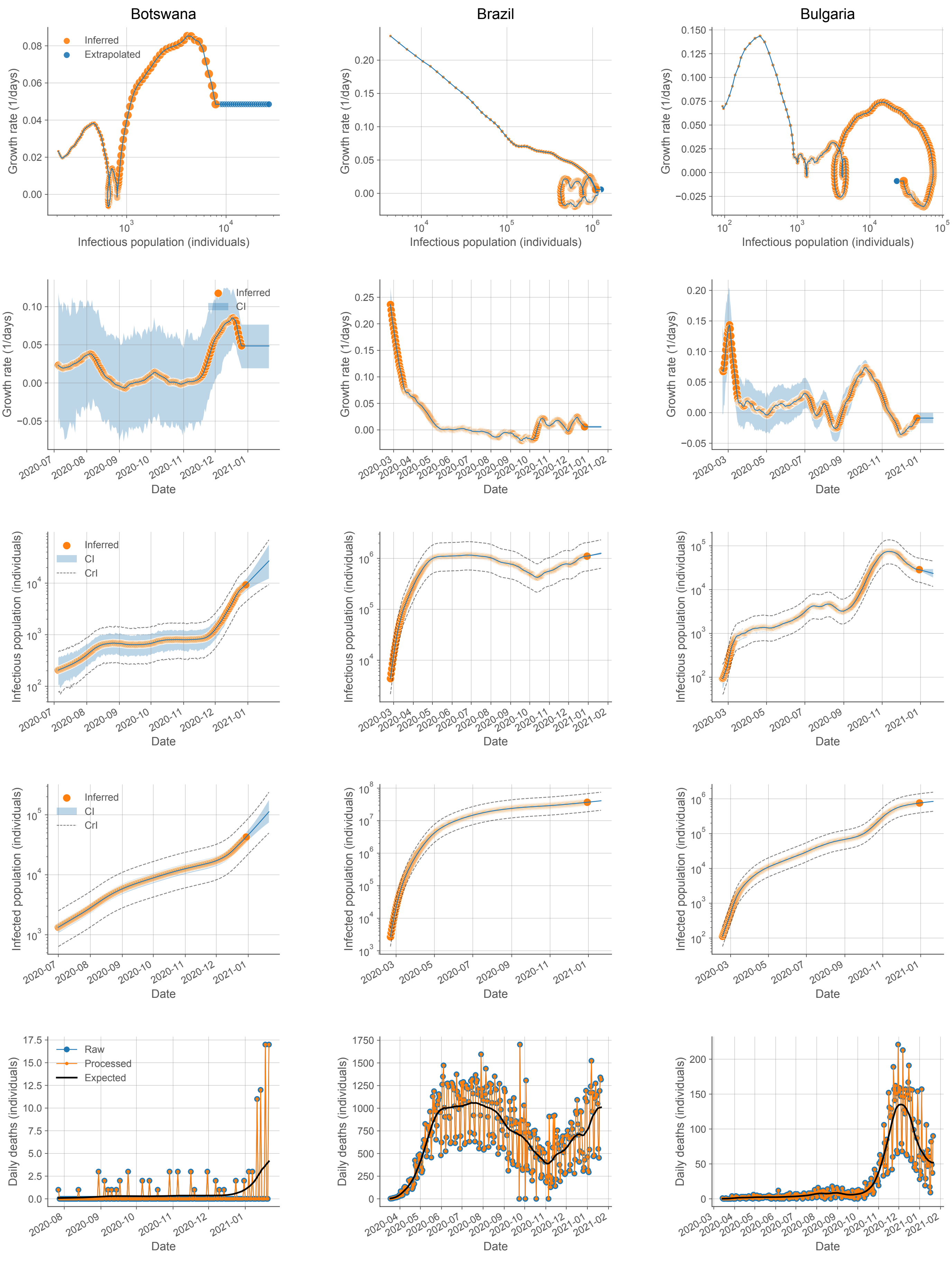

Botswana
Brazil
Bulgaria
Inferred
Extrapolated
CI
CrI
Raw
Processed
Expected
Growth rate (1/days)
Infectious population (individuals)
Infected population (individuals)
Daily deaths (individuals)
Date

Figure S1.7

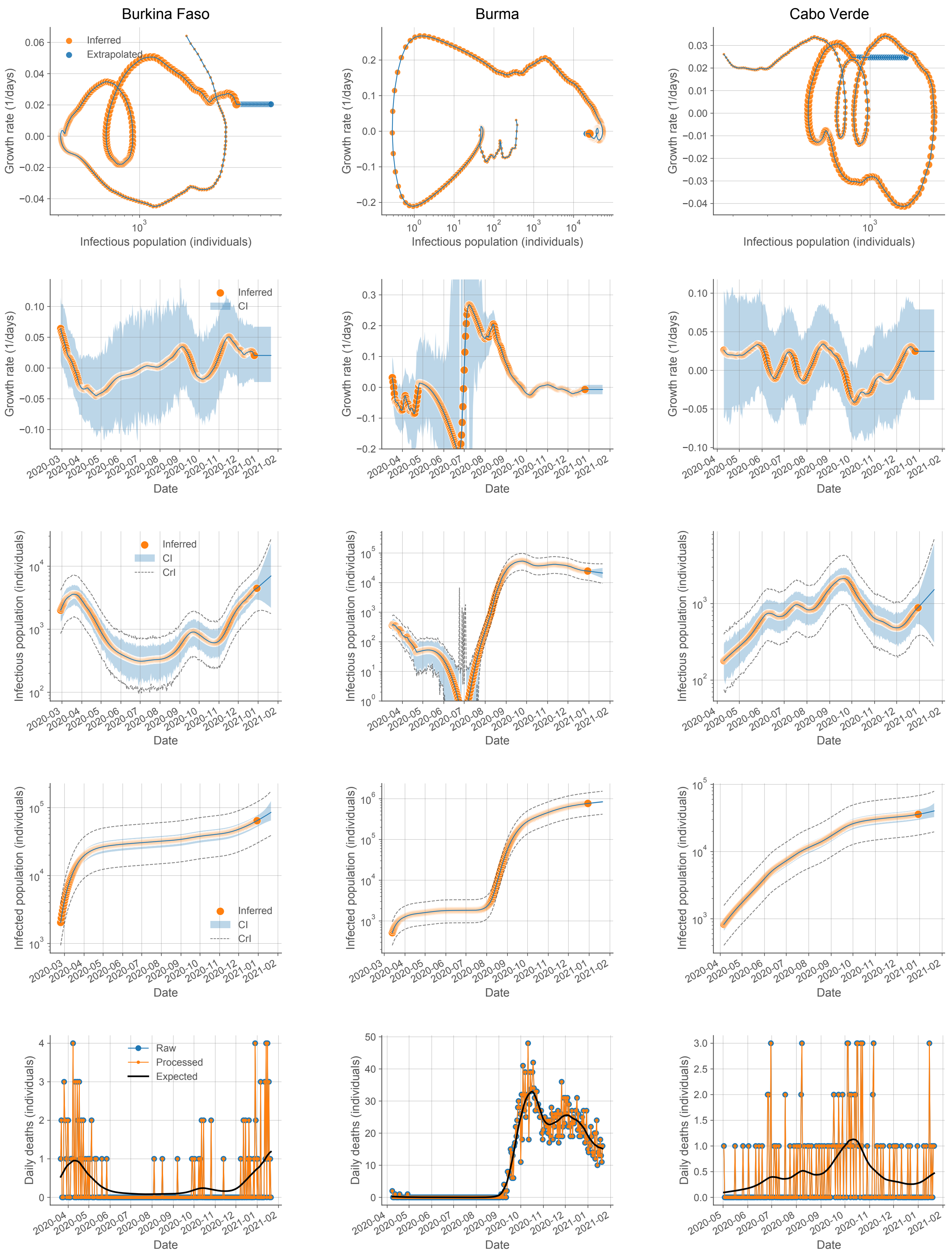
Burkina Faso
Burma
Cabo Verde
Inferred
Extrapolated
CI
CrI
Raw
Processed
Expected
Growth rate (1/days)
Infectious population (individuals)
Infected population (individuals)
Daily deaths (individuals)
Date

Figure S1.8

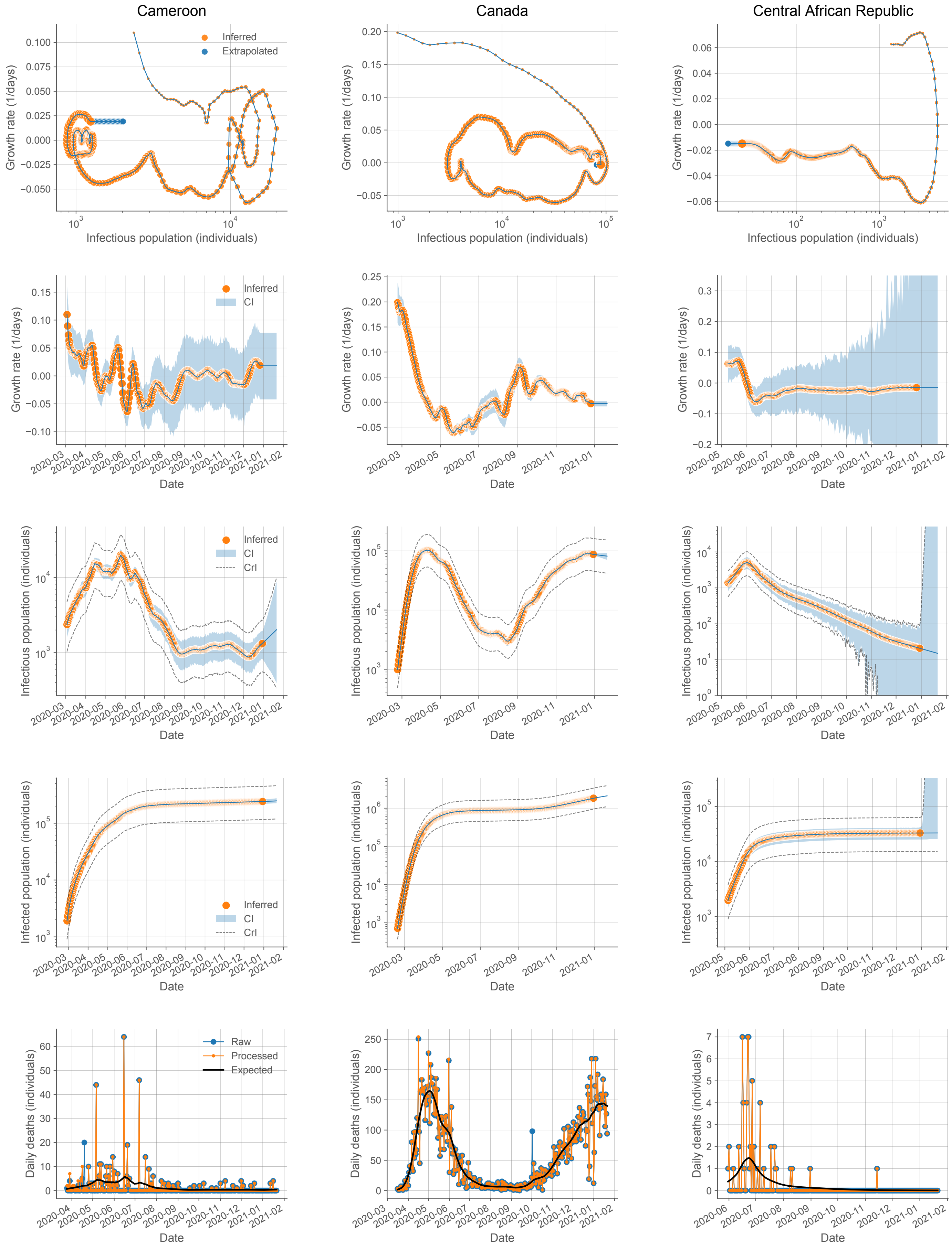

Cameroon
Canada
Central African Republic
Inferred
Extrapolated
CI
CrI
Raw
Processed
Expected
Growth rate (1/days)
Infectious population (individuals)
Infected population (individuals)
Daily deaths (individuals)
Date

Figure S1.9

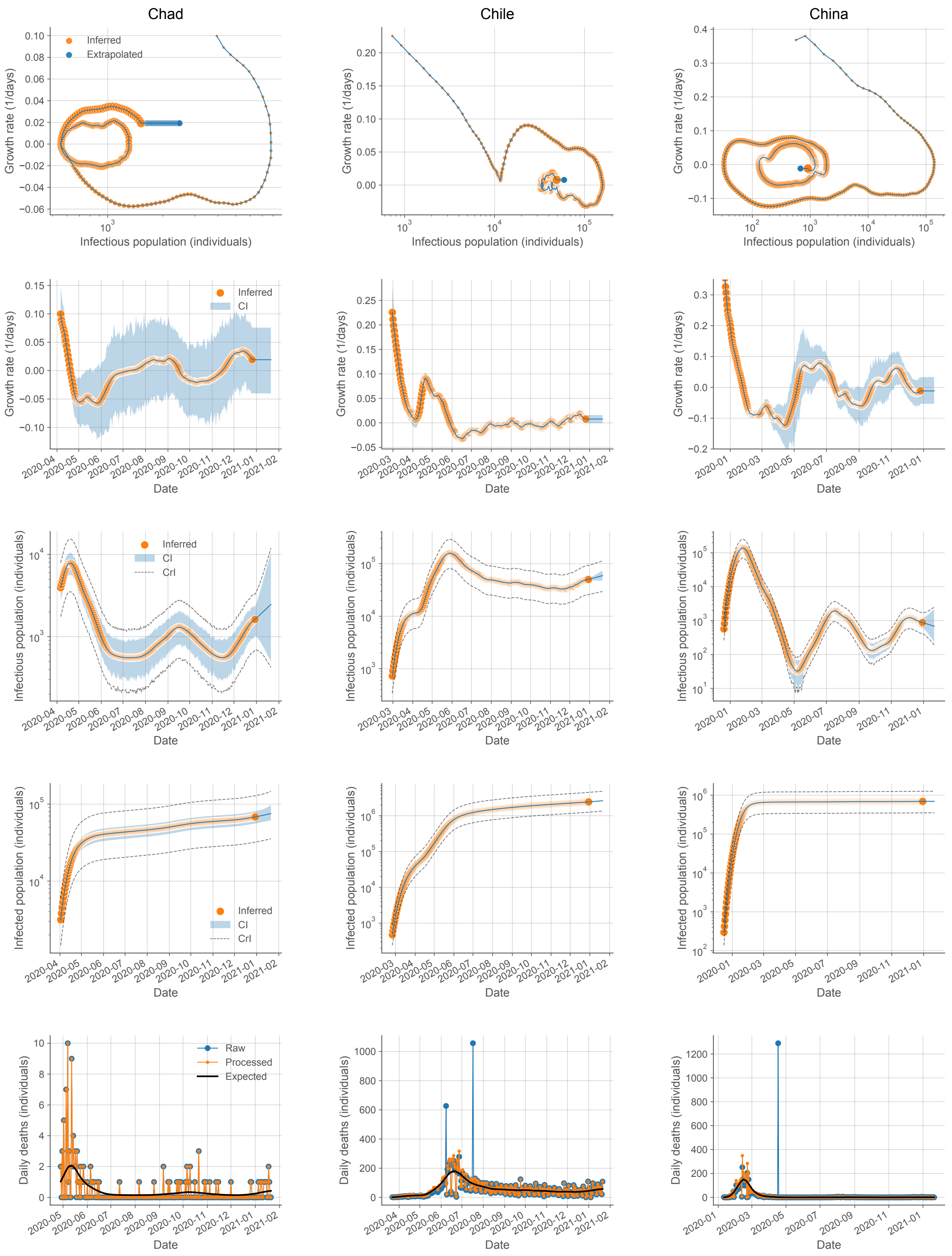

Chad
Chile
China
Growth rate (1/days)
Infectious population (individuals)
Inferred
Extrapolated
CI
CrI
Date
Infected population (individuals)
Daily deaths (individuals)
Raw
Processed
Expected

Figure S1.10

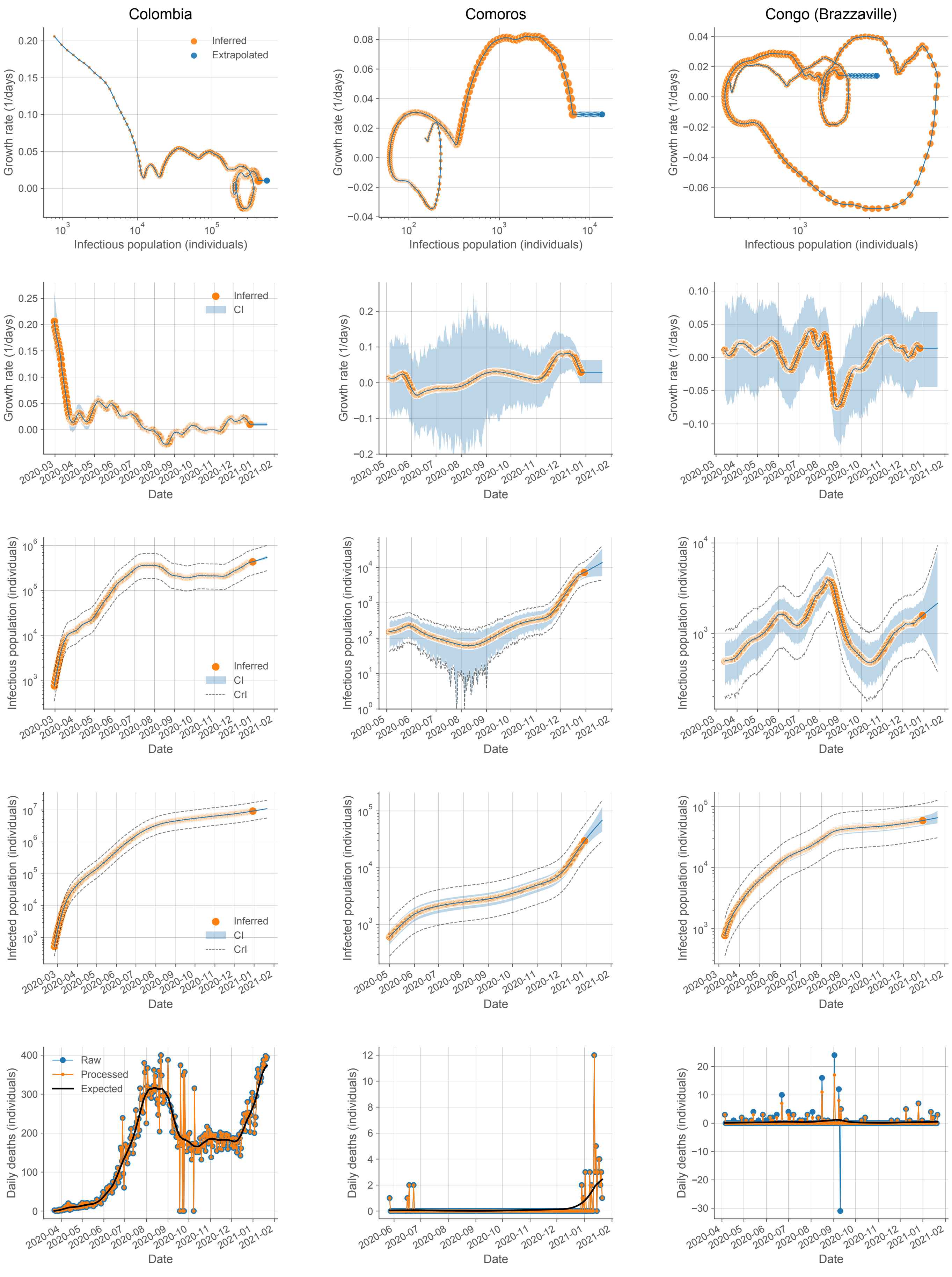

Colombia
Comoros
Congo (Brazzaville)
Growth rate (1/days)
Infectious population (individuals)
Inferred
Extrapolated
CI
CrI
Date
Infected population (individuals)
Daily deaths (individuals)
Raw
Processed
Expected

Figure S1.11

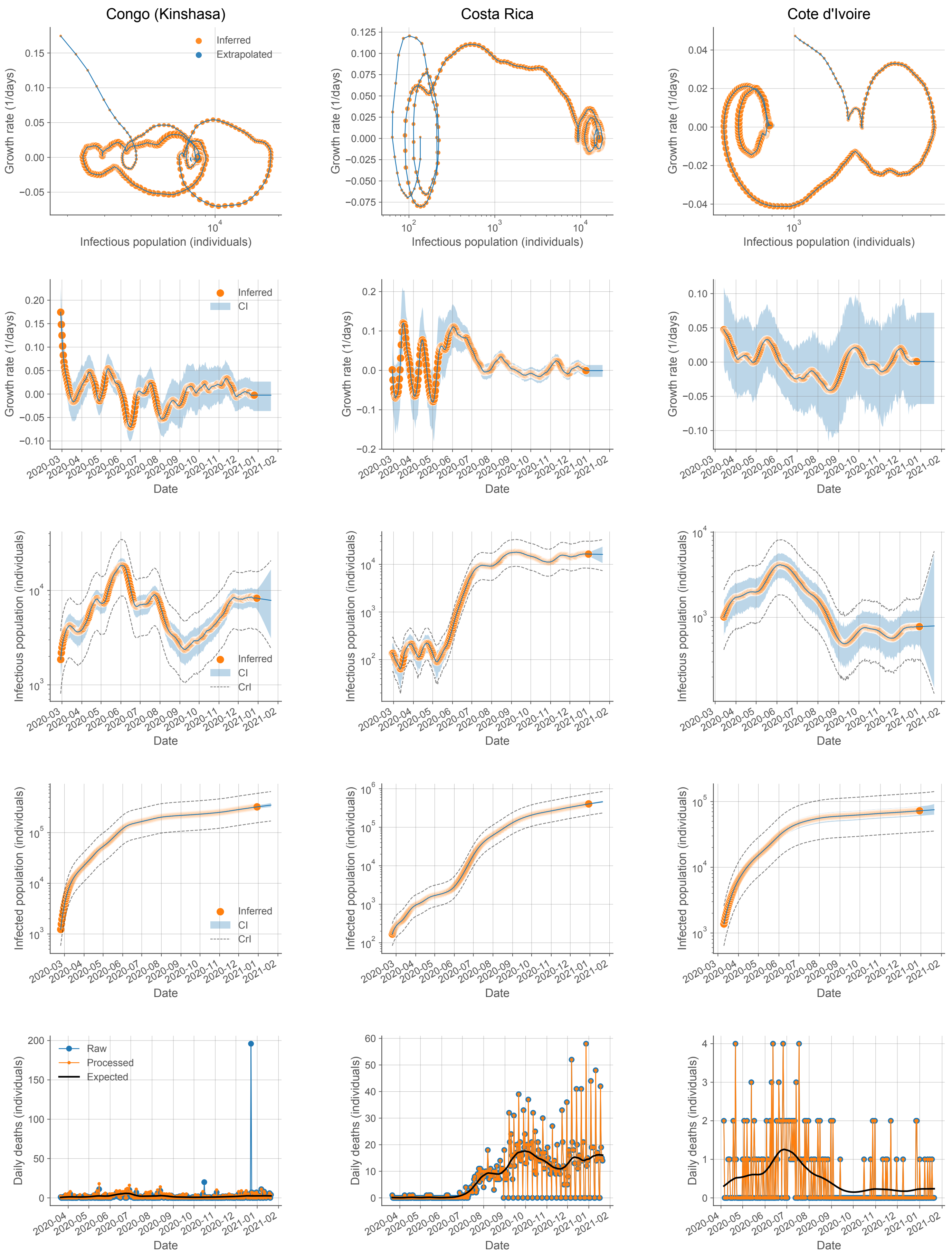

Congo (Kinshasa)
Costa Rica
Cote d'Ivoire
Growth rate (1/days)
Infectious population (individuals)
Inferred
Extrapolated
Growth rate (1/days)
Date
Inferred
CI
Infectious population (individuals)
Date
Inferred
CI
CrI
Infected population (individuals)
Date
Inferred
CI
CrI
Daily deaths (individuals)
Date
Raw
Processed
Expected

Figure S1.12

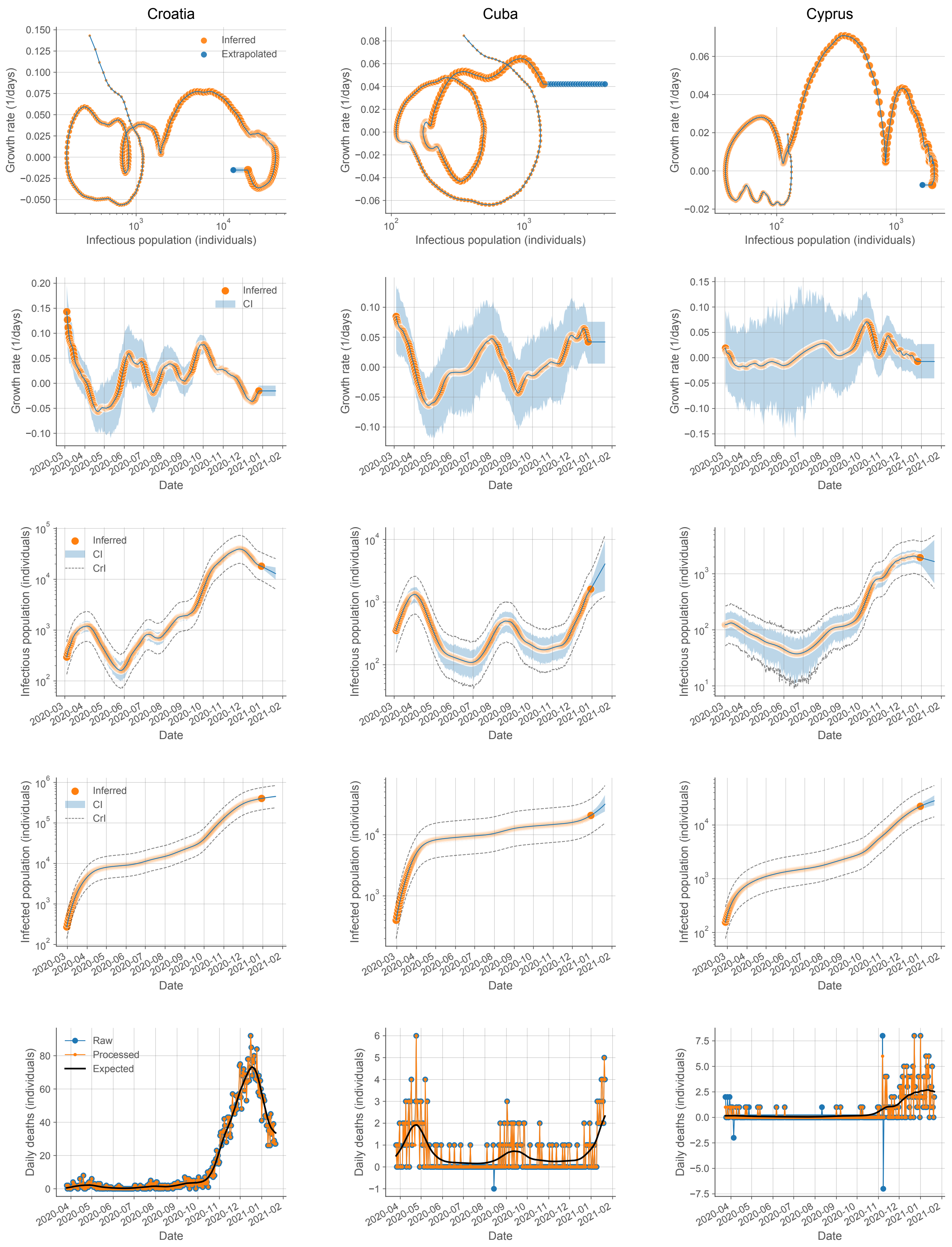
Croatia
Cuba
Cyprus
Growth rate (1/days)
Infectious population (individuals)
Inferred
Extrapolated
CI
CrI
Date
Infected population (individuals)
Daily deaths (individuals)
Raw
Processed
Expected

Figure S1.13

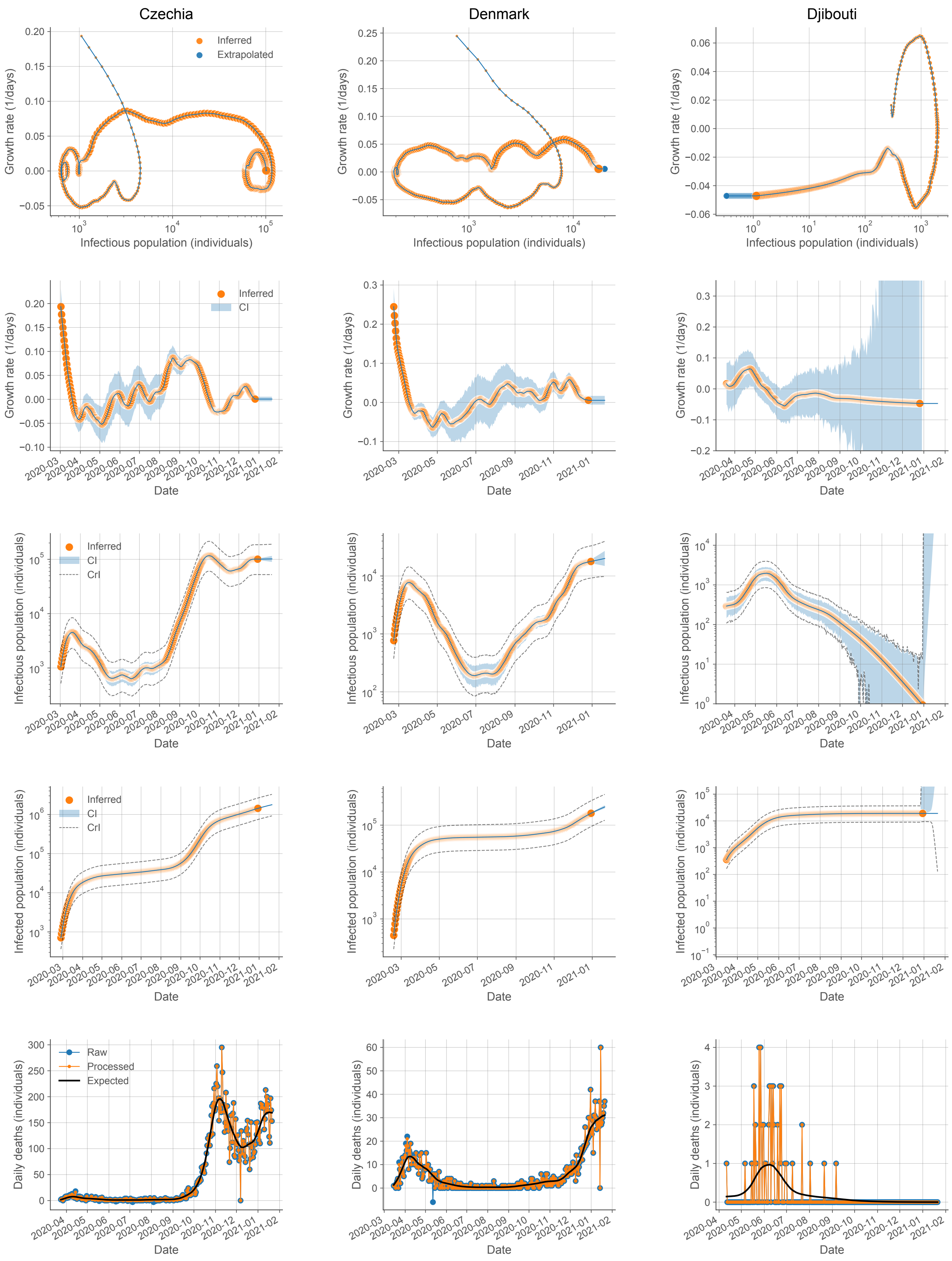
Czechia
Denmark
Djibouti
Inferred
Extrapolated
Growth rate (1/days)
Infectious population (individuals)
CI
Date
CrI
Infected population (individuals)
Raw
Processed
Expected
Daily deaths (individuals)

Figure S1.14

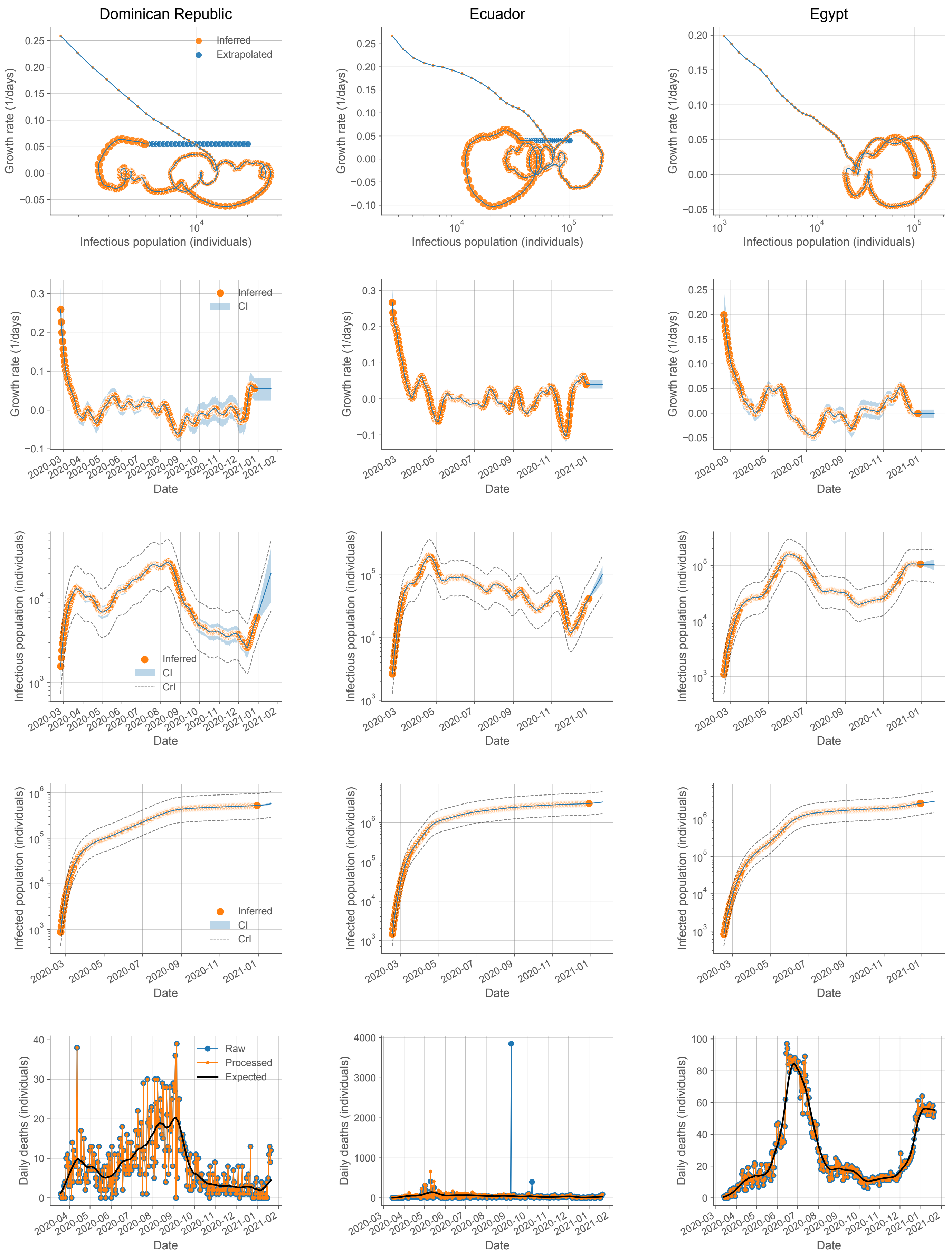
Dominican Republic
Ecuador
Egypt
Growth rate (1/days)
Infectious population (individuals)
Inferred
Extrapolated
CI
CrI
Date
Infected population (individuals)
Daily deaths (individuals)
Raw
Processed
Expected

Figure S1.15

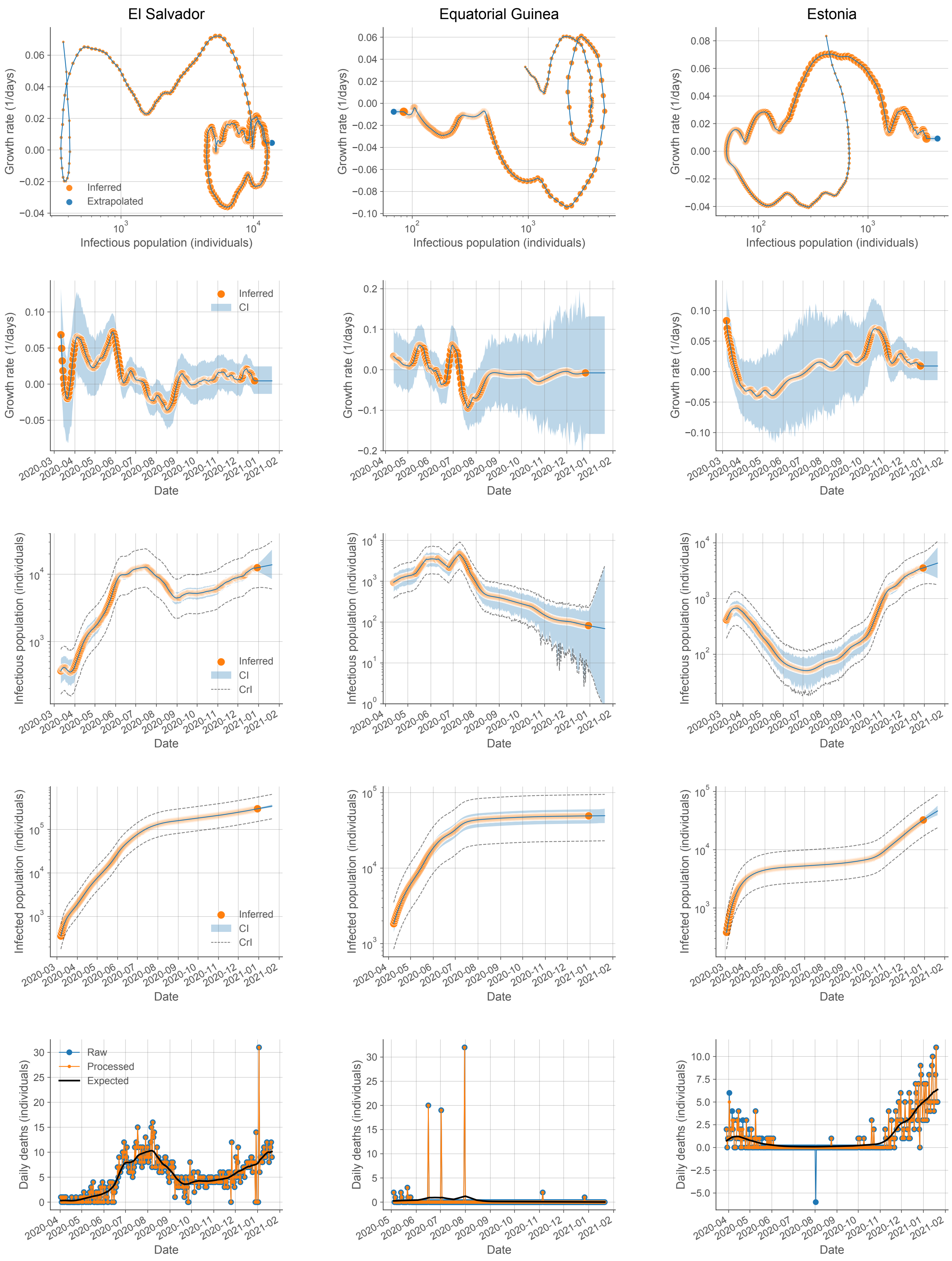

El Salvador
Equatorial Guinea
Estonia
Growth rate (1/days)
Infectious population (individuals)
Inferred
Extrapolated
CI
CrI
Date
Infected population (individuals)
Daily deaths (individuals)
Raw
Processed
Expected

Figure S1.16

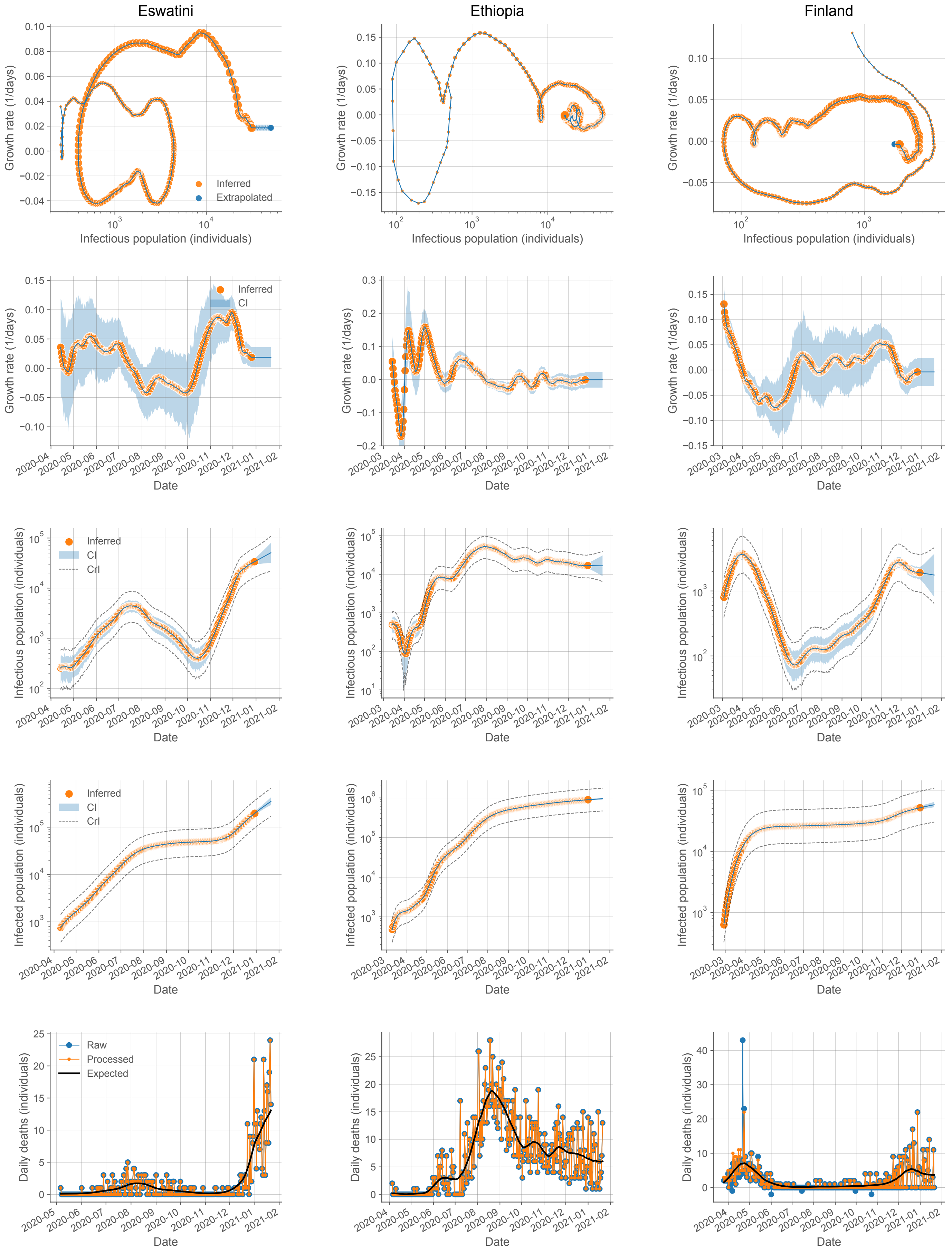
Eswatini
Ethiopia
Finland
Growth rate (1/days)
Infectious population (individuals)
Inferred
Extrapolated
CI
CrI
Date
Infected population (individuals)
Daily deaths (individuals)
Raw
Processed
Expected

Figure S1.17

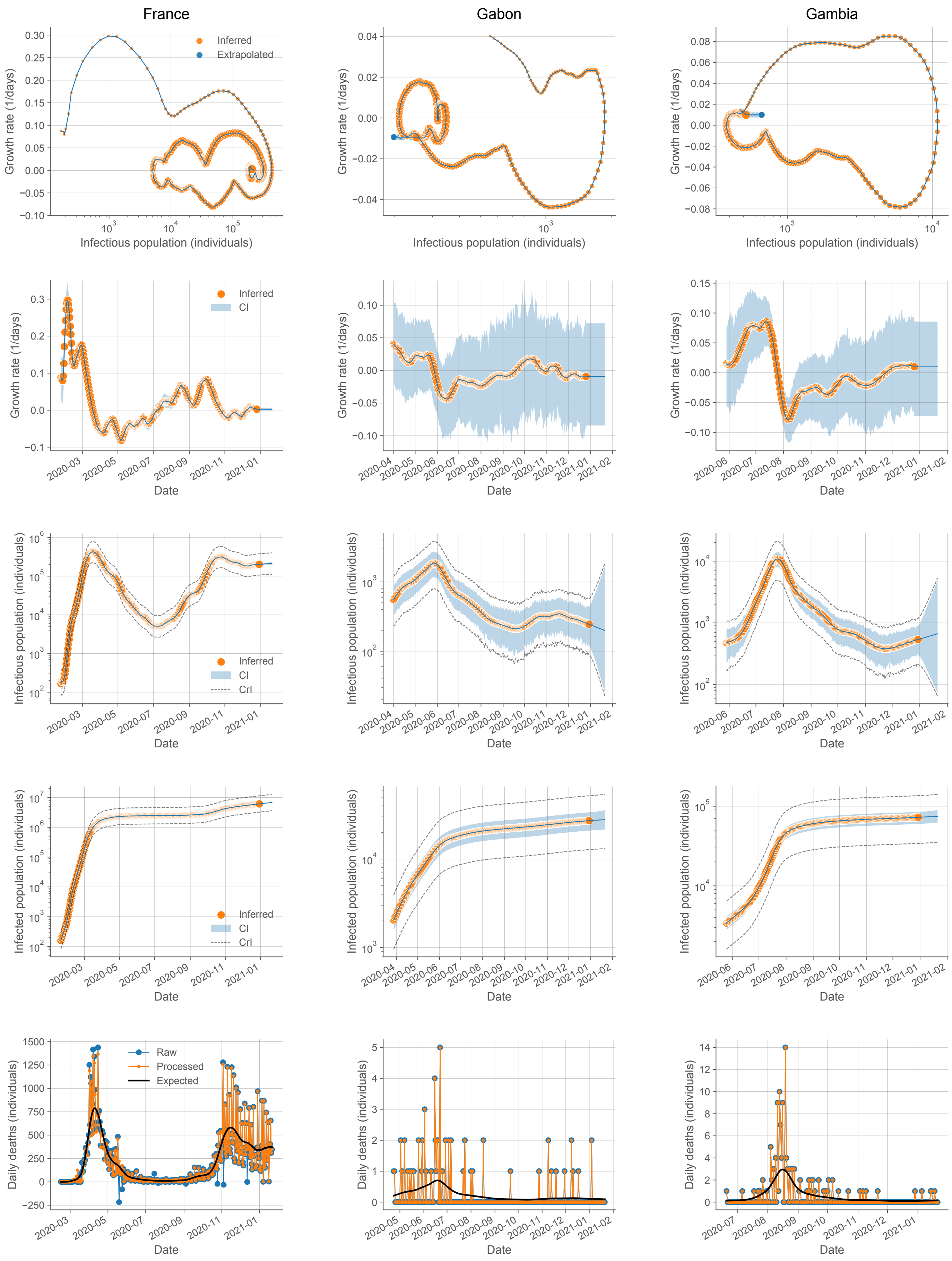
France
Gabon
Gambia
Growth rate (1/days)
Infectious population (individuals)
Inferred
Extrapolated
CI
CrI
Date
Infected population (individuals)
Daily deaths (individuals)
Raw
Processed
Expected

Figure S1.18

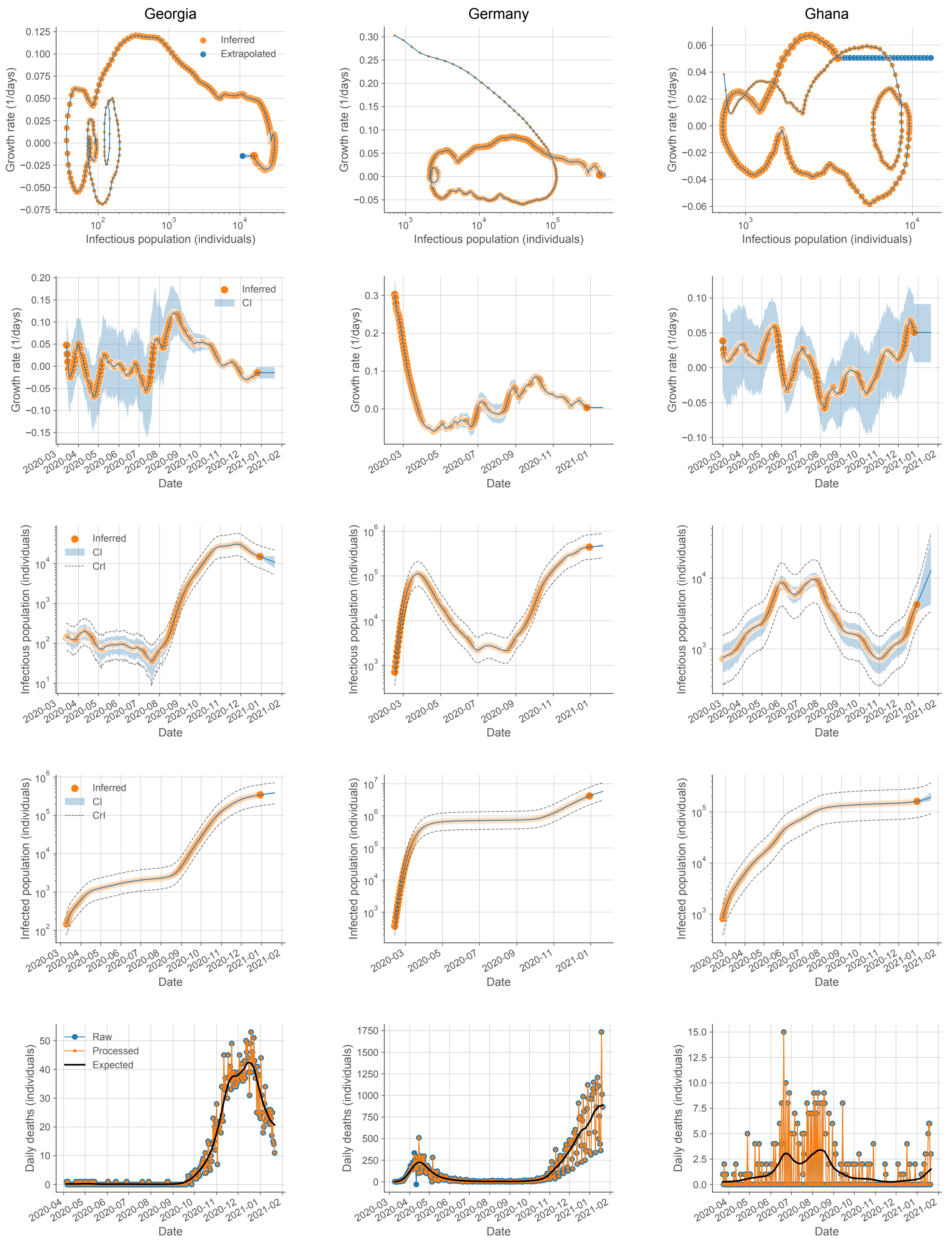

Georgia
Germany
Ghana
Inferred
Extrapolated
CI
CrI
Raw
Processed
Expected
Growth rate (1/days)
Infectious population (individuals)
Infected population (individuals)
Daily deaths (individuals)
Date

Figure S1.19

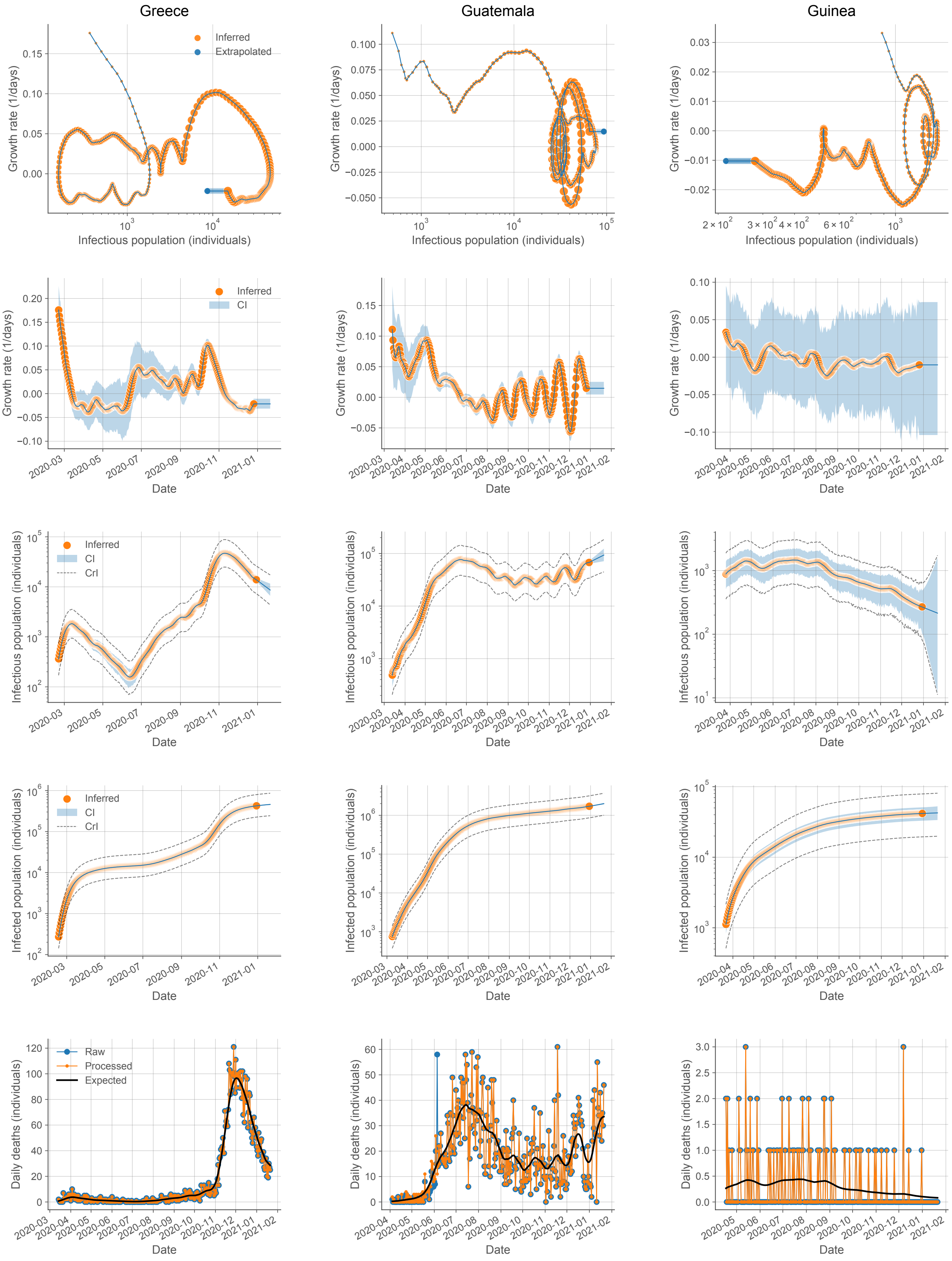
Greece
Guatemala
Guinea
Inferred
Extrapolated
CI
CrI
Raw
Processed
Expected
Growth rate (1/days)
Infectious population (individuals)
Infected population (individuals)
Daily deaths (individuals)
Date

Figure S1.20

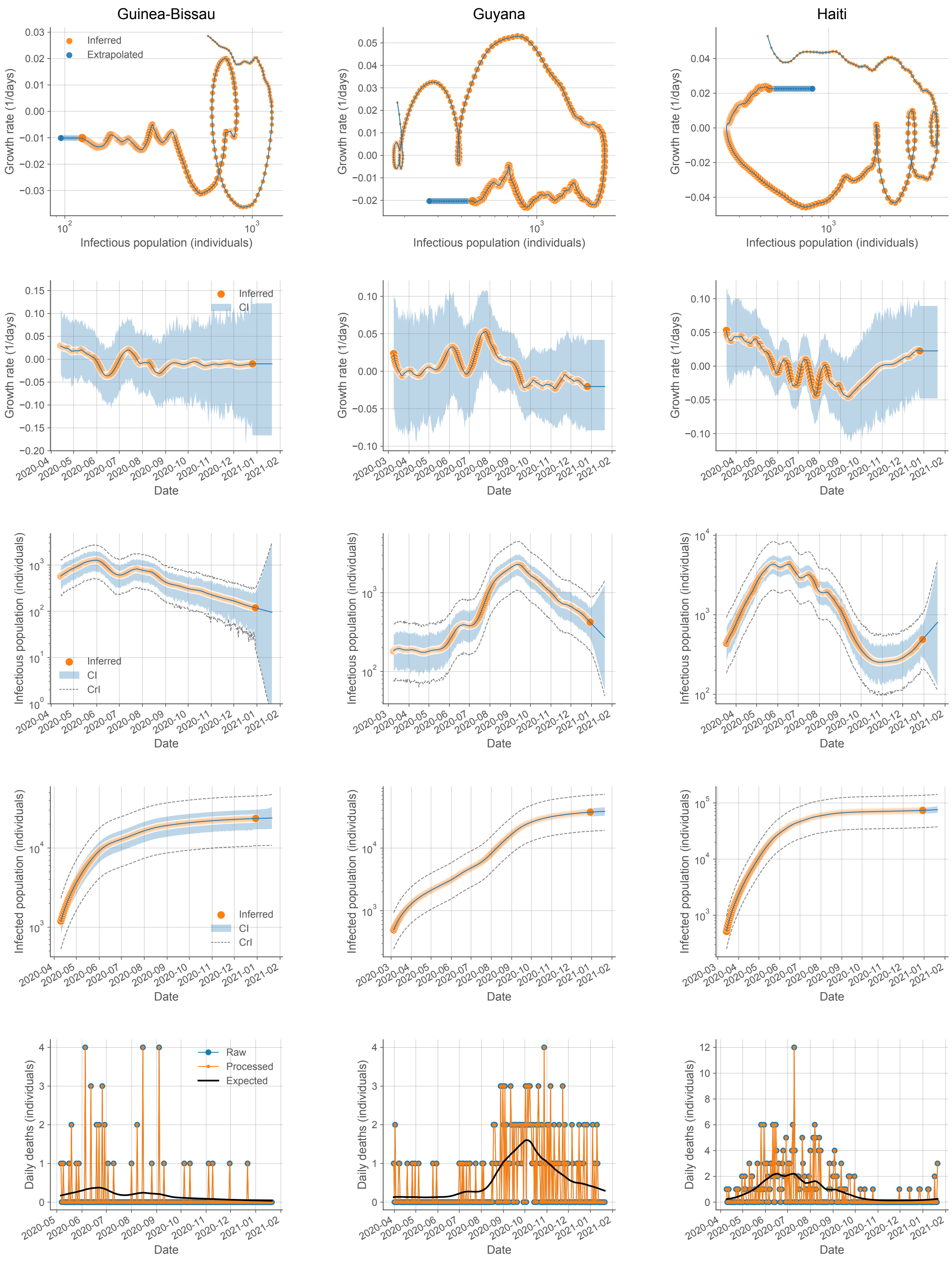

Guinea-Bissau
Guyana
Haiti
Inferred
Extrapolated
CI
CrI
Raw
Processed
Expected
Growth rate (1/days)
Infectious population (individuals)
Infected population (individuals)
Daily deaths (individuals)
Date

Figure S1.21

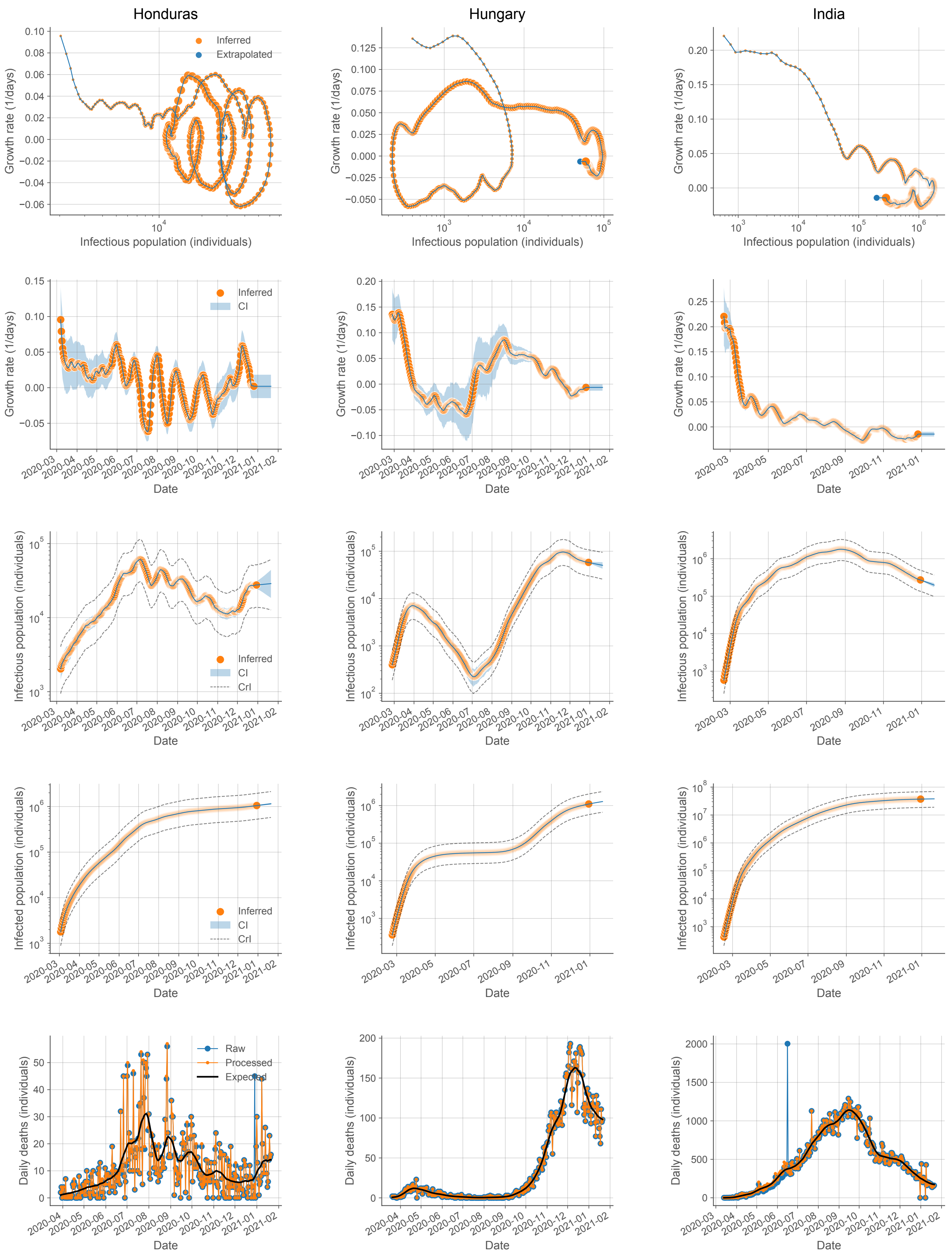
Honduras
Hungary
India
Growth rate (1/days)
Infectious population (individuals)
Inferred
Extrapolated
CI
CrI
Date
Infected population (individuals)
Daily deaths (individuals)
Raw
Processed
Expected

Figure S1.22

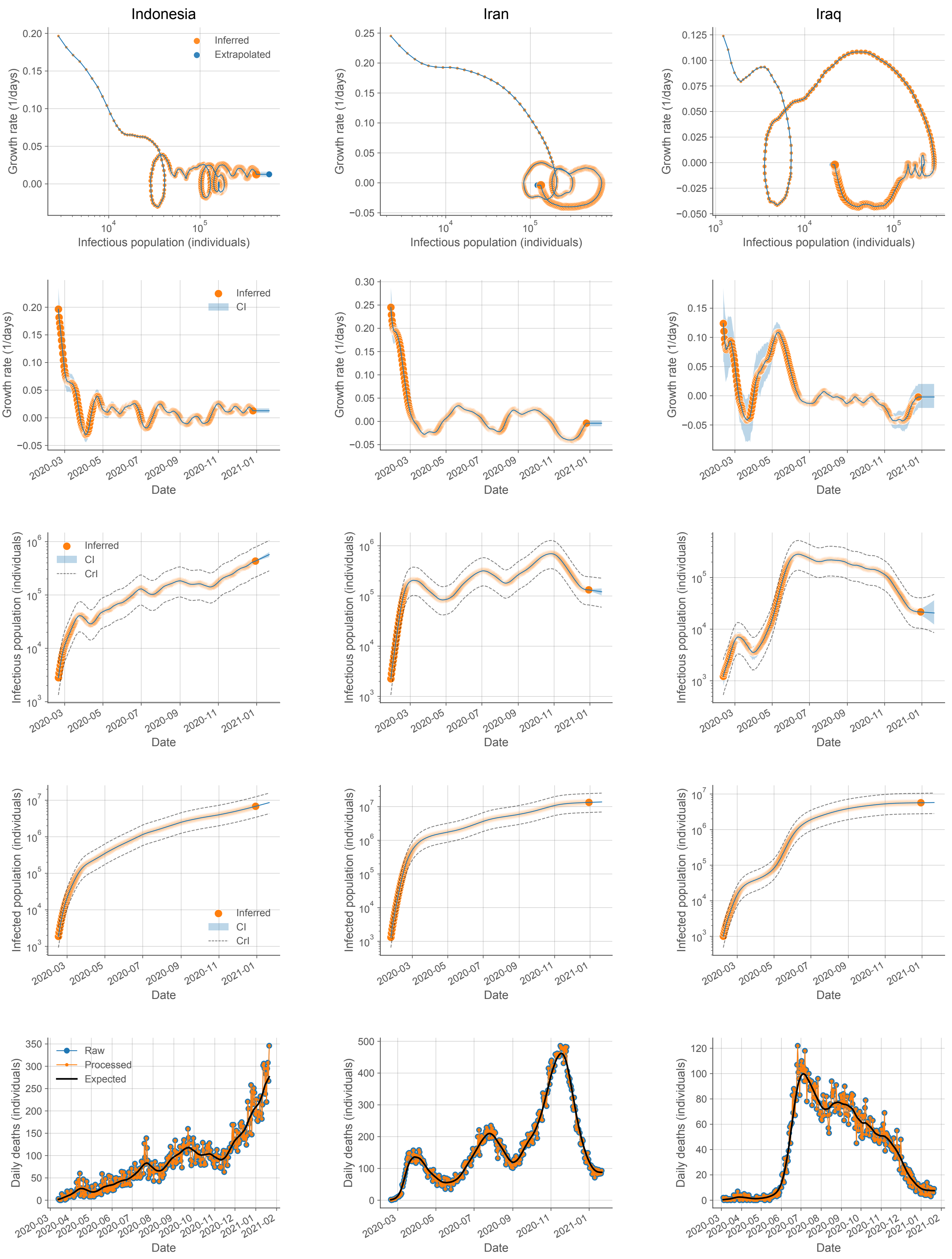
Indonesia
Iran
Iraq
Inferred
Extrapolated
Growth rate (1/days)
Infectious population (individuals)
CI
CrI
Date
Infected population (individuals)
Raw
Processed
Expected
Daily deaths (individuals)

Figure S1.23

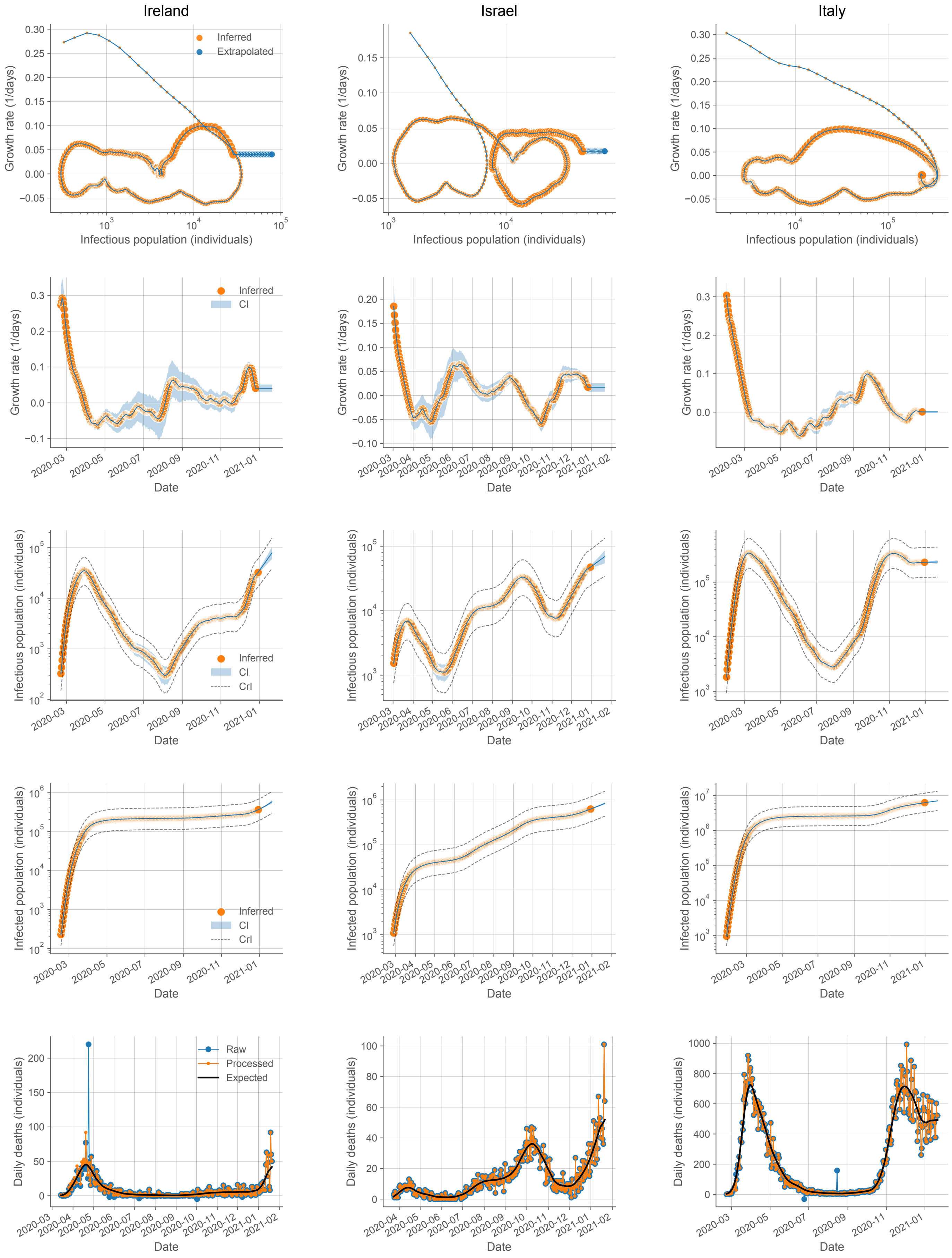
Ireland
Israel
Italy
Inferred
Extrapolated
Growth rate (1/days)
Infectious population (individuals)
CI
CrI
Date
Infected population (individuals)
Daily deaths (individuals)
Raw
Processed
Expected

Figure S1.24

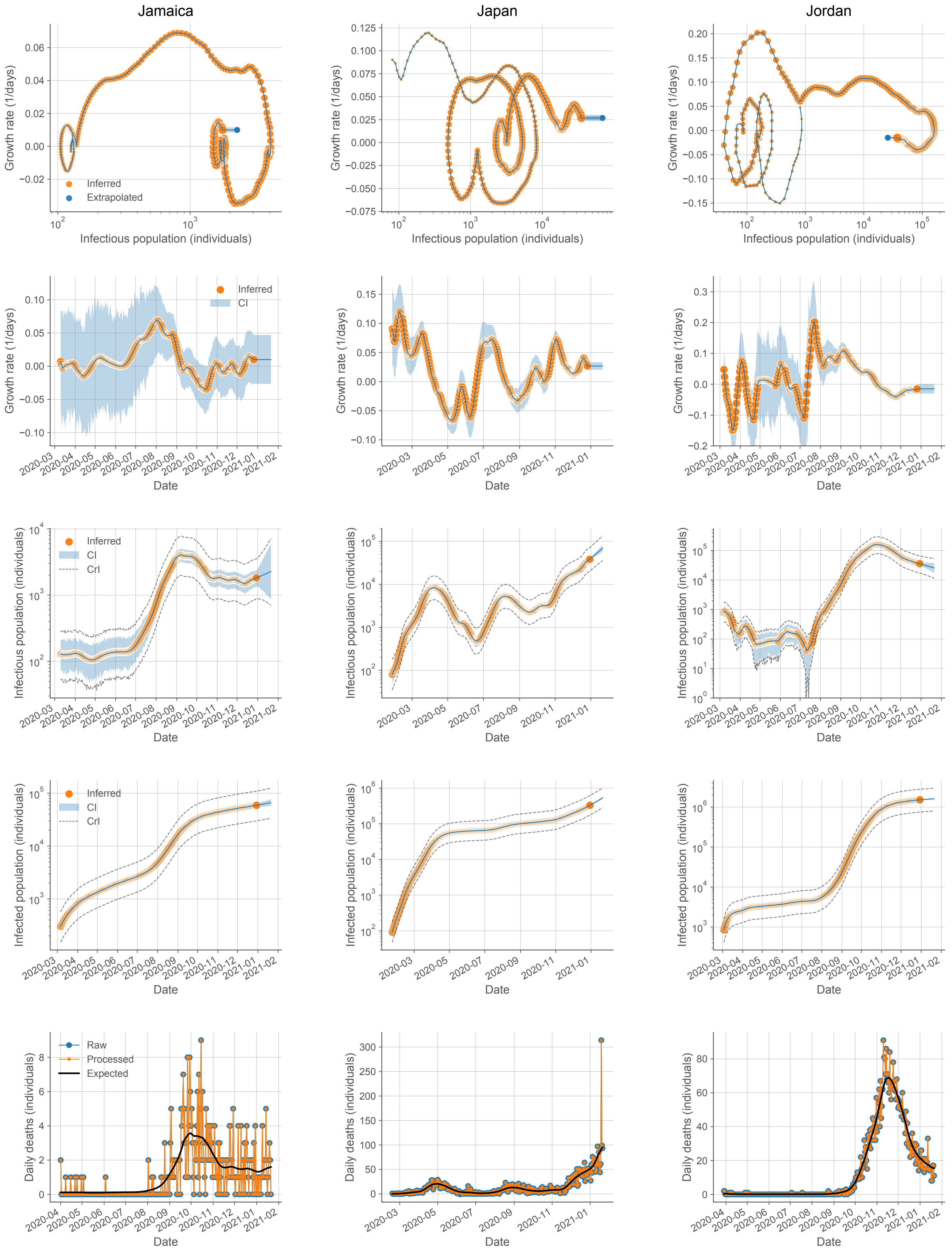

Jamaica
Japan
Jordan
Growth rate (1/days)
Infectious population (individuals)
Inferred
Extrapolated
CI
CrI
Date
Infected population (individuals)
Daily deaths (individuals)
Raw
Processed
Expected

Figure S1.25

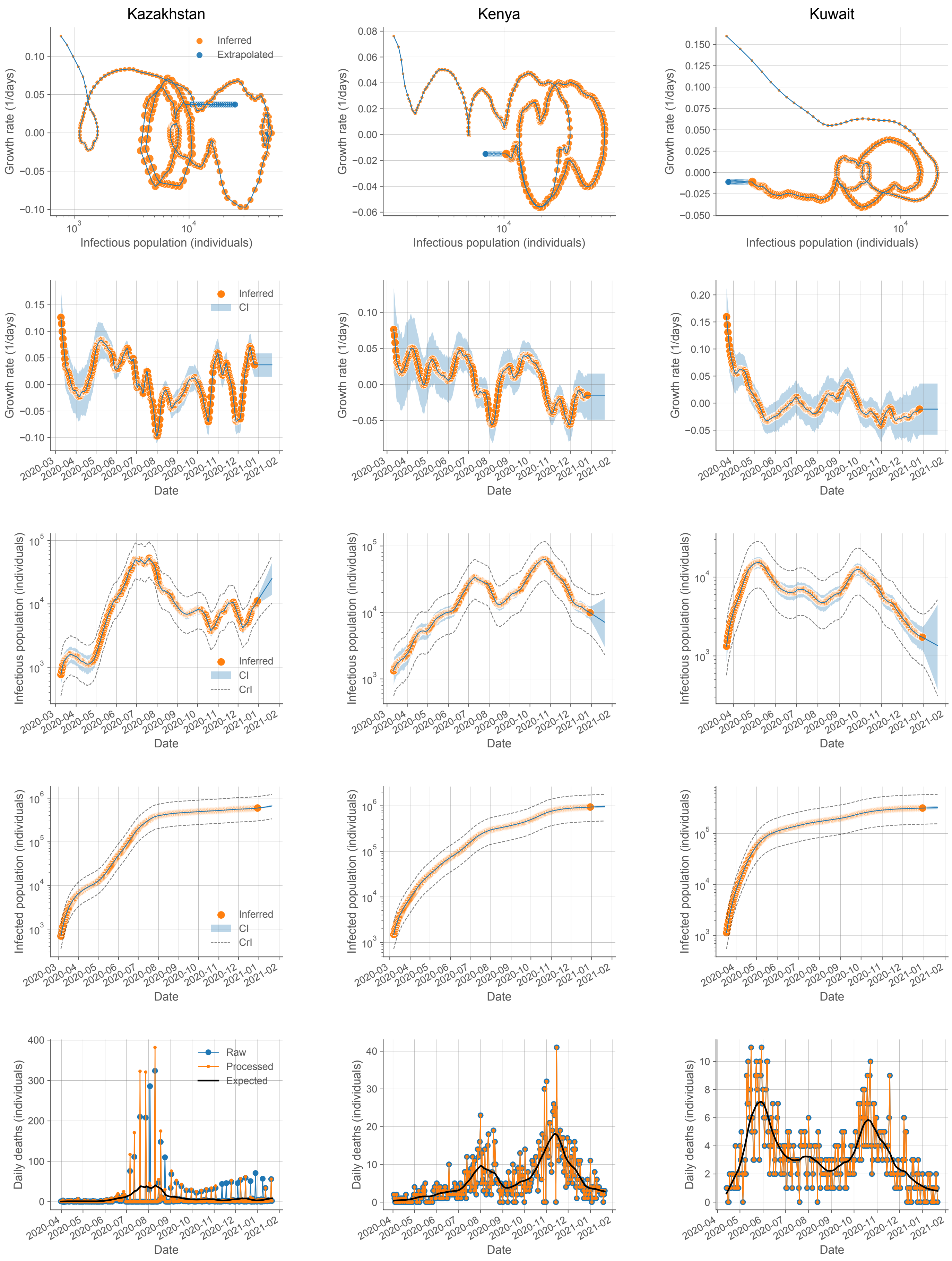
Kazakhstan
Kenya
Kuwait
Inferred
Extrapolated
Growth rate (1/days)
Infectious population (individuals)
CI
CrI
Date
Infected population (individuals)
Daily deaths (individuals)
Raw
Processed
Expected

Figure S1.26

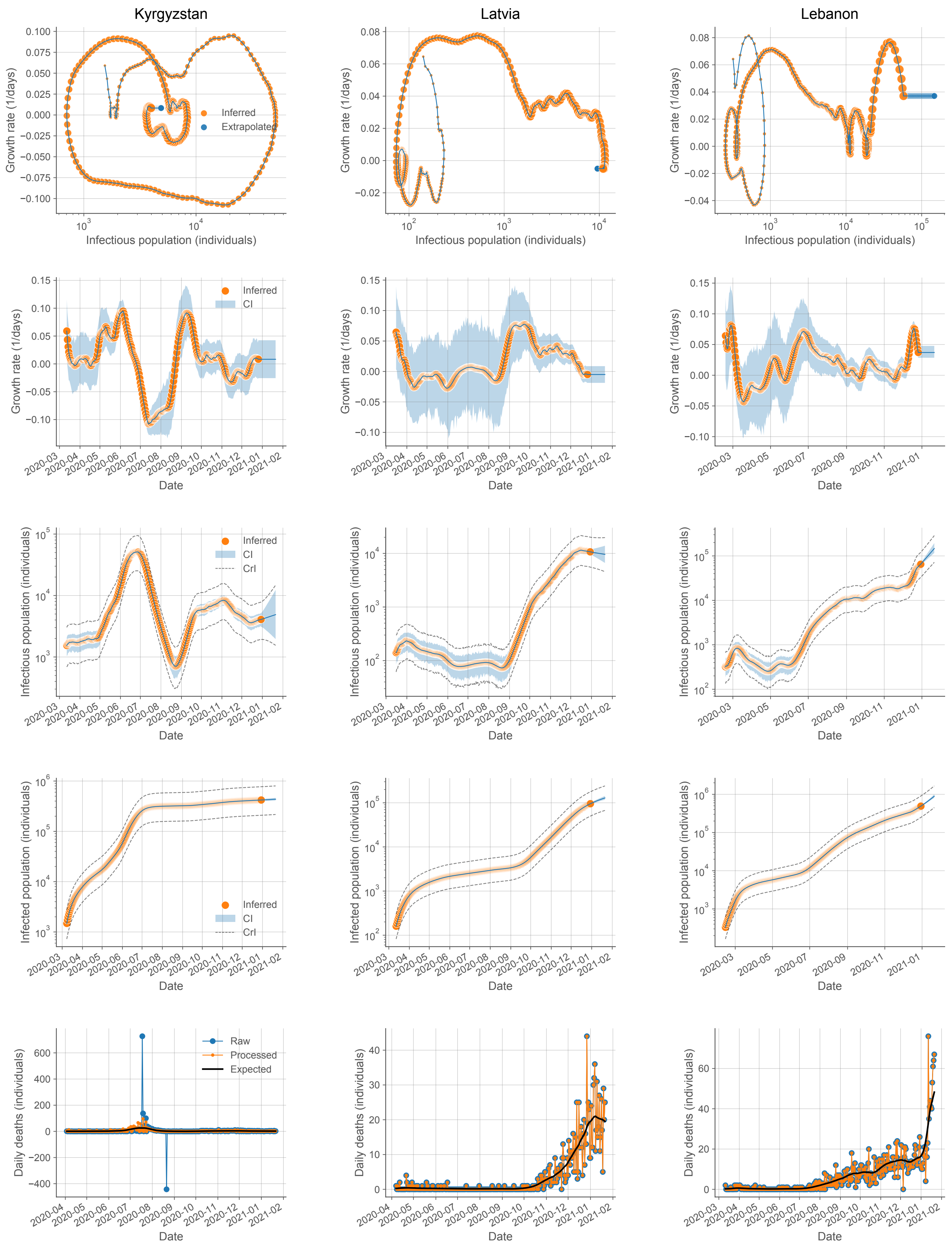

Kyrgyzstan
Latvia
Lebanon
Growth rate (1/days)
Infectious population (individuals)
Inferred
Extrapolated
CI
CrI
Date
Infected population (individuals)
Daily deaths (individuals)
Raw
Processed
Expected

Figure S1.27

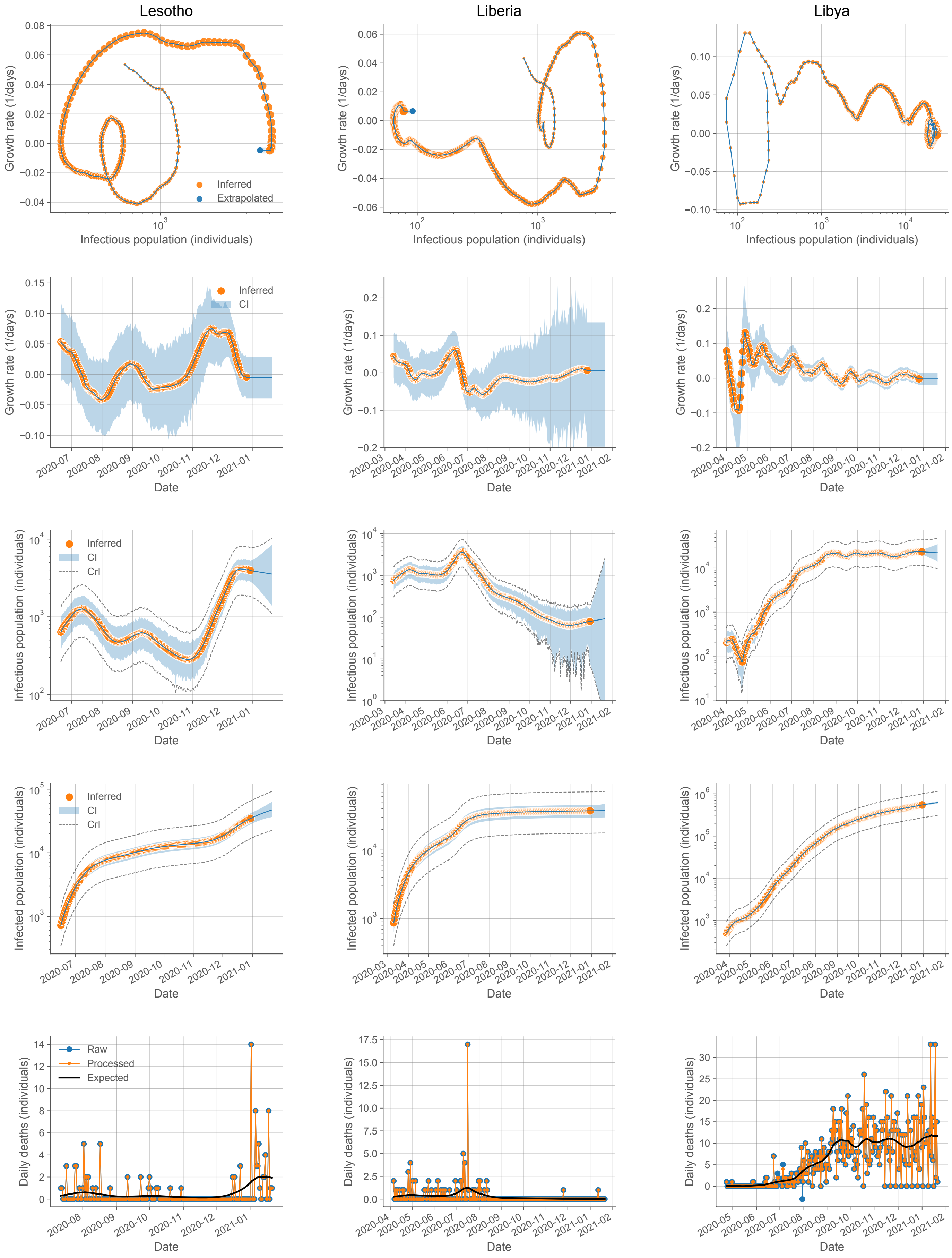

Lesotho
Liberia
Libya
Growth rate (1/days)
Infectious population (individuals)
Inferred
Extrapolated
CI
CrI
Date
Infected population (individuals)
Daily deaths (individuals)
Raw
Processed
Expected

Figure S1.28

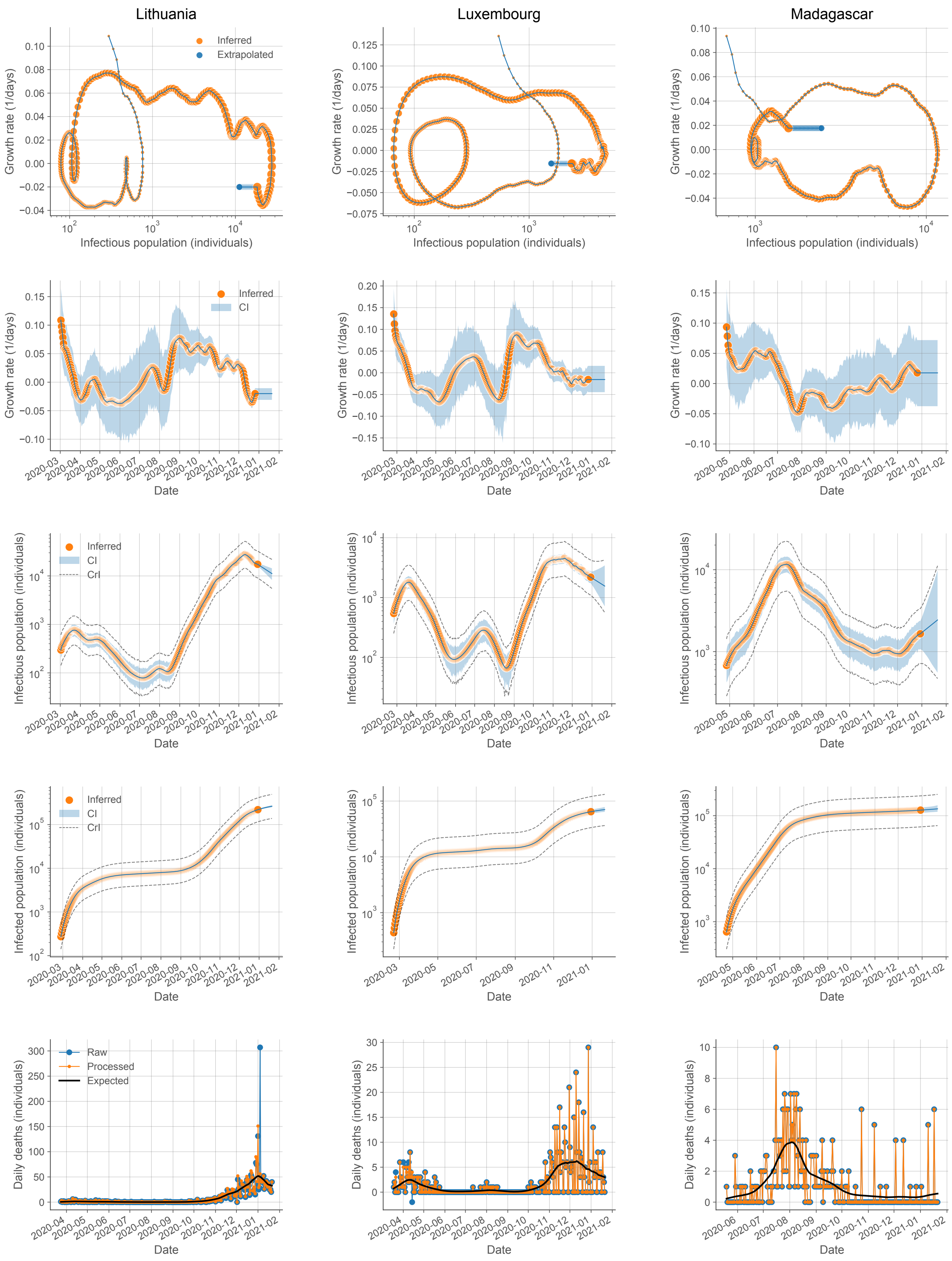
Lithuania
Luxembourg
Madagascar
Inferred
Extrapolated
CI
CrI
Raw
Processed
Expected
Growth rate (1/days)
Infectious population (individuals)
Infected population (individuals)
Daily deaths (individuals)
Date

Figure S1.29

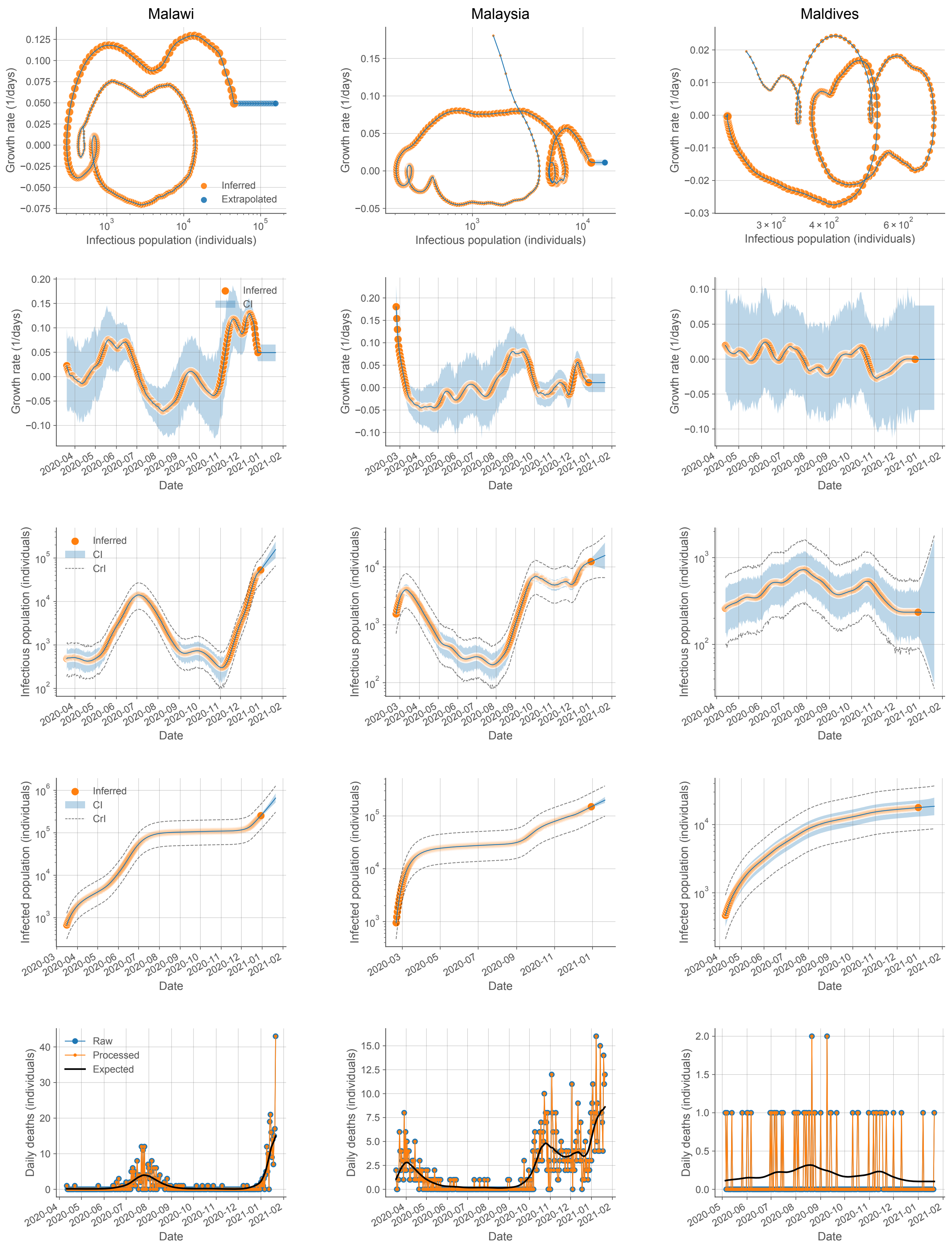

Malawi
Malaysia
Maldives
Growth rate (1/days)
Infectious population (individuals)
Inferred
Extrapolated
CI
CrI
Date
Infected population (individuals)
Daily deaths (individuals)
Raw
Processed
Expected

Figure S1.30

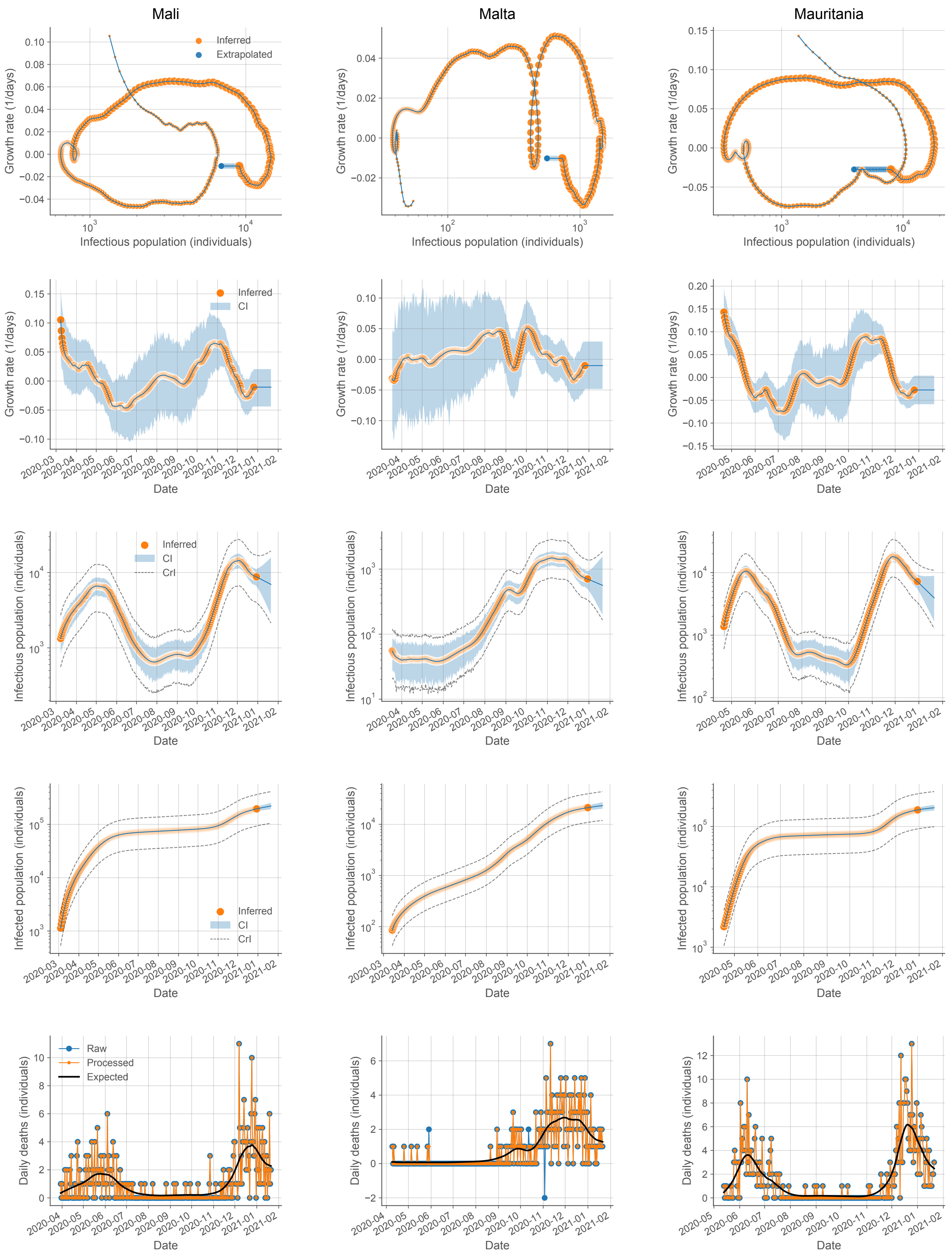

Mali
Malta
Mauritania
Inferred
Extrapolated
CI
CrI
Raw
Processed
Expected
Growth rate (1/days)
Infectious population (individuals)
Infected population (individuals)
Daily deaths (individuals)
Date

Figure S1.31

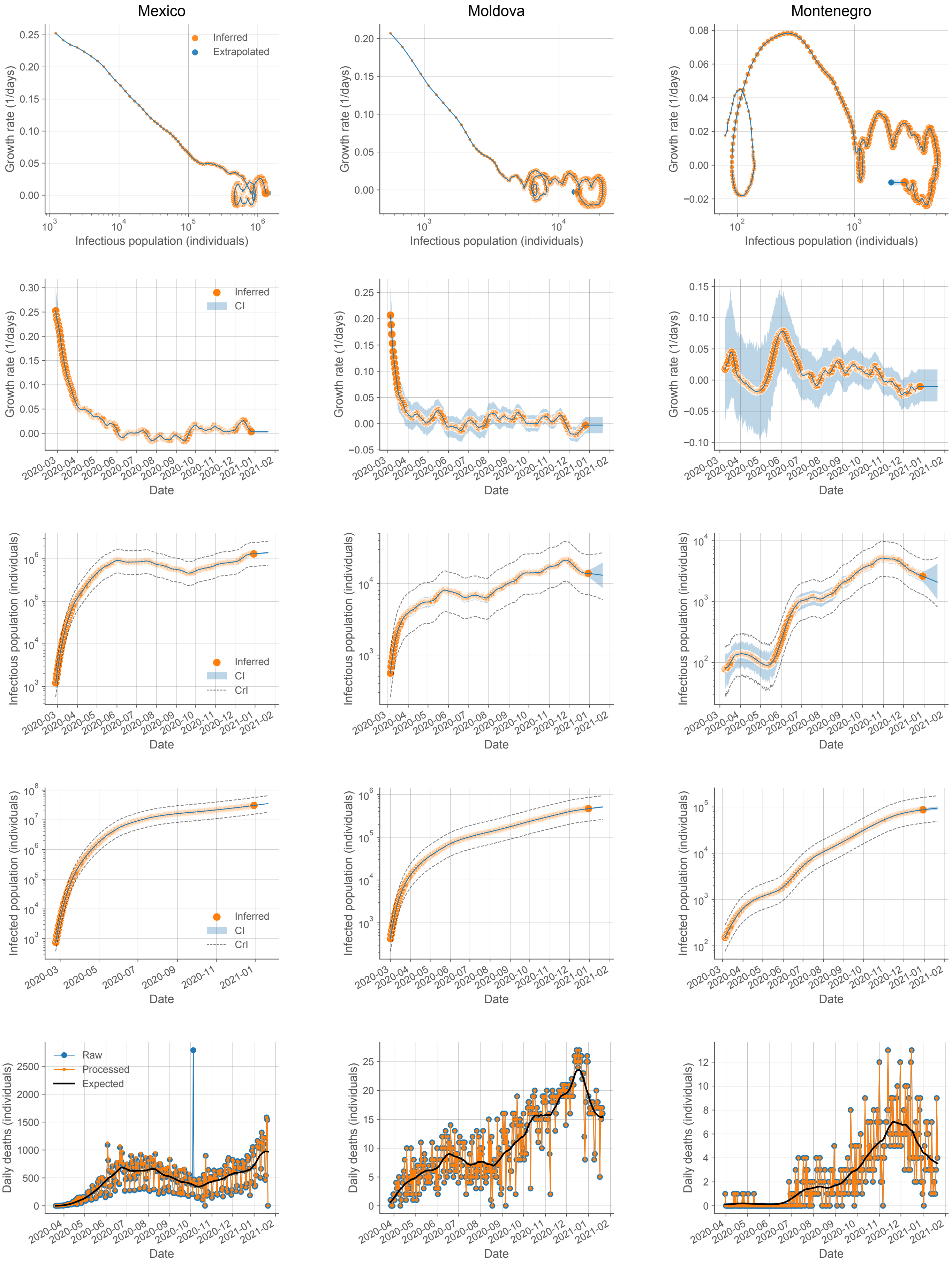
Mexico
Moldova
Montenegro
Growth rate (1/days)
Infectious population (individuals)
Inferred
Extrapolated
CI
CrI
Date
Infected population (individuals)
Daily deaths (individuals)
Raw
Processed
Expected

Figure S1.32

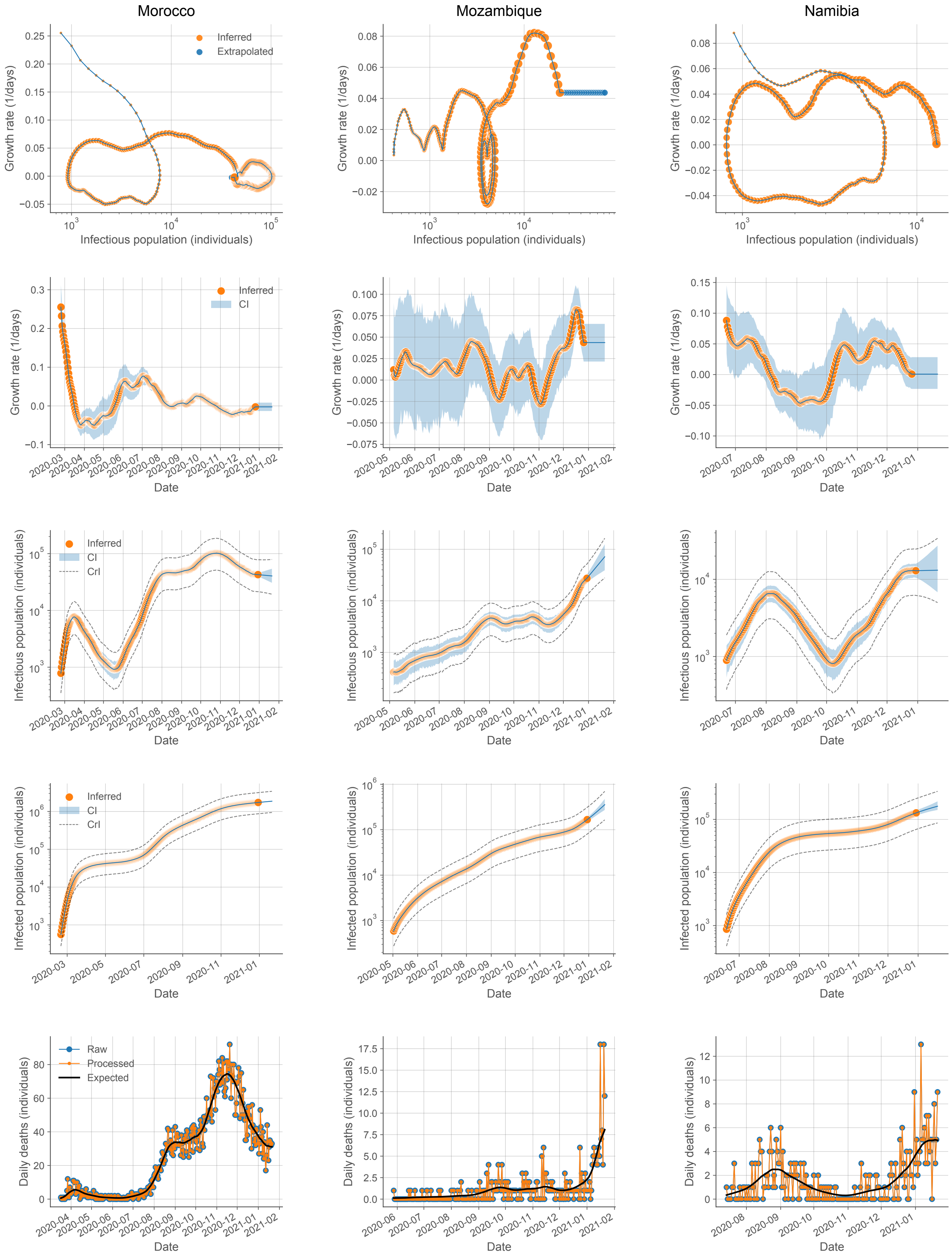

Morocco
Mozambique
Namibia
Growth rate (1/days)
Infectious population (individuals)
Inferred
Extrapolated
CI
CrI
Date
Infected population (individuals)
Daily deaths (individuals)
Raw
Processed
Expected

Figure S1.33

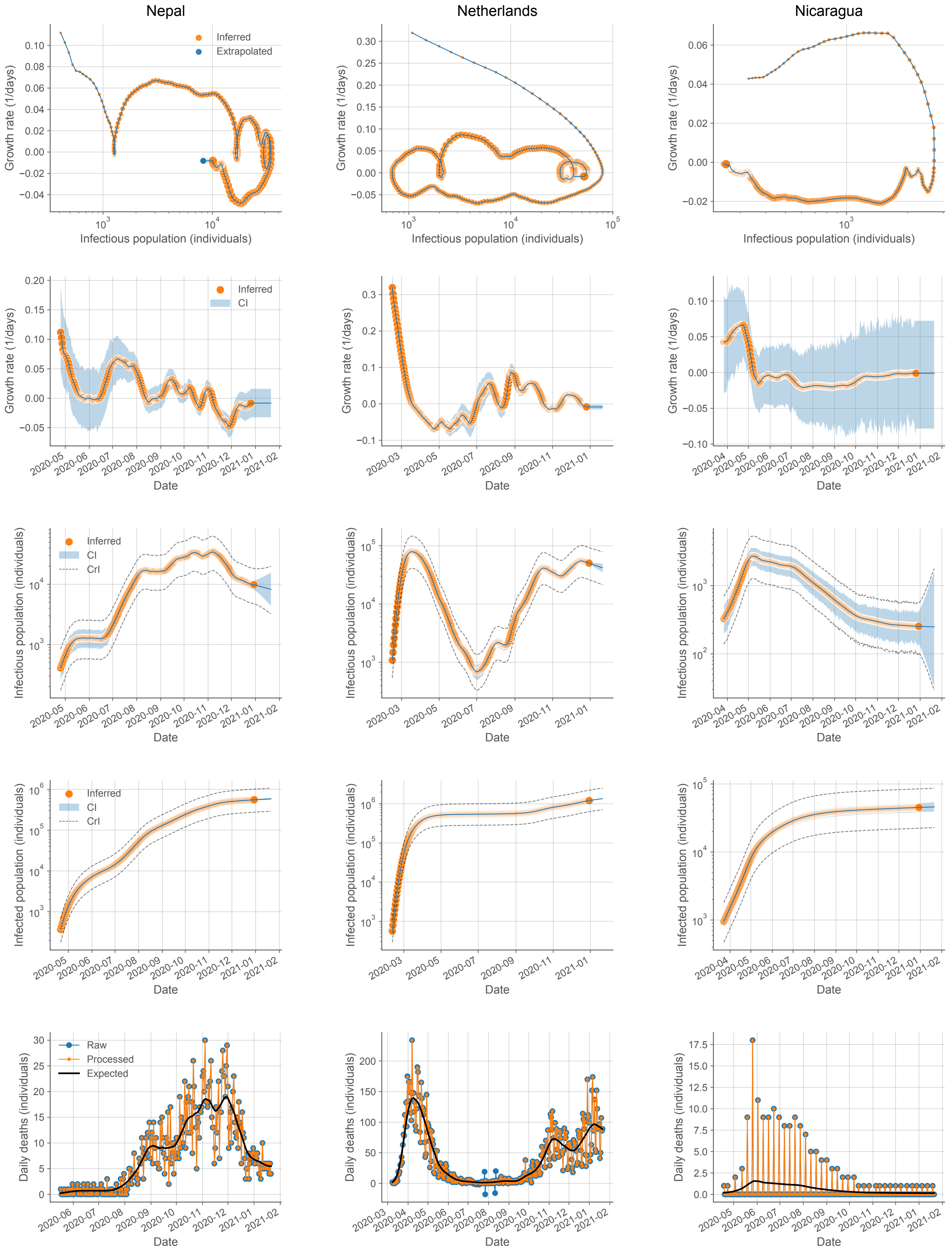

Nepal
Netherlands
Nicaragua
Growth rate (1/days)
Infectious population (individuals)
Inferred
Extrapolated
CI
CrI
Date
Infected population (individuals)
Daily deaths (individuals)
Raw
Processed
Expected

Figure S1.34

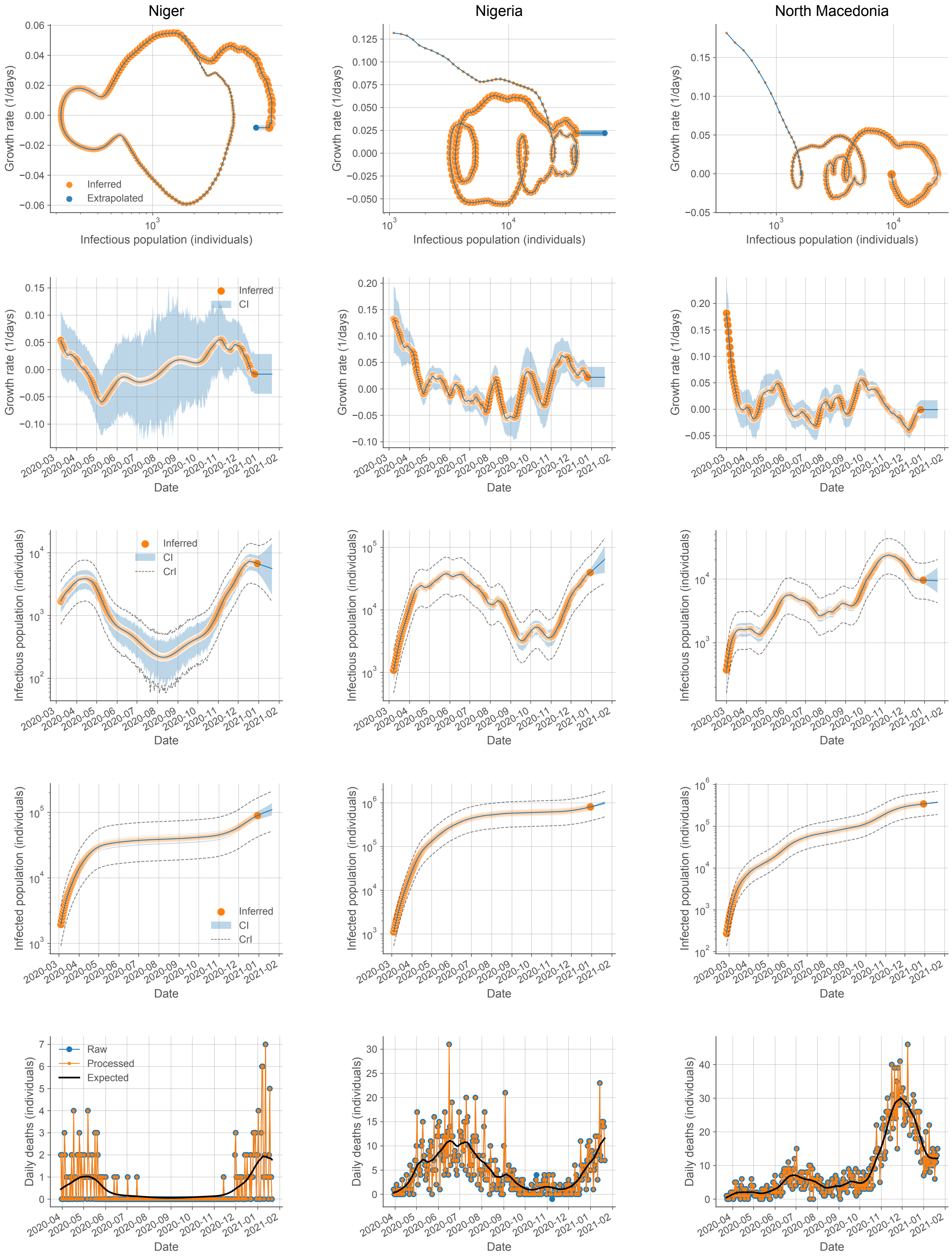
Niger
Nigeria
North Macedonia
Growth rate (1/days)
Infectious population (individuals)
Inferred
Extrapolated
CI
CrI
Date
Infected population (individuals)
Daily deaths (individuals)
Raw
Processed
Expected

Figure S1.35

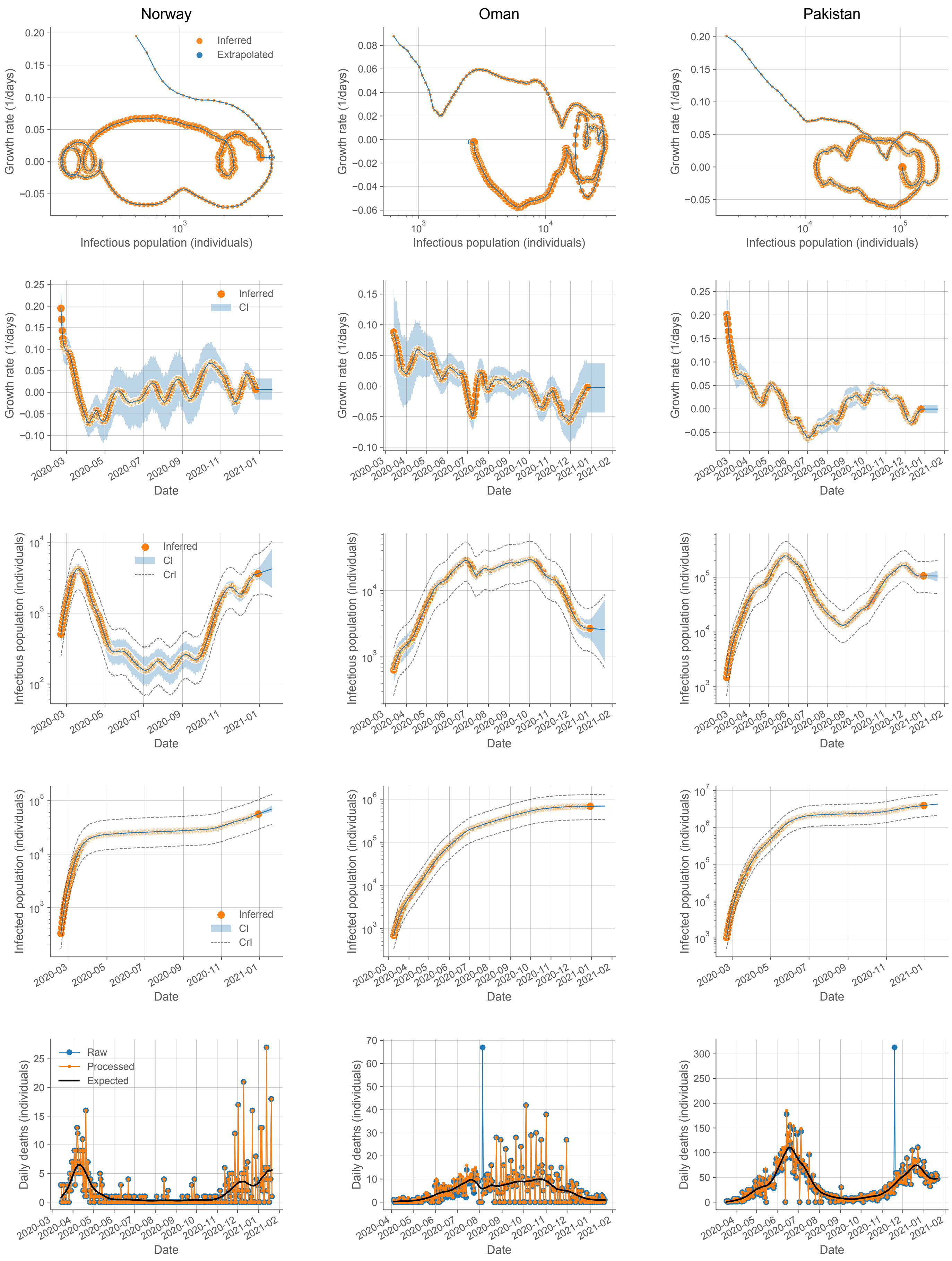

Norway
Oman
Pakistan
Growth rate (1/days)
Infectious population (individuals)
Inferred
Extrapolated
CI
CrI
Date
Infected population (individuals)
Daily deaths (individuals)
Raw
Processed
Expected

Figure S1.36

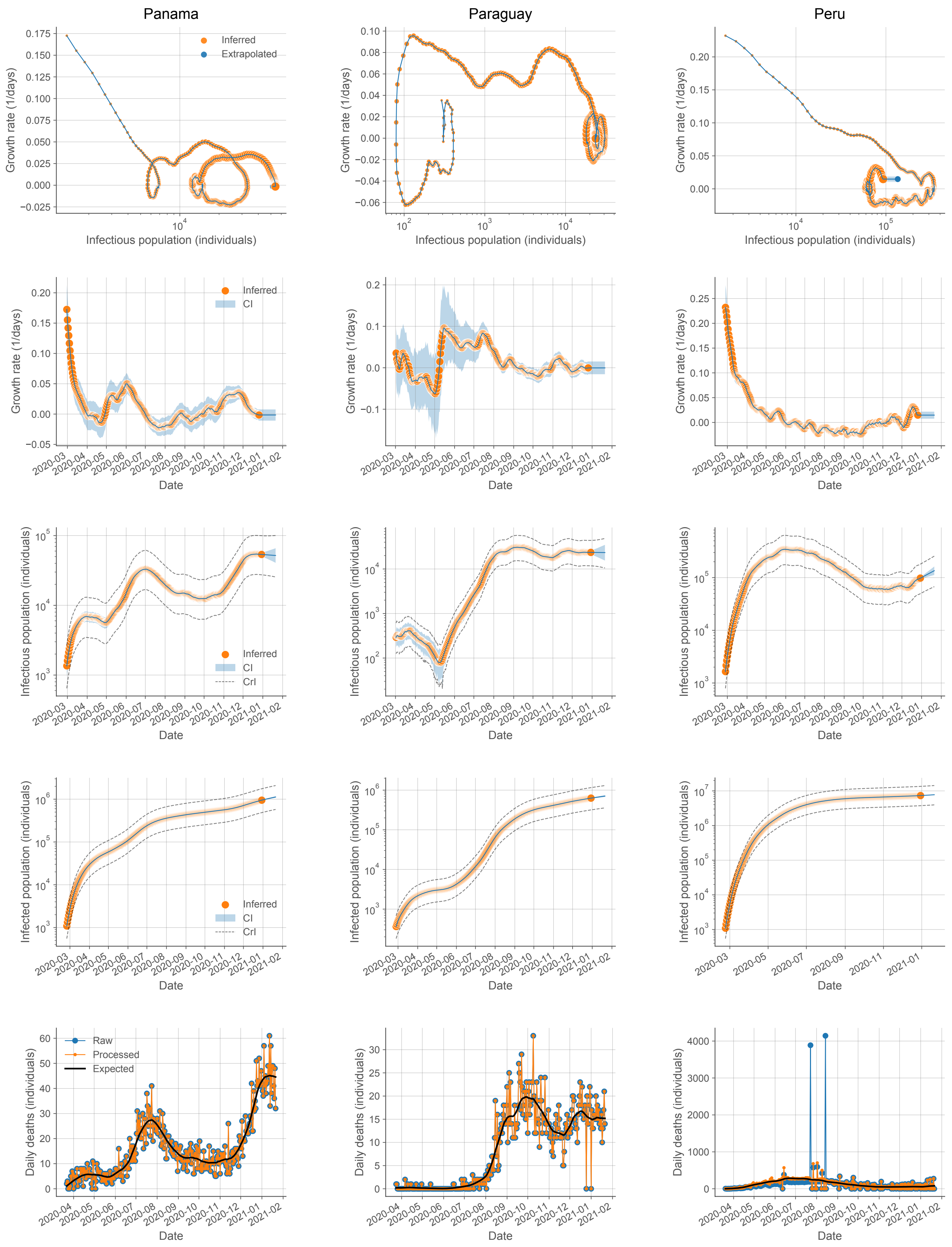

Panama
Paraguay
Peru
Growth rate (1/days)
Infectious population (individuals)
Inferred
Extrapolated
CI
CrI
Date
Infected population (individuals)
Daily deaths (individuals)
Raw
Processed
Expected

Figure S1.37

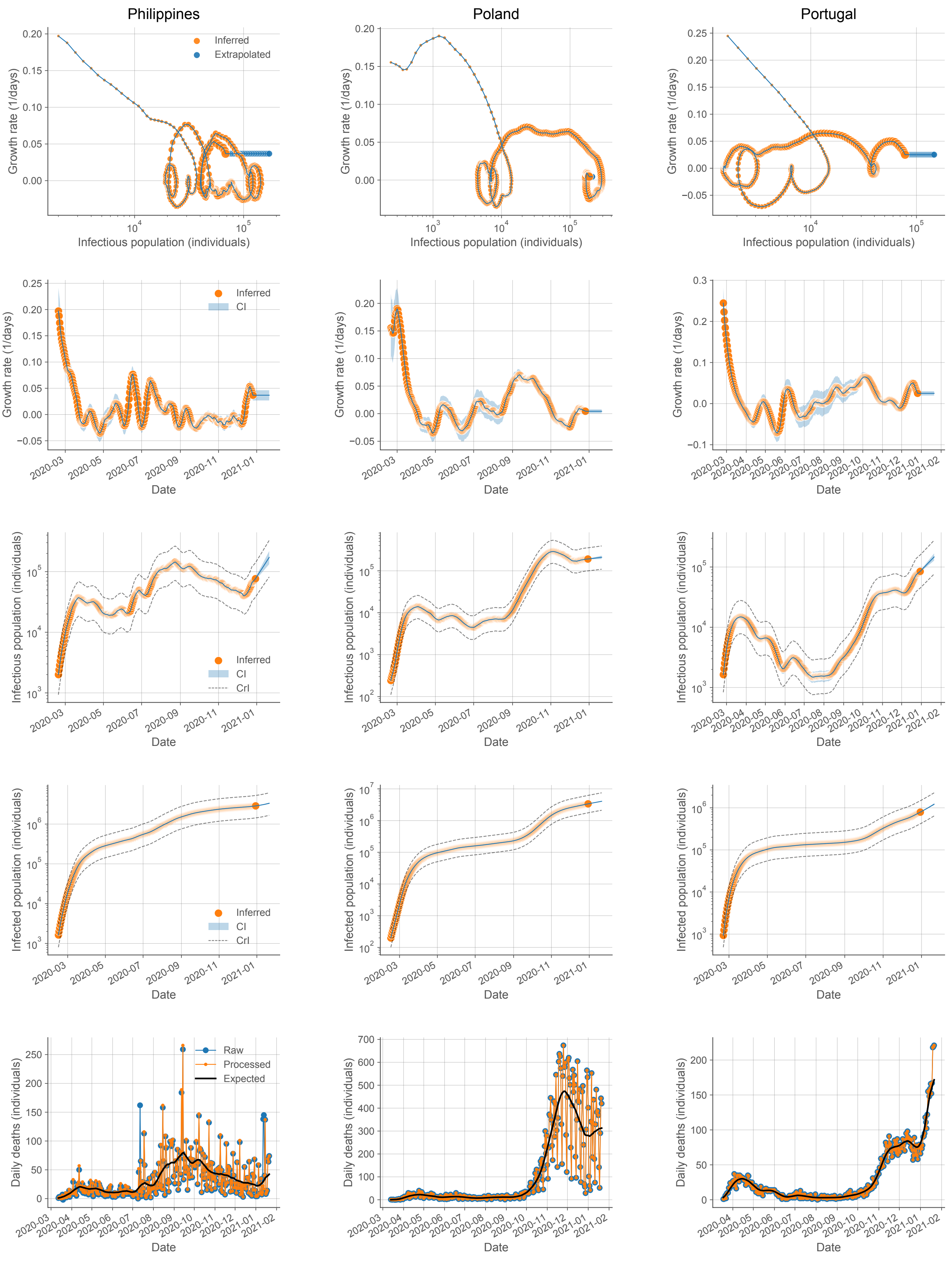

Philippines
Poland
Portugal
Inferred
Extrapolated
CI
CrI
Raw
Processed
Expected
Growth rate (1/days)
Infectious population (individuals)
Infected population (individuals)
Daily deaths (individuals)
Date

Figure S1.38

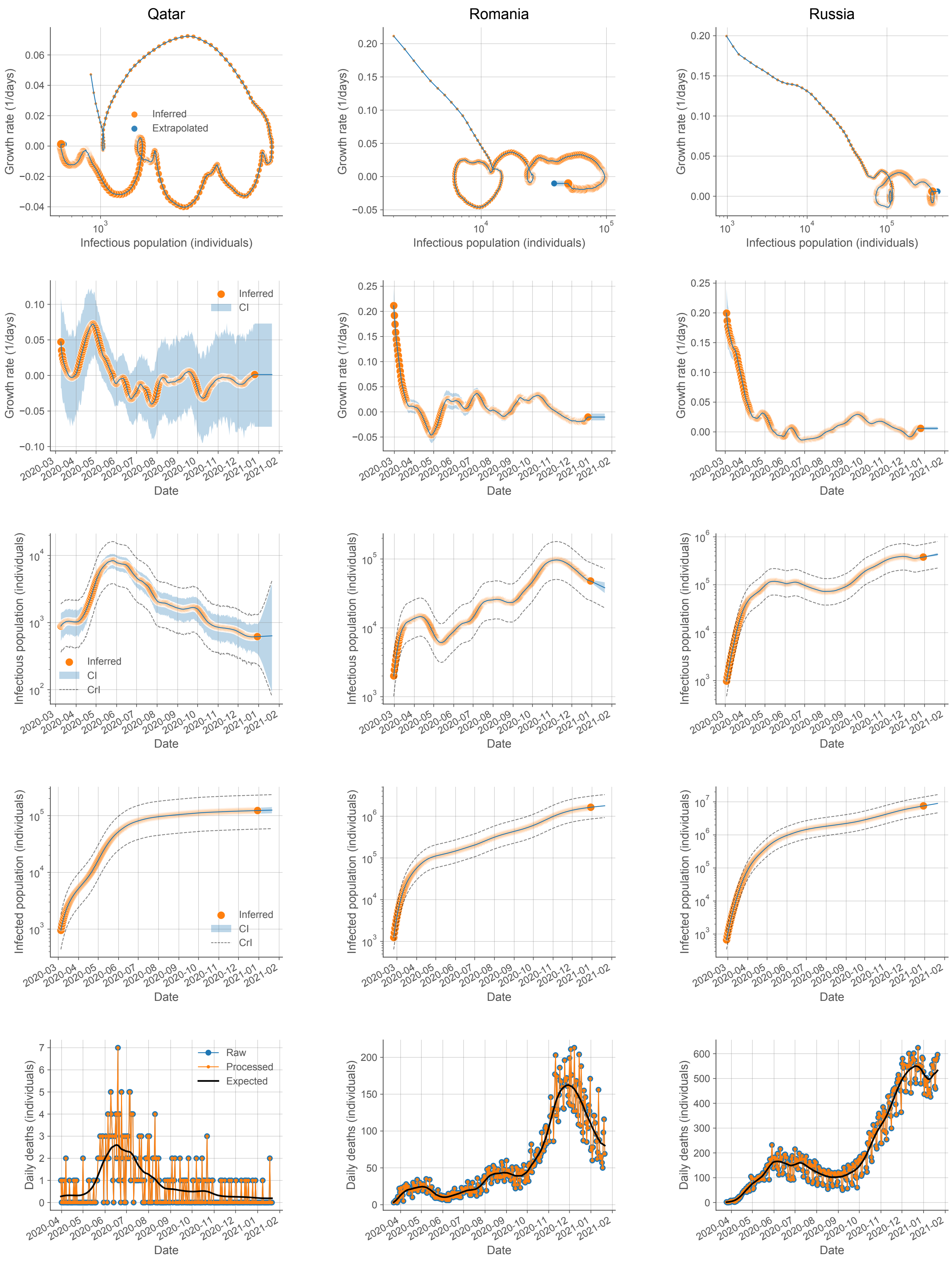

Qatar
Romania
Russia
Growth rate (1/days)
Infectious population (individuals)
Inferred
Extrapolated
CI
CrI
Date
Infected population (individuals)
Daily deaths (individuals)
Raw
Processed
Expected

Figure S1.39

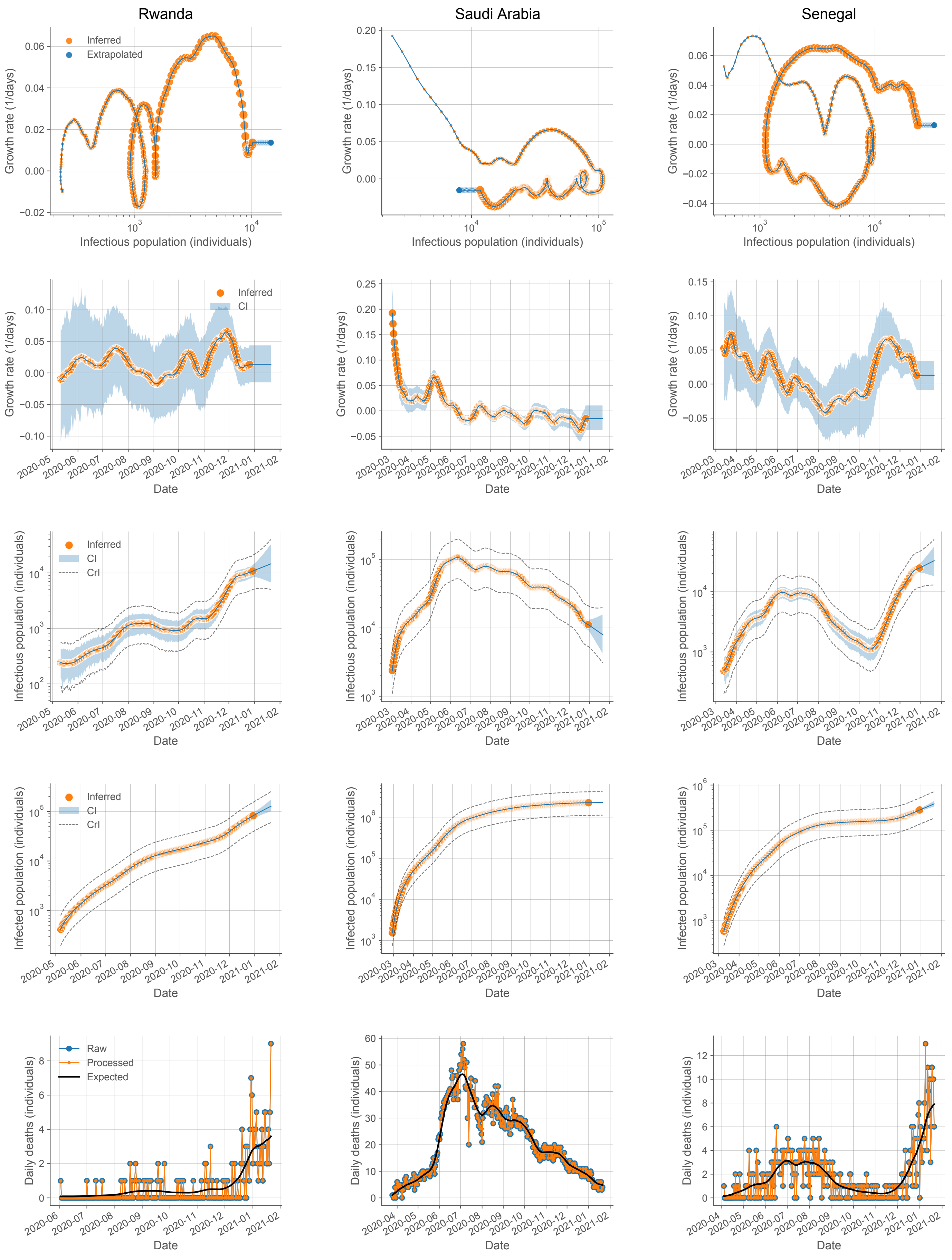

Rwanda
Saudi Arabia
Senegal
Inferred
Extrapolated
CI
CrI
Raw
Processed
Expected
Growth rate (1/days)
Infectious population (individuals)
Infected population (individuals)
Daily deaths (individuals)
Date

Figure S1.40

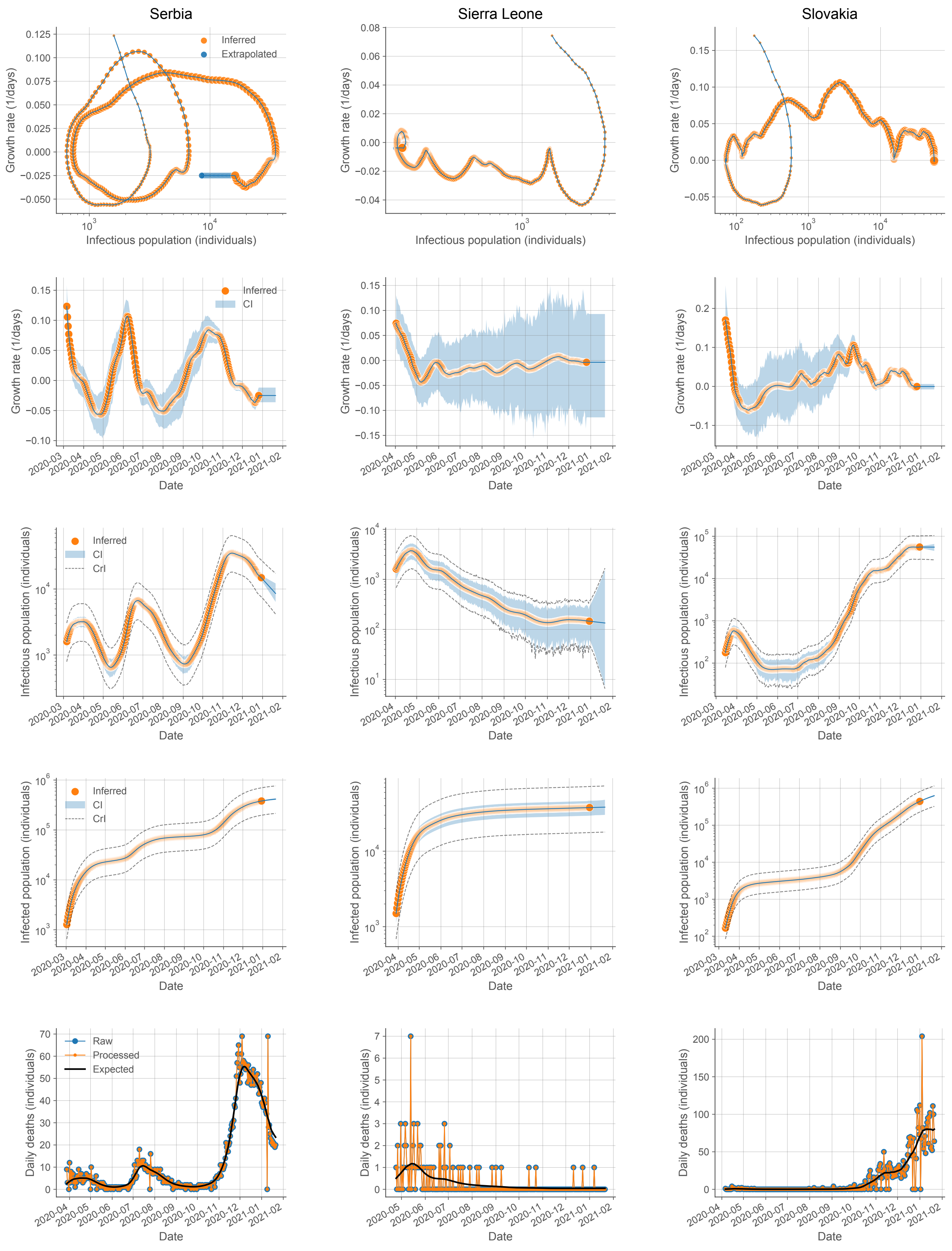

Serbia
Sierra Leone
Slovakia
Growth rate (1/days)
Infectious population (individuals)
Inferred
Extrapolated
CI
CrI
Date
Infected population (individuals)
Daily deaths (individuals)
Raw
Processed
Expected

Figure S1.41

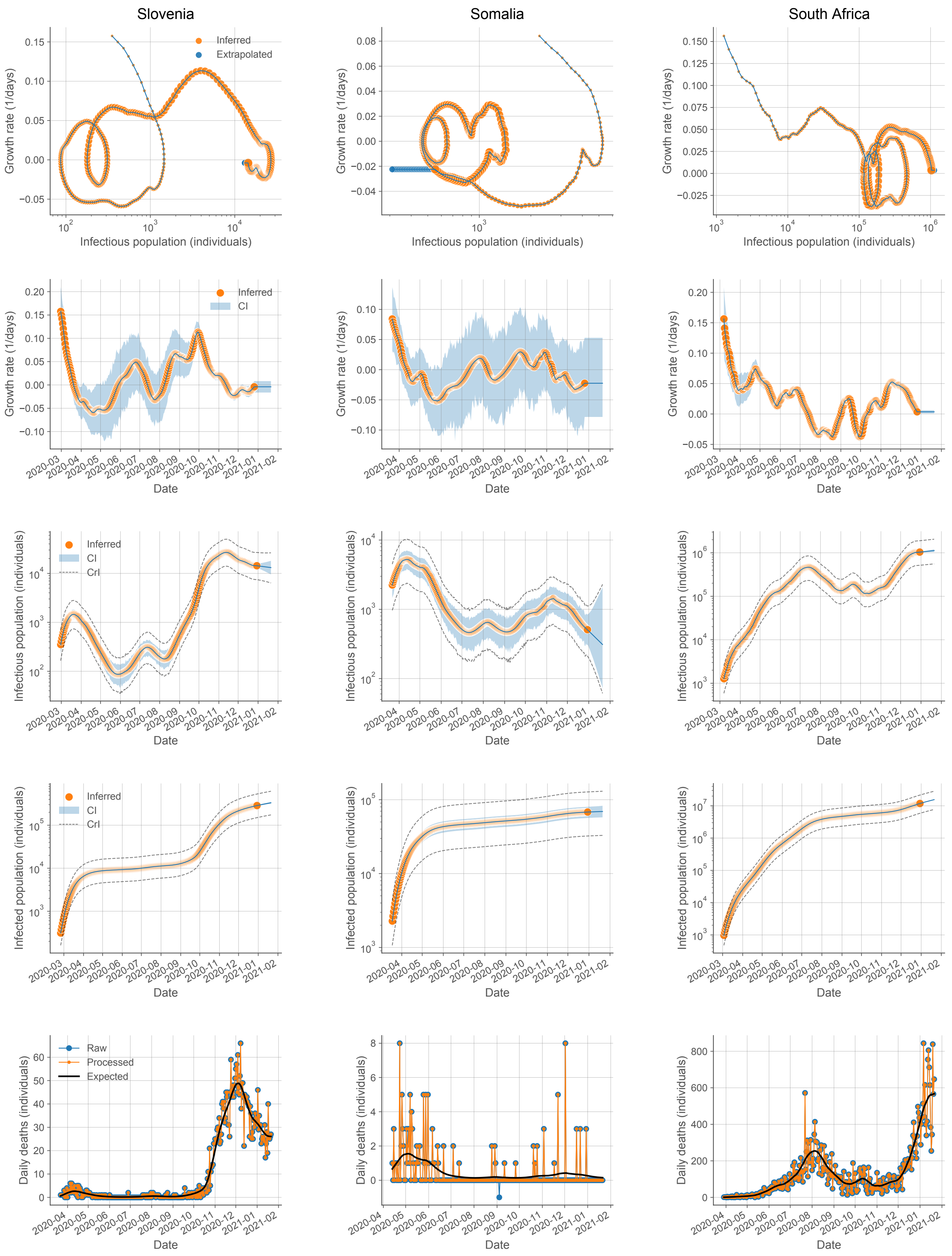

Slovenia
Somalia
South Africa
Growth rate (1/days)
Infectious population (individuals)
Inferred
Extrapolated
CI
CrI
Date
Infected population (individuals)
Daily deaths (individuals)
Raw
Processed
Expected

Figure S1.42

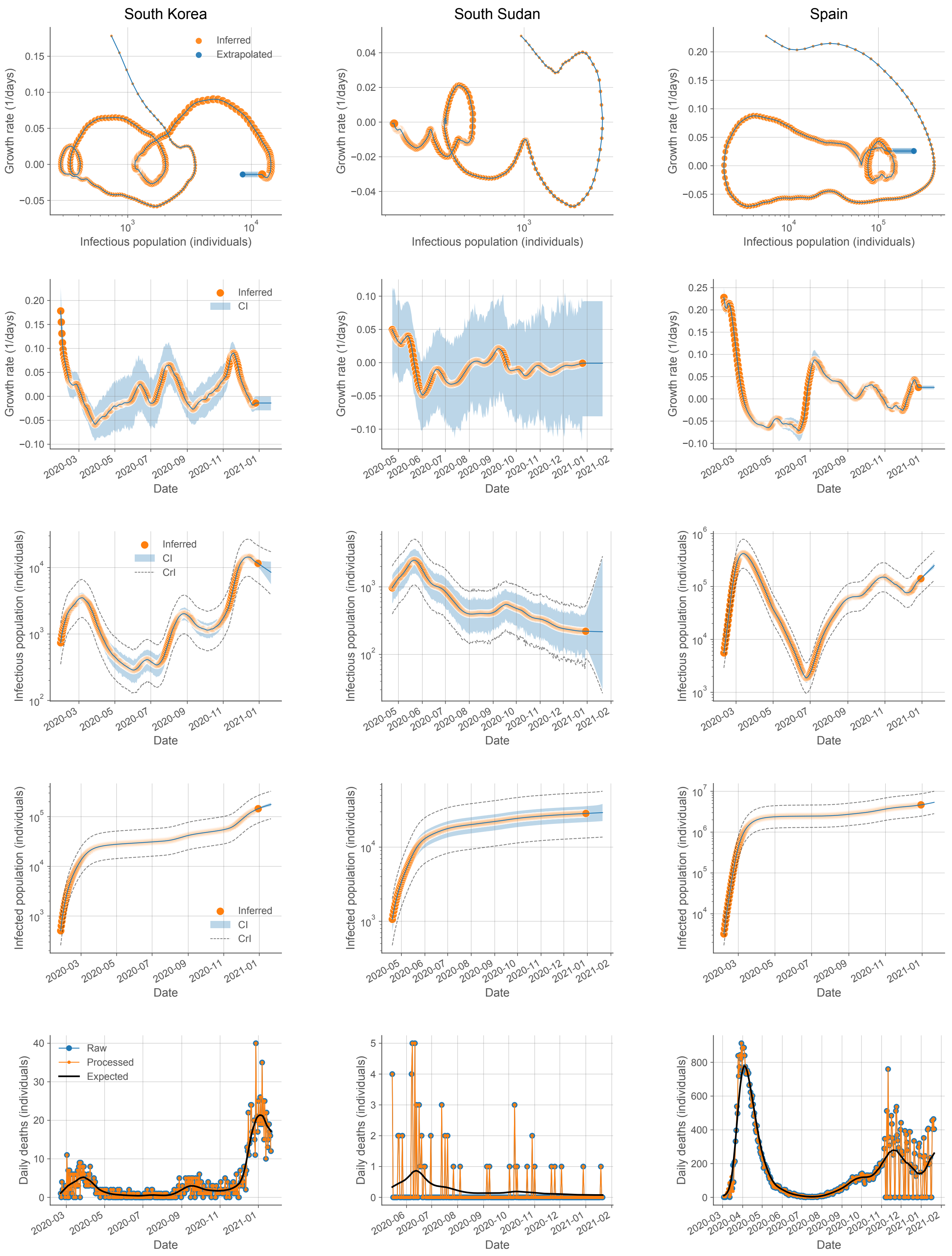

South Korea
South Sudan
Spain
Inferred
Extrapolated
CI
CrI
Raw
Processed
Expected
Growth rate (1/days)
Infectious population (individuals)
Infected population (individuals)
Daily deaths (individuals)
Date

Figure S1.43

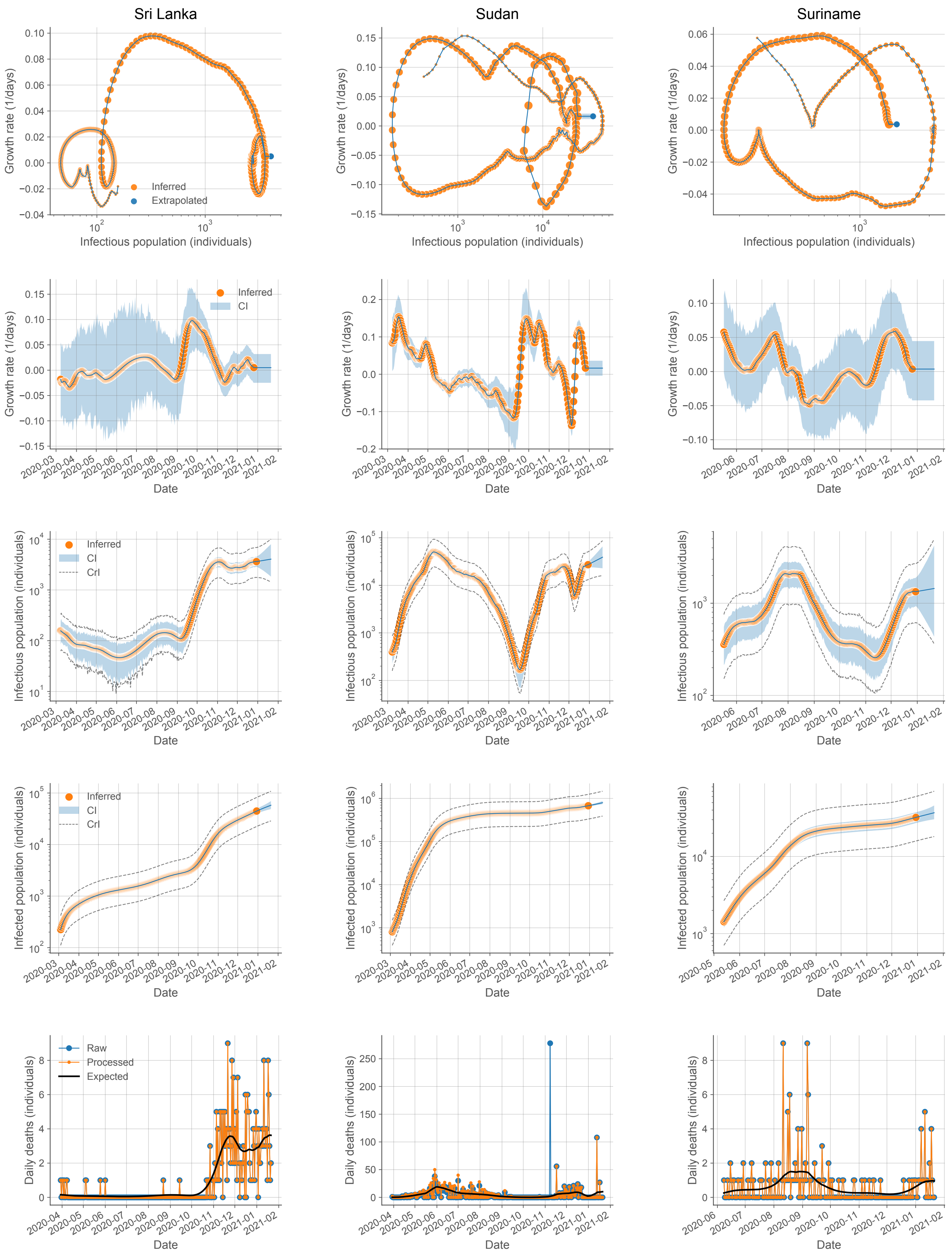

Sri Lanka
Sudan
Suriname
Growth rate (1/days)
Infectious population (individuals)
Inferred
Extrapolated
CI
CrI
Date
Infected population (individuals)
Daily deaths (individuals)
Raw
Processed
Expected

Figure S1.44

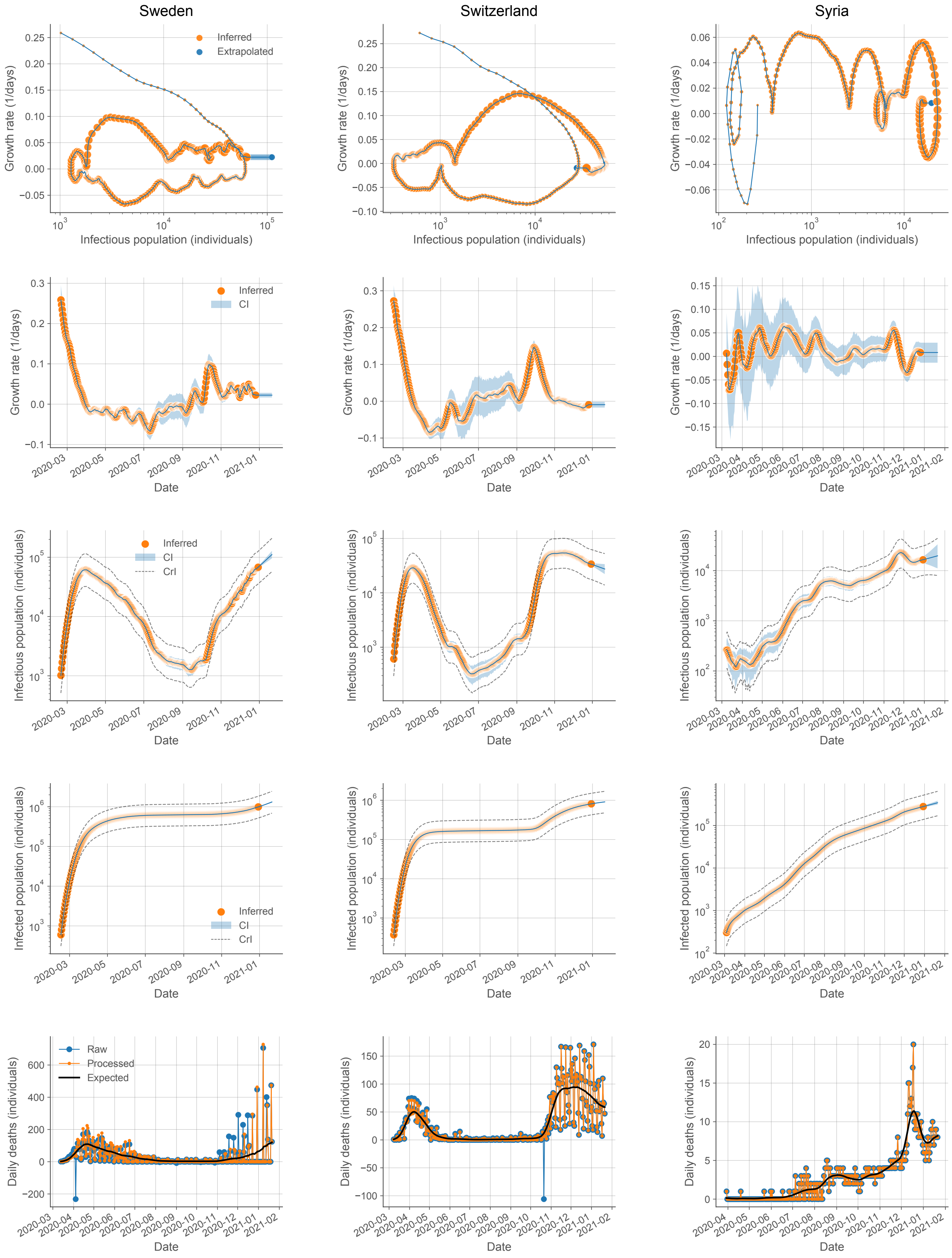
Sweden
Switzerland
Syria
Growth rate (1/days)
Infectious population (individuals)
Inferred
Extrapolated
CI
CrI
Date
Infected population (individuals)
Daily deaths (individuals)
Raw
Processed
Expected

Figure S1.45

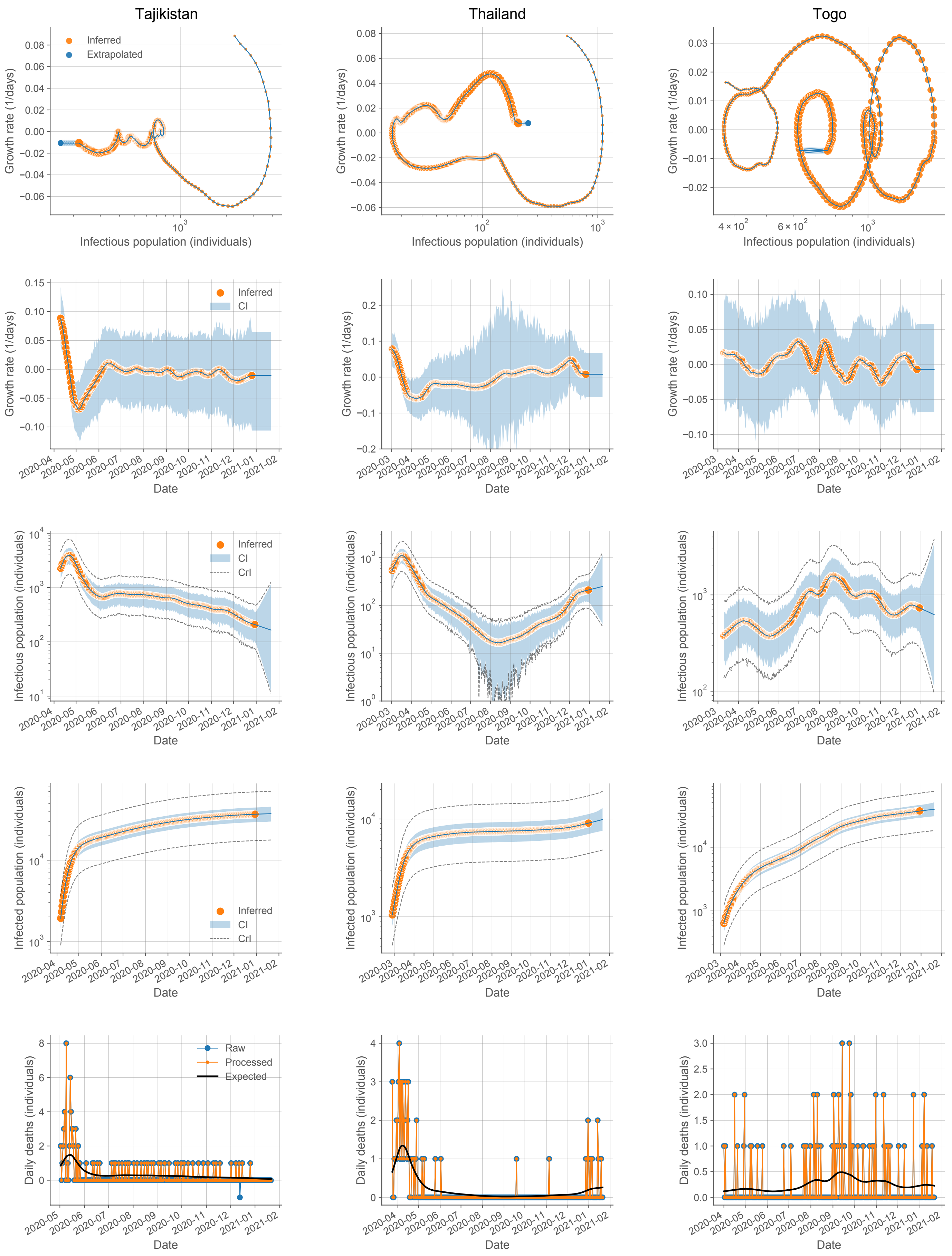

Tajikistan
Thailand
Togo
Inferred
Extrapolated
CI
CrI
Raw
Processed
Expected
Growth rate (1/days)
Infectious population (individuals)
Infected population (individuals)
Daily deaths (individuals)
Date

Figure S1.46

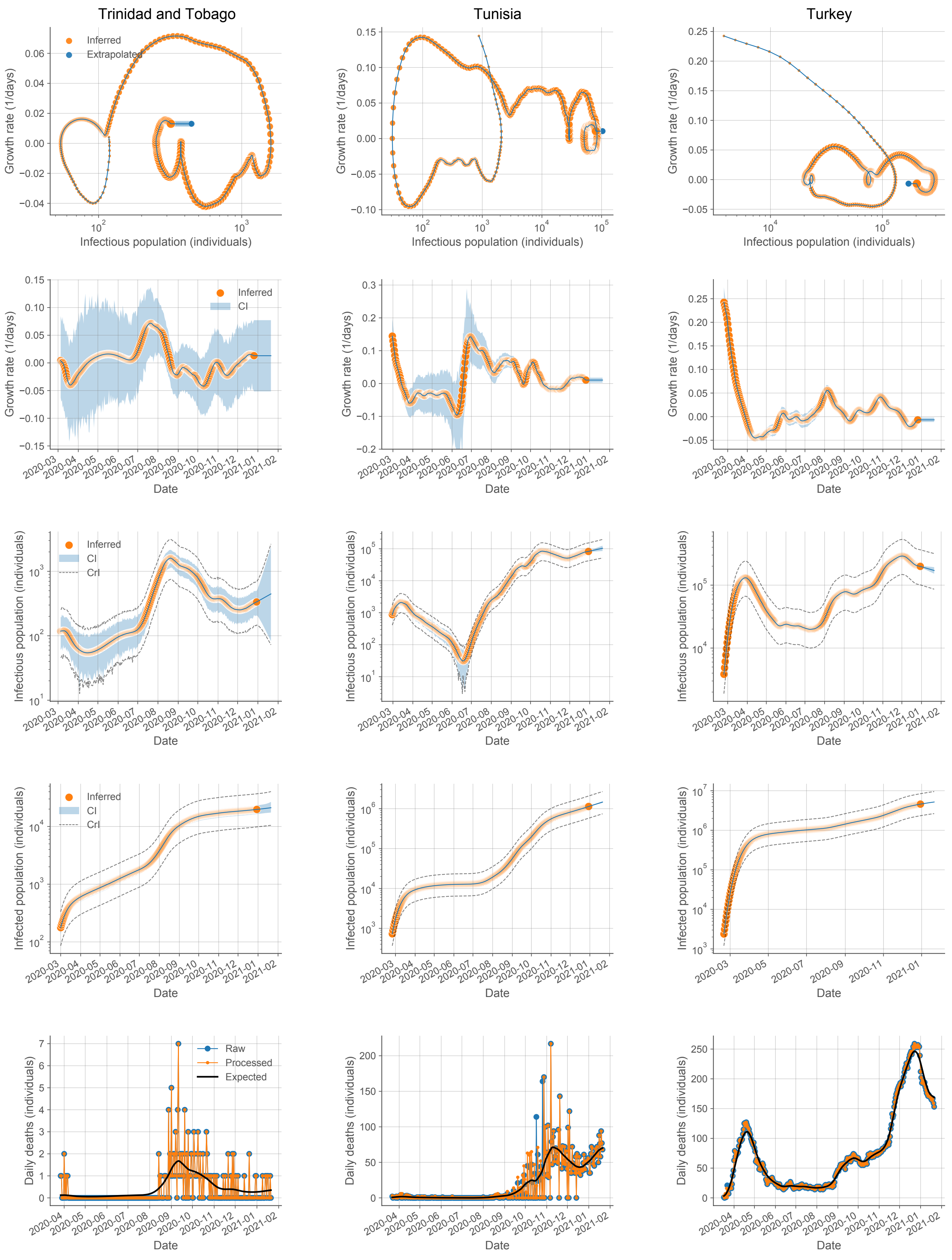

Trinidad and Tobago
Tunisia
Turkey
Inferred
Extrapolated
CI
CrI
Raw
Processed
Expected
Growth rate (1/days)
Infectious population (individuals)
Infected population (individuals)
Daily deaths (individuals)
Date

Figure S1.47

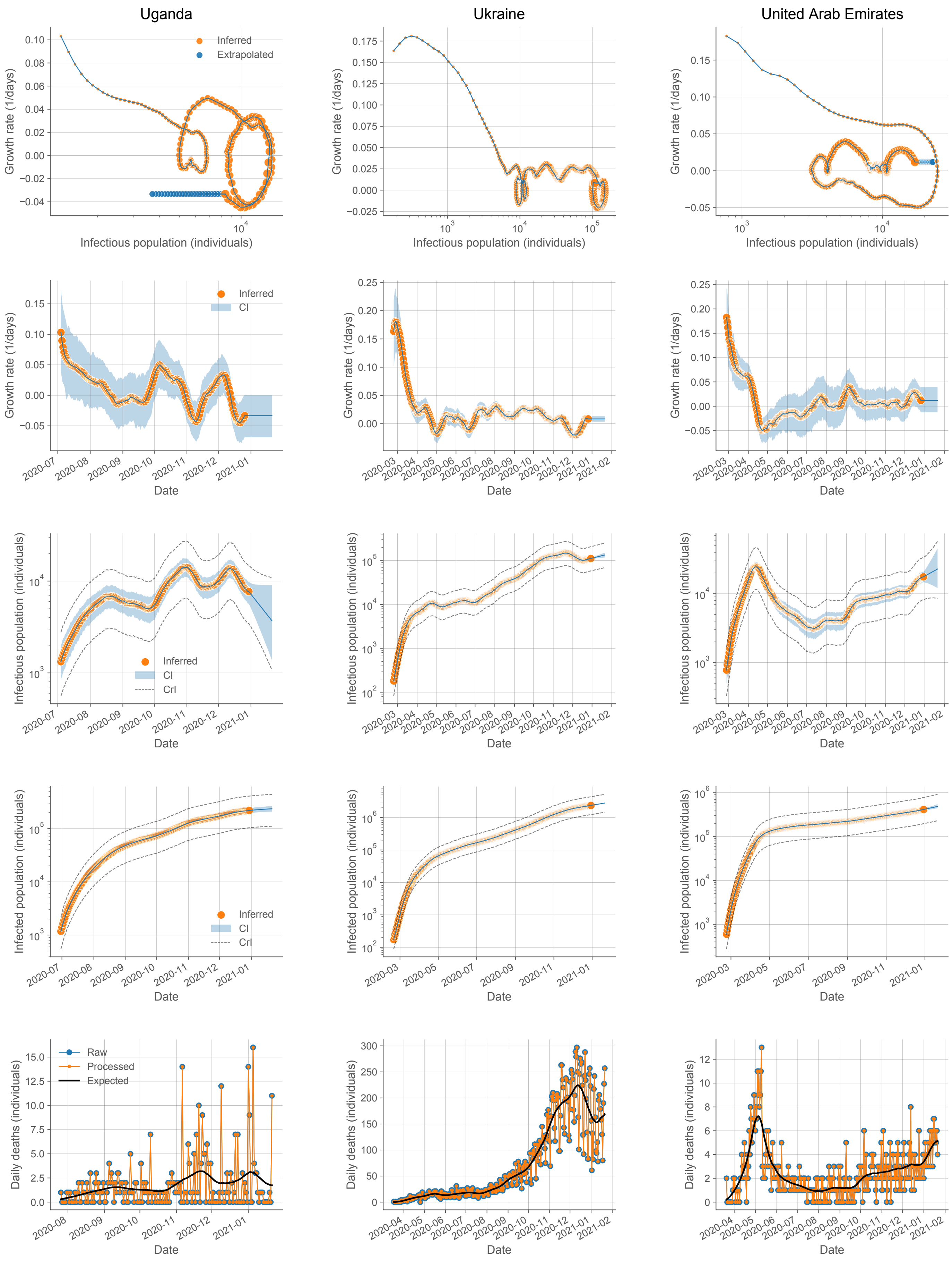
Uganda
Ukraine
United Arab Emirates
Growth rate (1/days)
Infectious population (individuals)
Inferred
Extrapolated
CI
CrI
Date
Infected population (individuals)
Daily deaths (individuals)
Raw
Processed
Expected

Figure S1.48

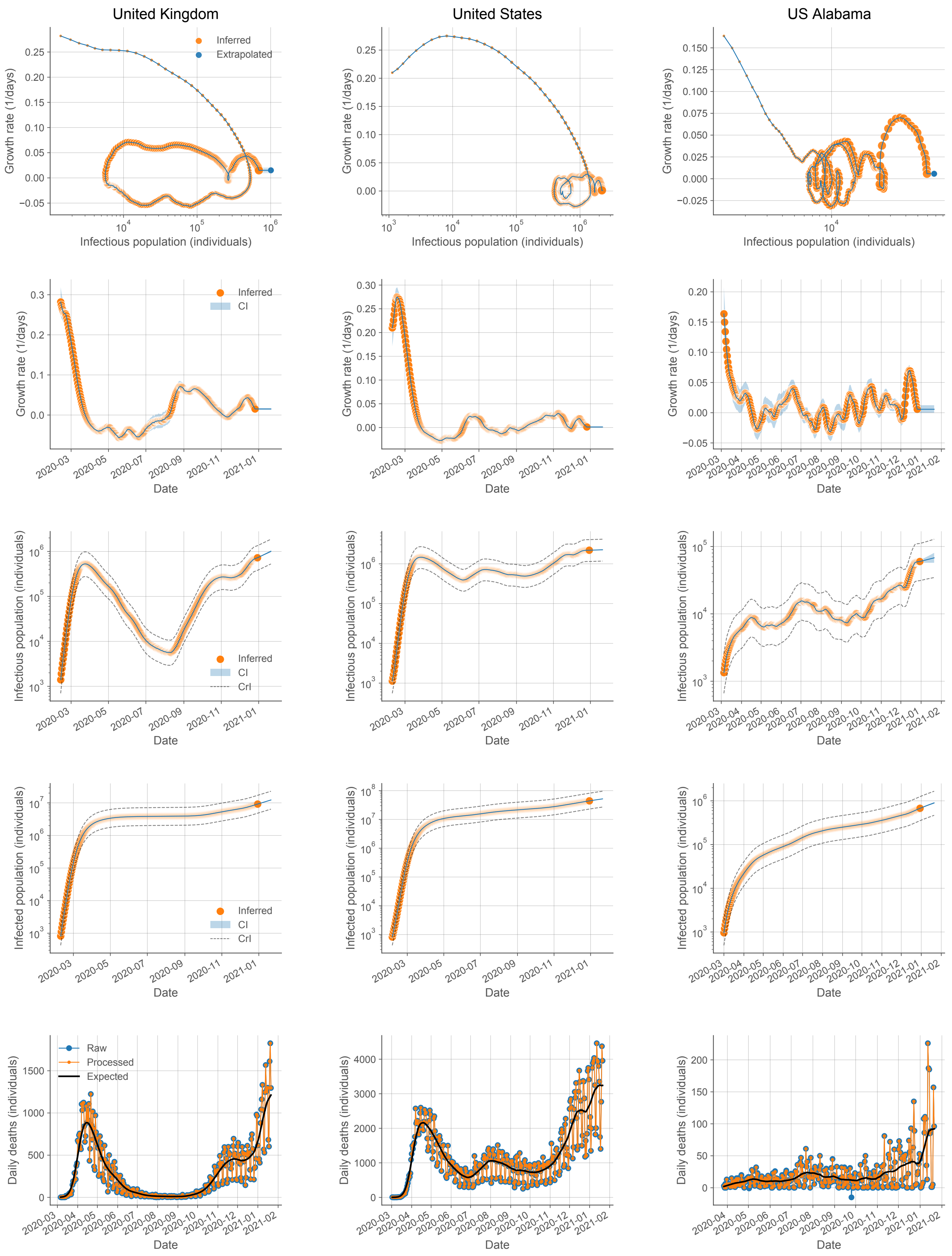
United Kingdom
United States
US Alabama
Growth rate (1/days)
Infectious population (individuals)
Inferred
Extrapolated
CI
CrI
Date
Infected population (individuals)
Daily deaths (individuals)
Raw
Processed
Expected

Figure S1.49

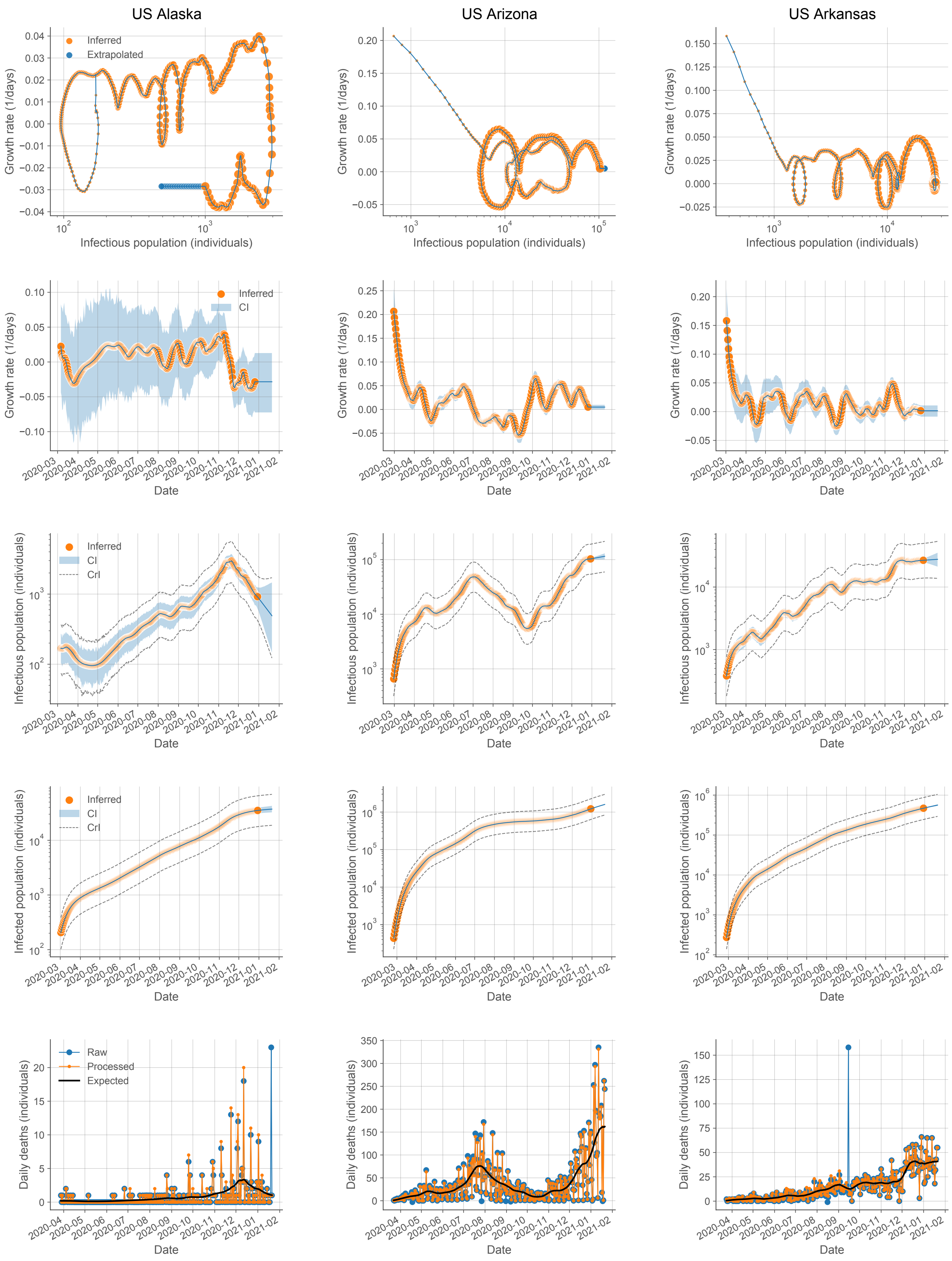

US Alaska
US Arizona
US Arkansas
Inferred
Extrapolated
CI
CrI
Raw
Processed
Expected
Growth rate (1/days)
Infectious population (individuals)
Infected population (individuals)
Daily deaths (individuals)
Date

Figure S1.50

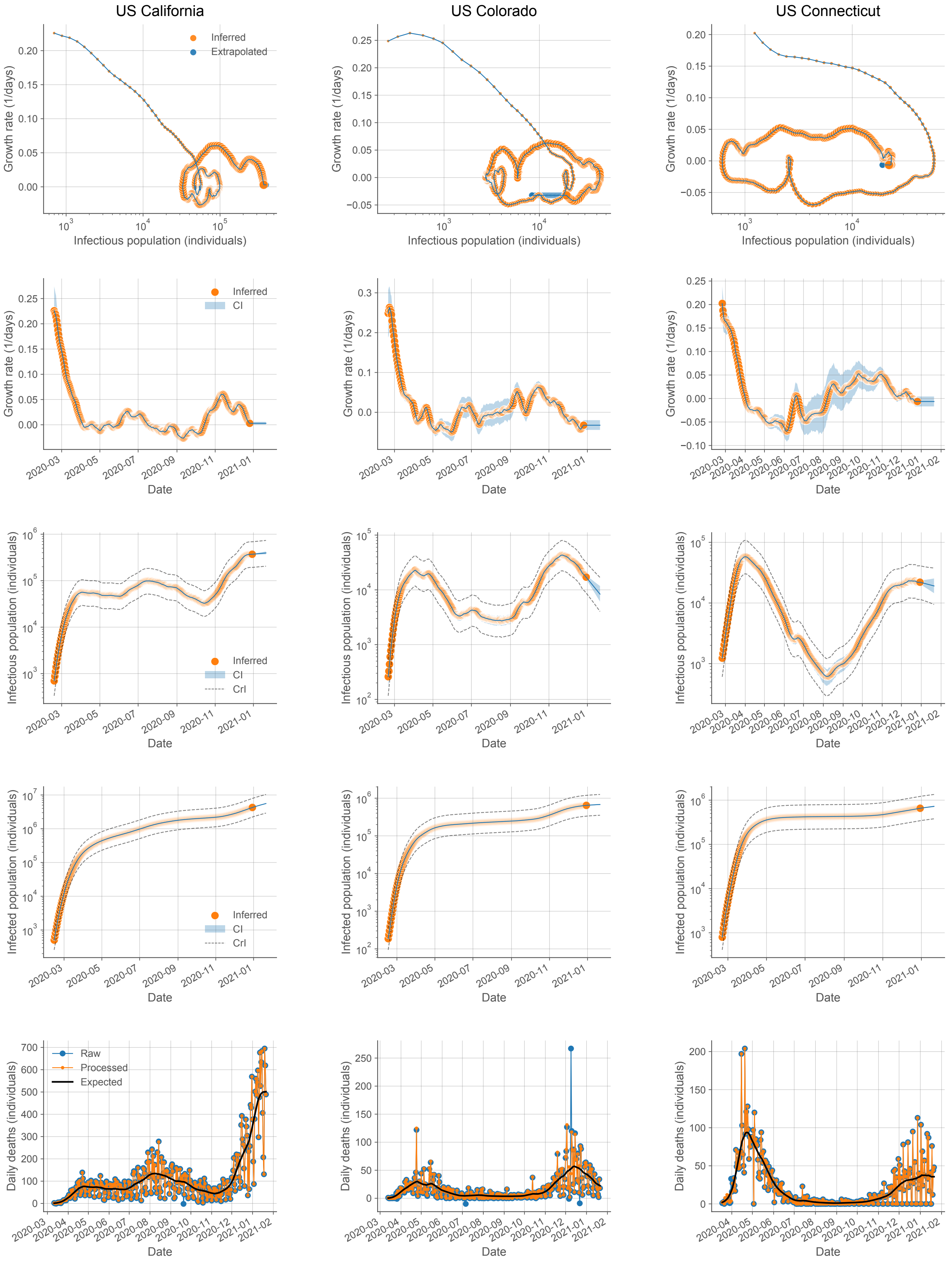
US California
US Colorado
US Connecticut
Growth rate (1/days)
Infectious population (individuals)
Inferred
Extrapolated
CI
CrI
Date
Infected population (individuals)
Daily deaths (individuals)
Raw
Processed
Expected

Figure S1.51

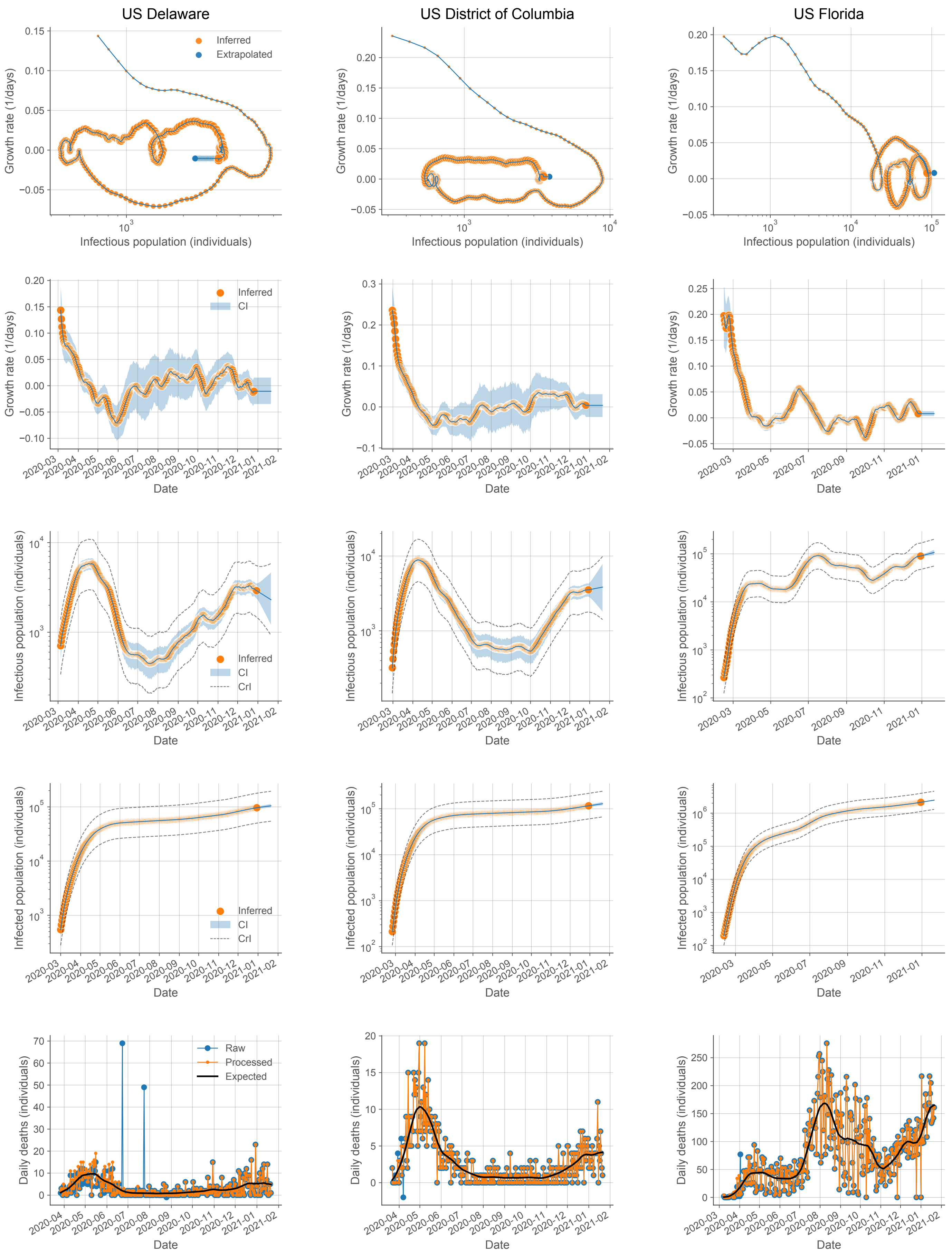
US Delaware
US District of Columbia
US Florida
Inferred
Extrapolated
CI
CrI
Raw
Processed
Expected
Growth rate (1/days)
Infectious population (individuals)
Infected population (individuals)
Daily deaths (individuals)
Date

Figure S1.52

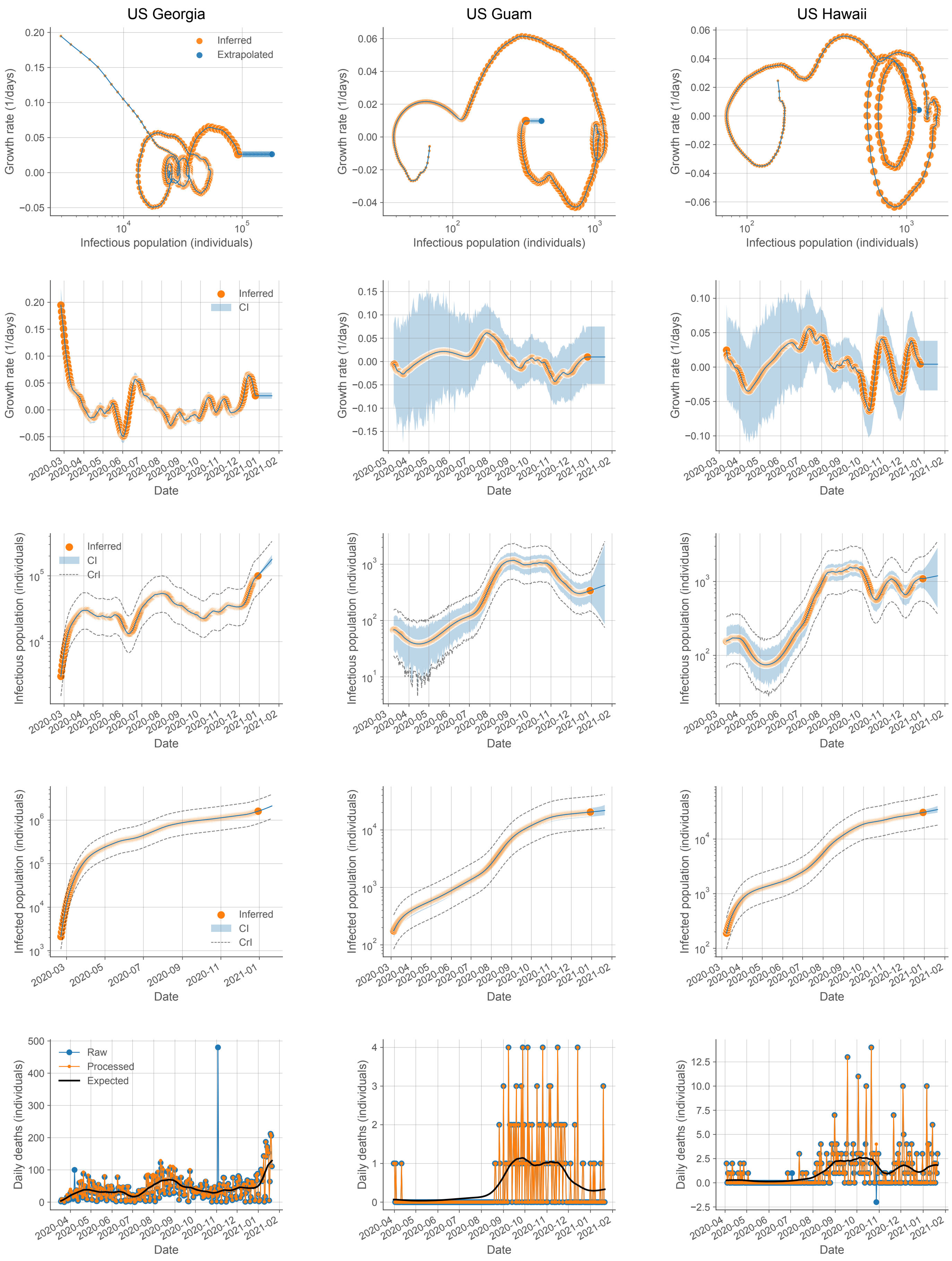
US Georgia
US Guam
US Hawaii
Inferred
Extrapolated
CI
CrI
Raw
Processed
Expected
Growth rate (1/days)
Infectious population (individuals)
Infected population (individuals)
Daily deaths (individuals)
Date

Figure S1.53

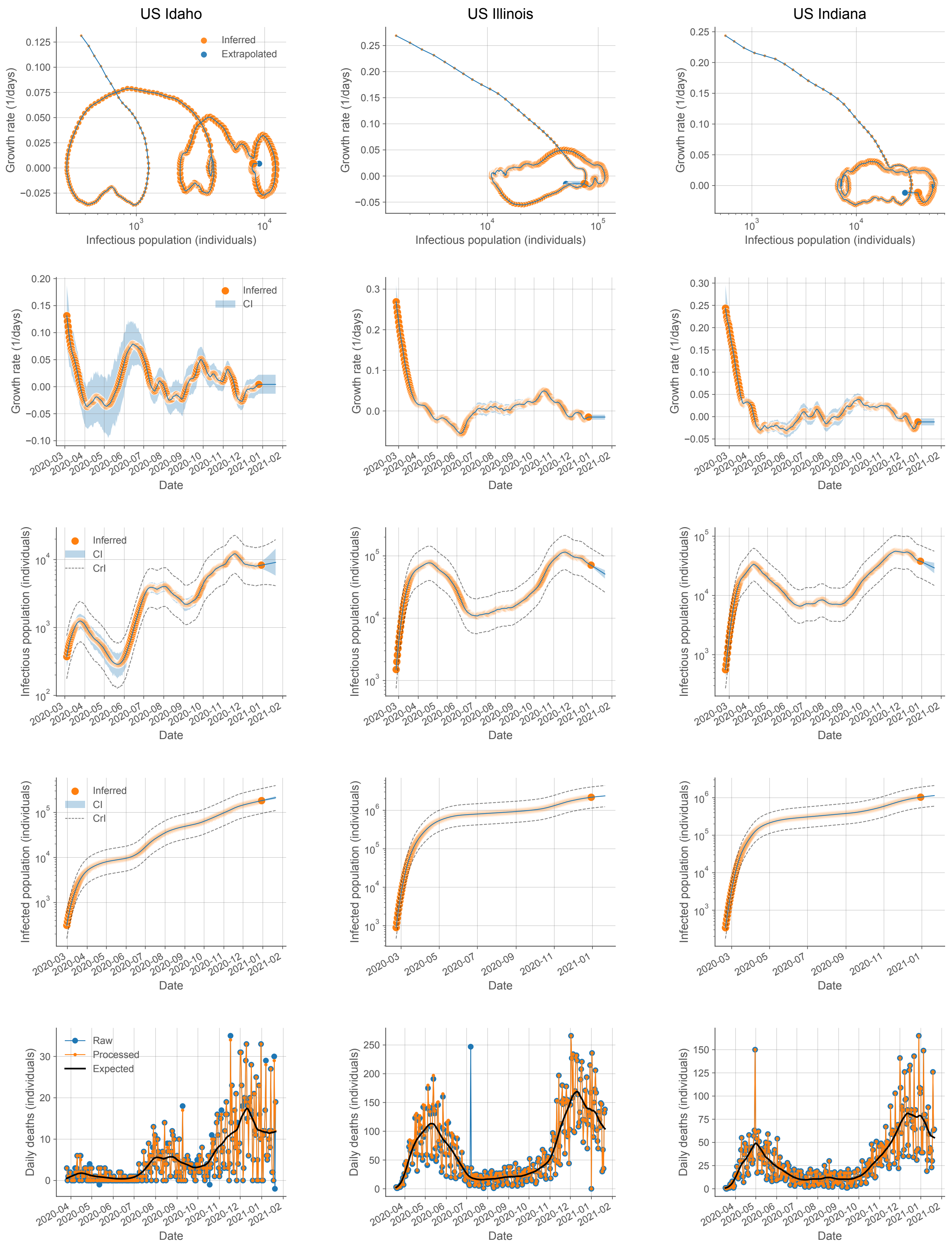
US Idaho
US Illinois
US Indiana
Inferred
Extrapolated
CI
CrI
Raw
Processed
Expected
Growth rate (1/days)
Infectious population (individuals)
Infected population (individuals)
Daily deaths (individuals)
Date

Figure S1.54

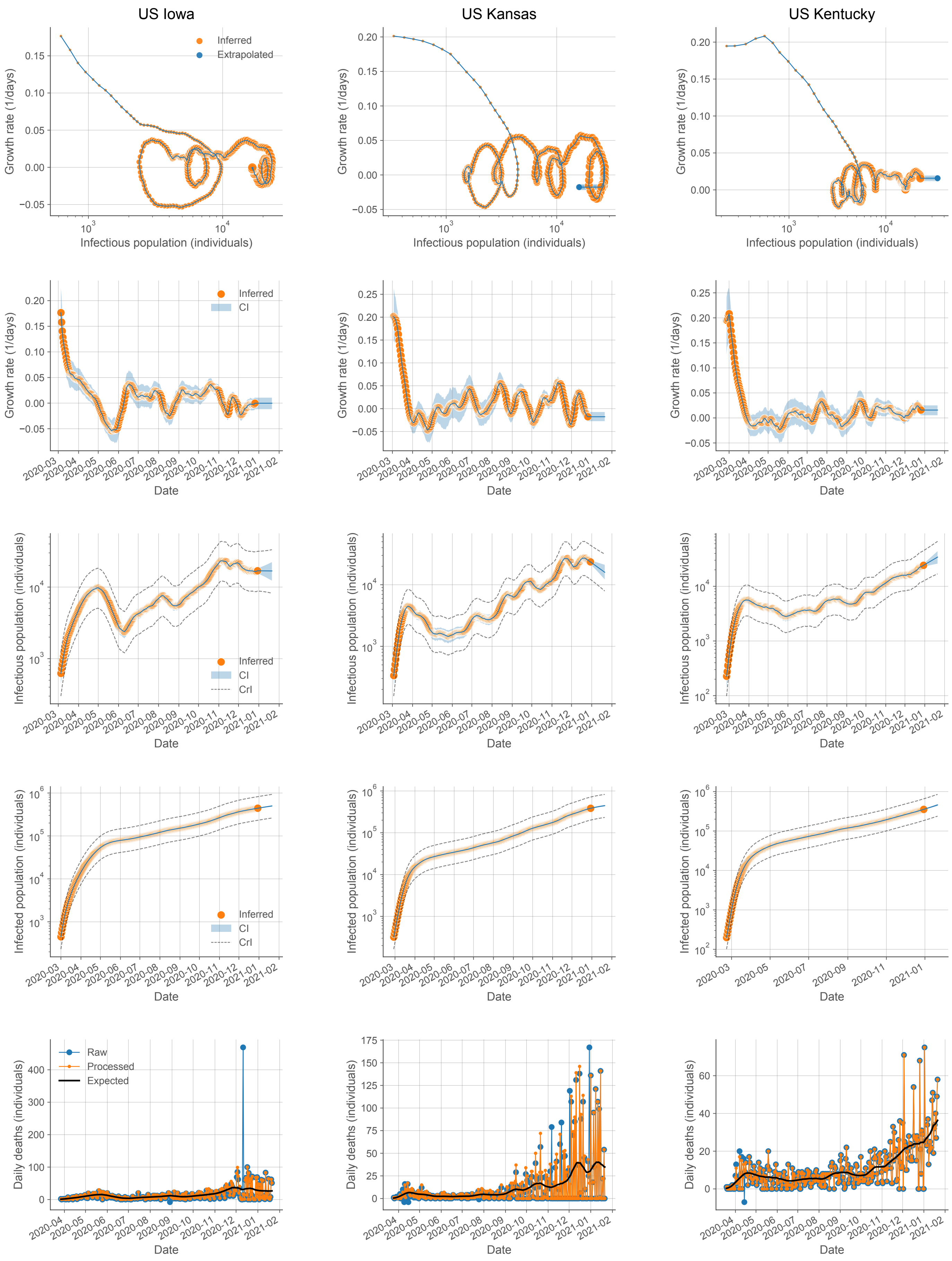
US Iowa
US Kansas
US Kentucky
Inferred
Extrapolated
Growth rate (1/days)
Infectious population (individuals)
Inferred
CI
Growth rate (1/days)
Date
Inferred
CI
CrI
Infectious population (individuals)
Date
Inferred
CI
CrI
Infected population (individuals)
Date
Raw
Processed
Expected
Daily deaths (individuals)
Date

Figure S1.55

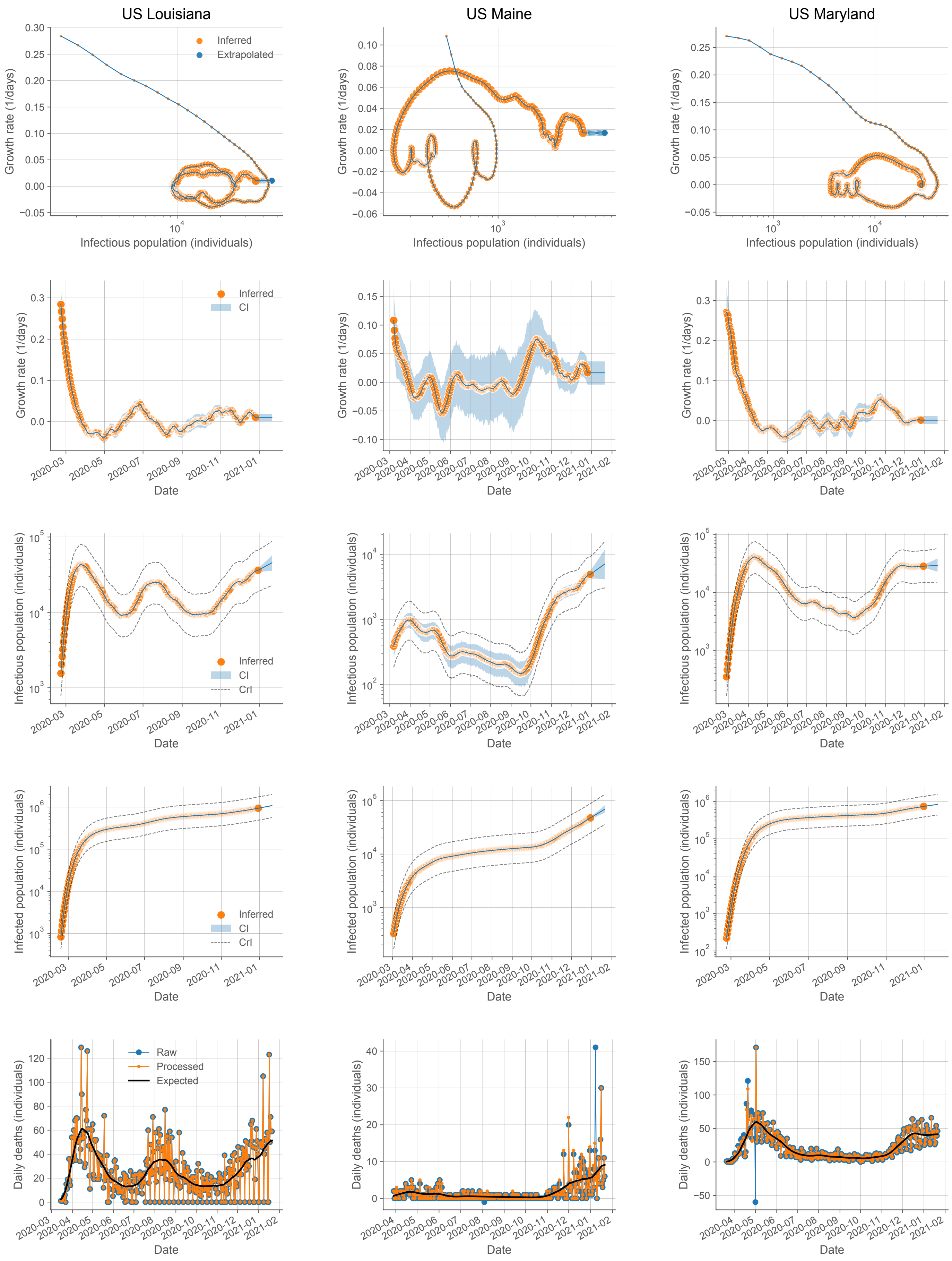
US Louisiana
US Maine
US Maryland
Inferred
Extrapolated
CI
CrI
Raw
Processed
Expected
Growth rate (1/days)
Infectious population (individuals)
Infected population (individuals)
Daily deaths (individuals)
Date

Figure S1.56

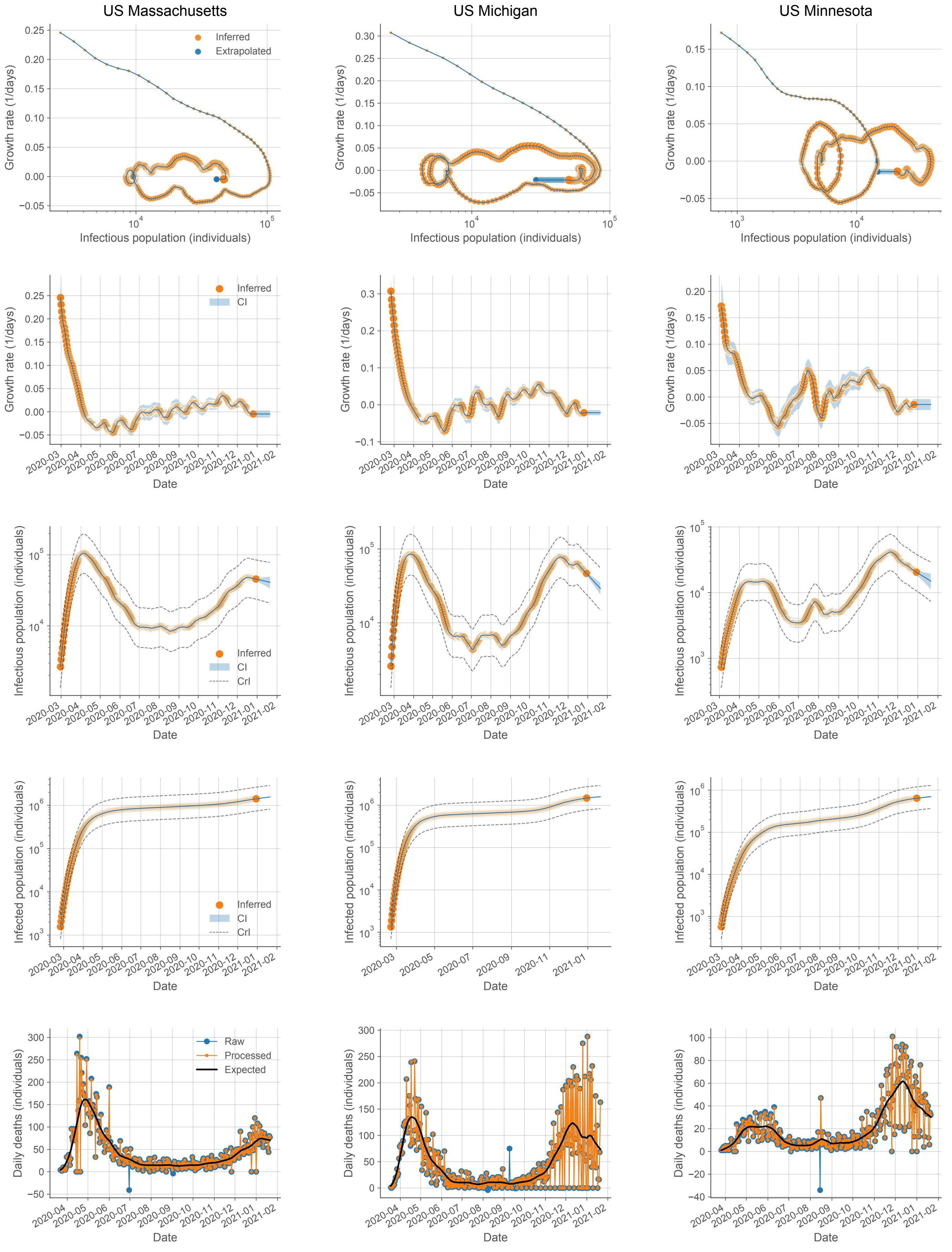

US Massachusetts
US Michigan
US Minnesota
Inferred
Extrapolated
CI
CrI
Raw
Processed
Expected
Growth rate (1/days)
Infectious population (individuals)
Infected population (individuals)
Daily deaths (individuals)
Date

Figure S1.57

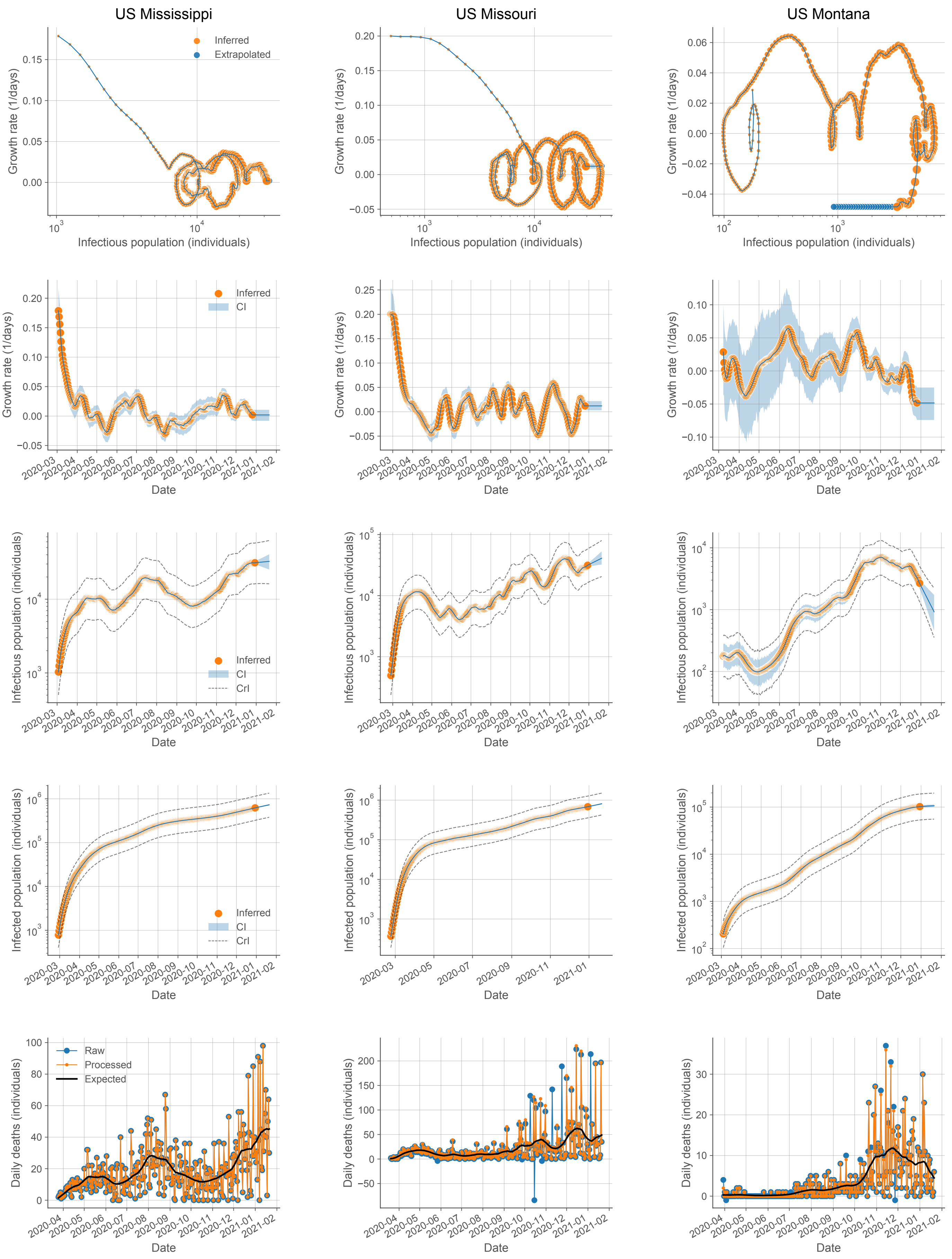
US Mississippi
US Missouri
US Montana
Growth rate (1/days)
Infectious population (individuals)
Inferred
Extrapolated
CI
CrI
Date
Infected population (individuals)
Daily deaths (individuals)
Raw
Processed
Expected

Figure S1.58

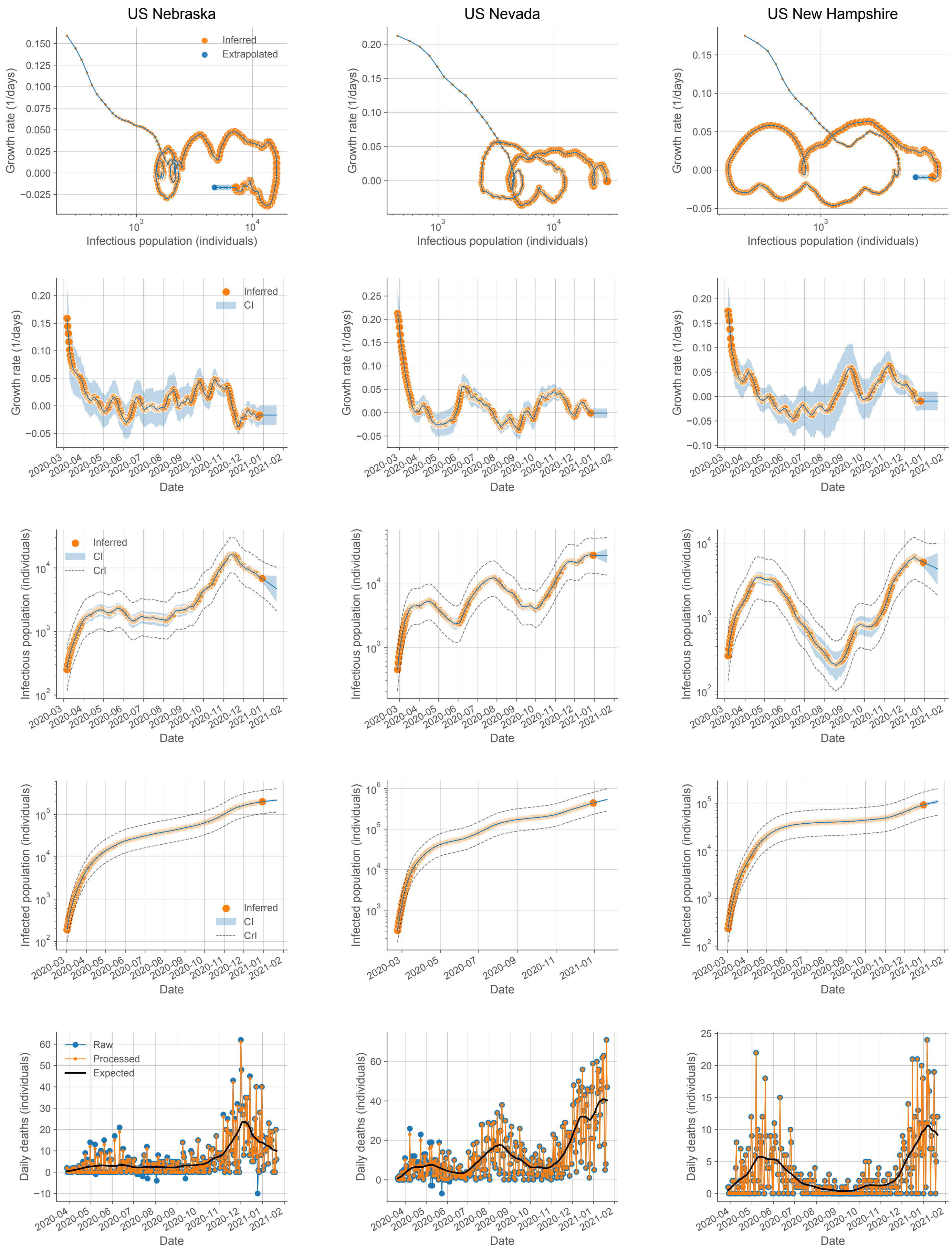
US Nebraska
US Nevada
US New Hampshire
Growth rate (1/days)
Infectious population (individuals)
Inferred
Extrapolated
CI
CrI
Date
Infected population (individuals)
Daily deaths (individuals)
Raw
Processed
Expected

Figure S1.59

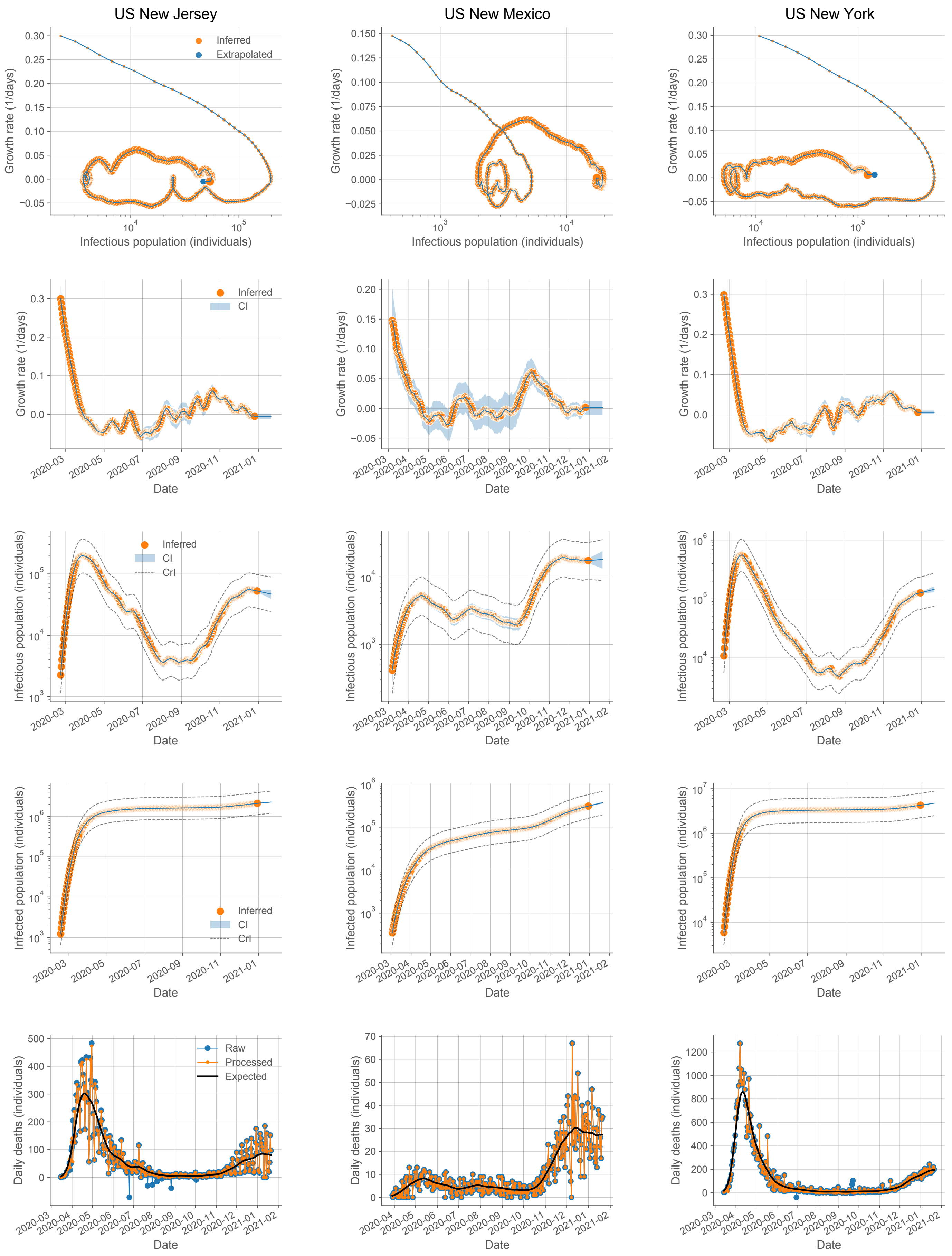

US New Jersey
US New Mexico
US New York
Growth rate (1/days)
Infectious population (individuals)
Inferred
Extrapolated
CI
CrI
Date
Infected population (individuals)
Daily deaths (individuals)
Raw
Processed
Expected

Figure S1.60

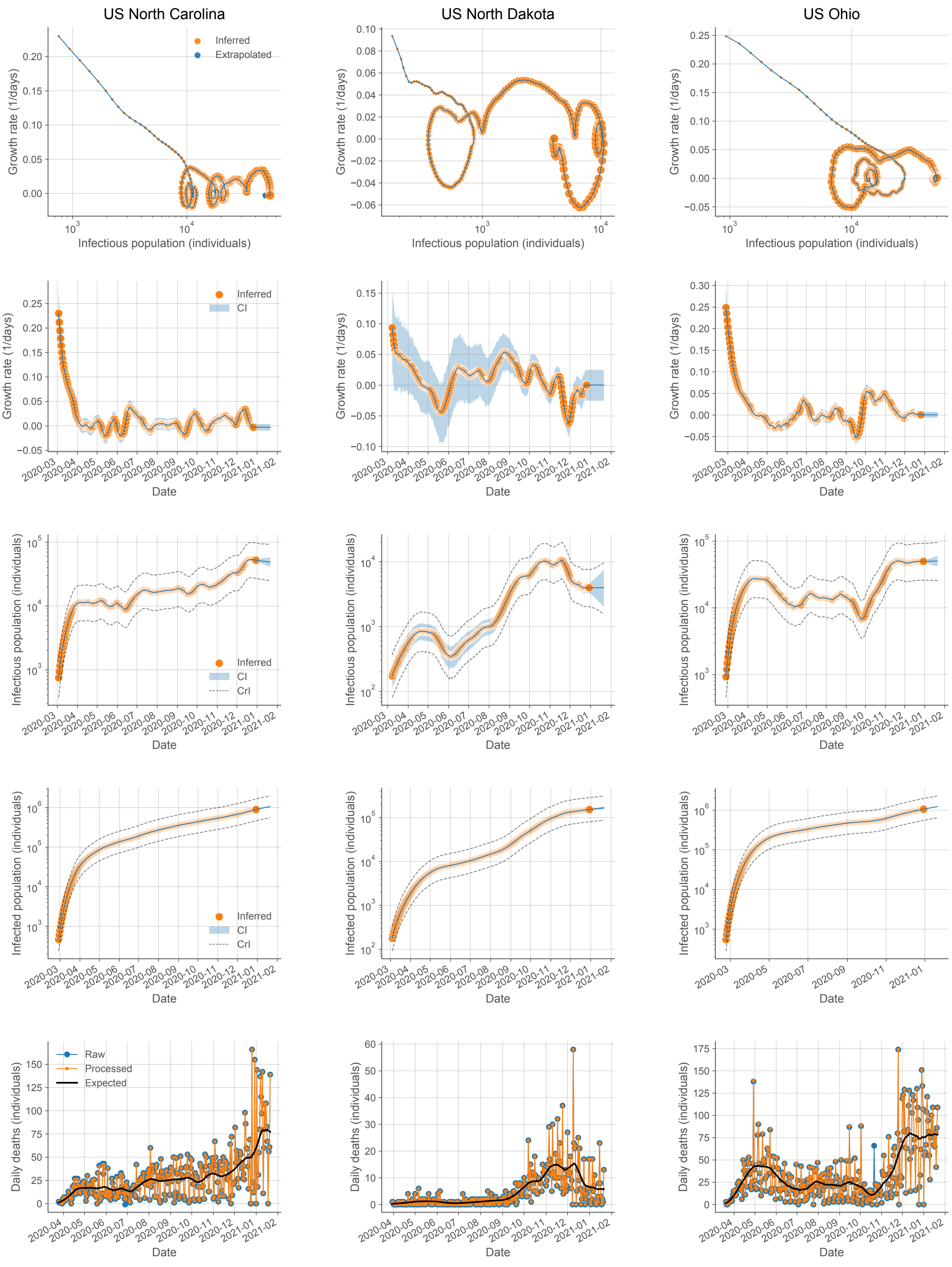

US North Carolina
US North Dakota
US Ohio
Inferred
Extrapolated
CI
CrI
Raw
Processed
Expected
Growth rate (1/days)
Infectious population (individuals)
Infected population (individuals)
Daily deaths (individuals)
Date

Figure S1.61

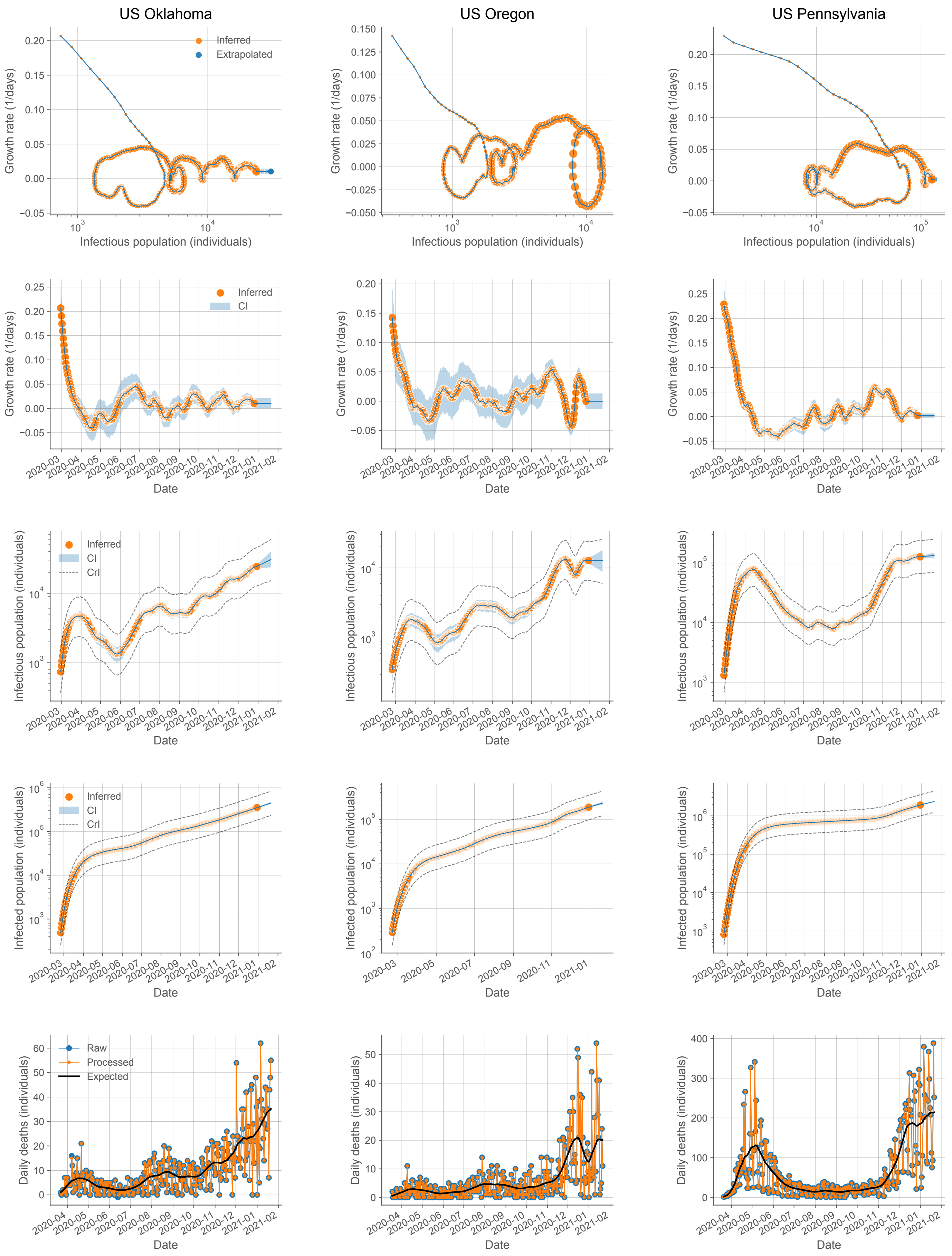

US Oklahoma
US Oregon
US Pennsylvania
Inferred
Extrapolated
CI
CrI
Raw
Processed
Expected
Growth rate (1/days)
Infectious population (individuals)
Infected population (individuals)
Daily deaths (individuals)
Date

Figure S1.62

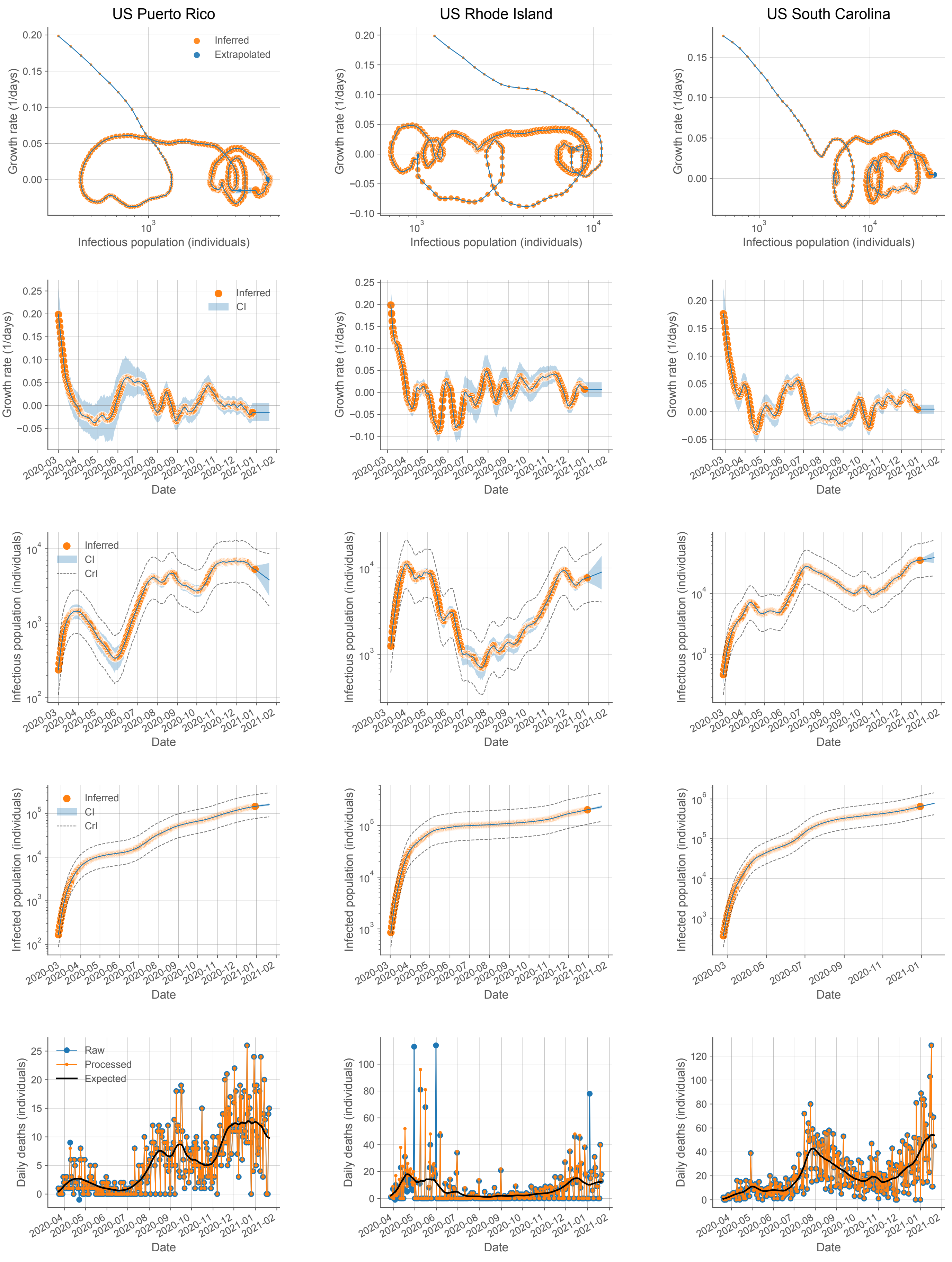

US Puerto Rico
US Rhode Island
US South Carolina
Inferred
Extrapolated
CI
CrI
Raw
Processed
Expected
Growth rate (1/days)
Infectious population (individuals)
Infected population (individuals)
Daily deaths (individuals)
Date

Figure S1.63

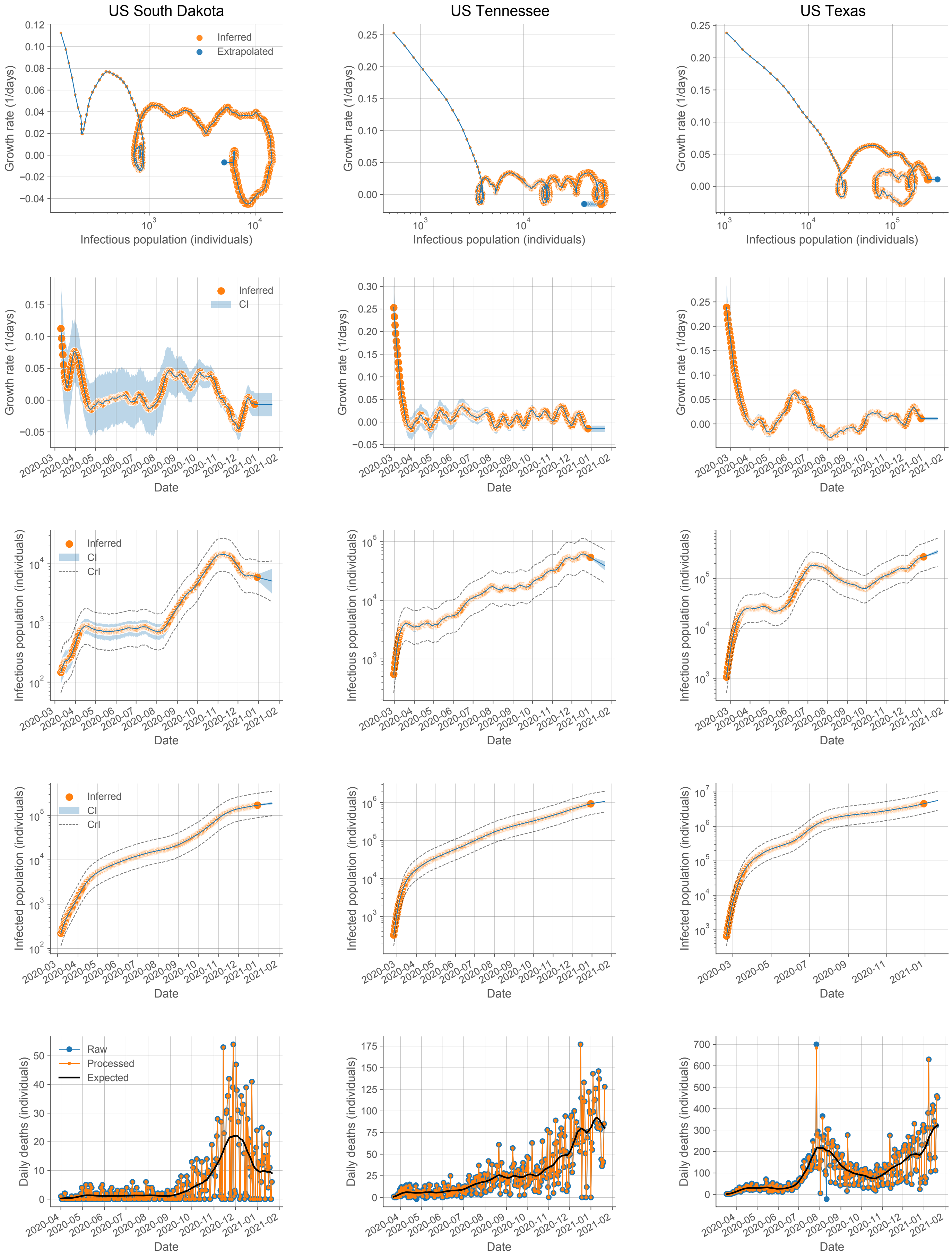
US South Dakota
US Tennessee
US Texas
Growth rate (1/days)
Infectious population (individuals)
Inferred
Extrapolated
CI
CrI
Date
Infected population (individuals)
Daily deaths (individuals)
Raw
Processed
Expected

Figure S1.64

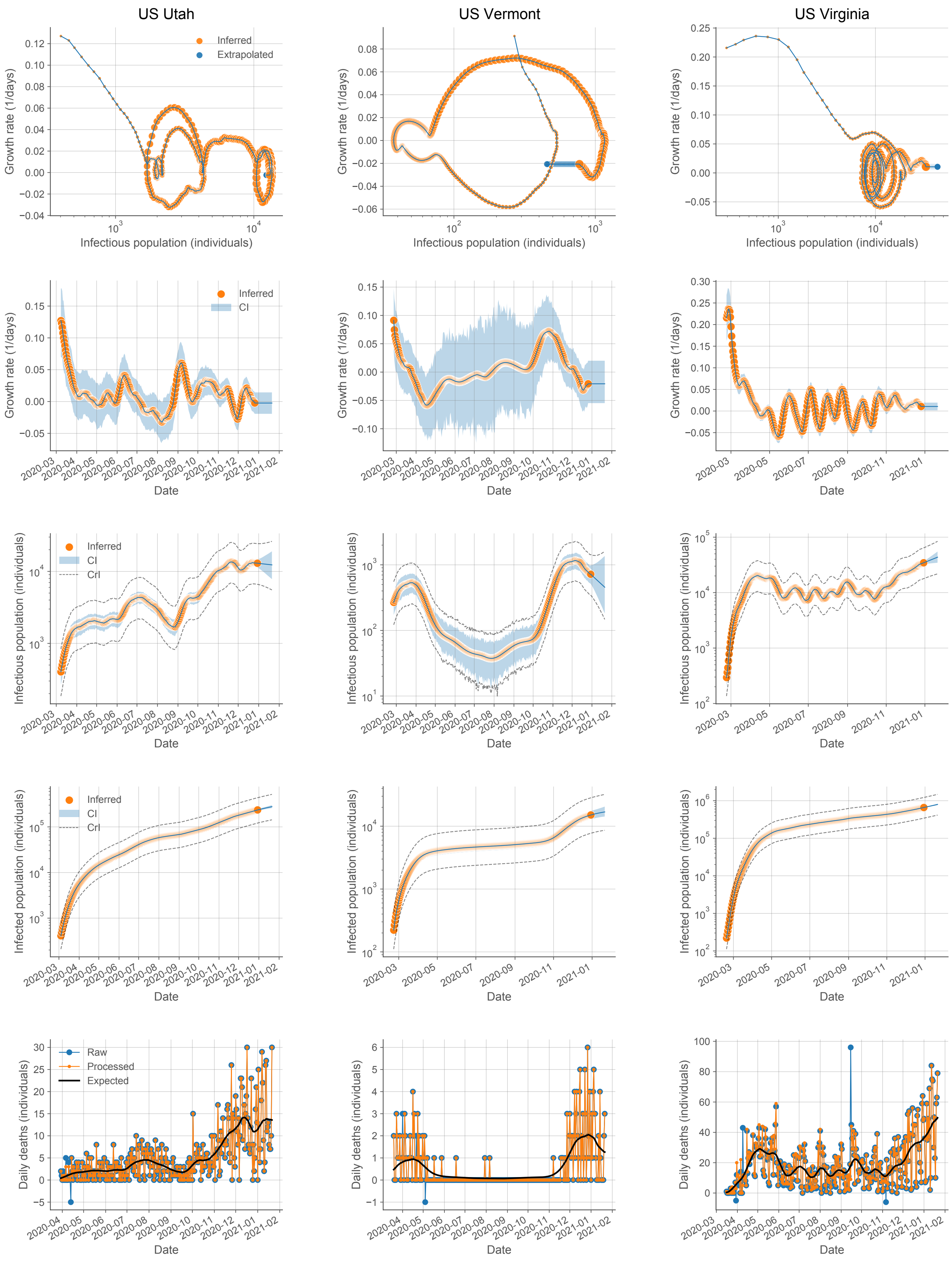
US Utah
US Vermont
US Virginia
Inferred
Extrapolated
CI
CrI
Raw
Processed
Expected
Growth rate (1/days)
Infectious population (individuals)
Infected population (individuals)
Daily deaths (individuals)
Date

Figure S1.65

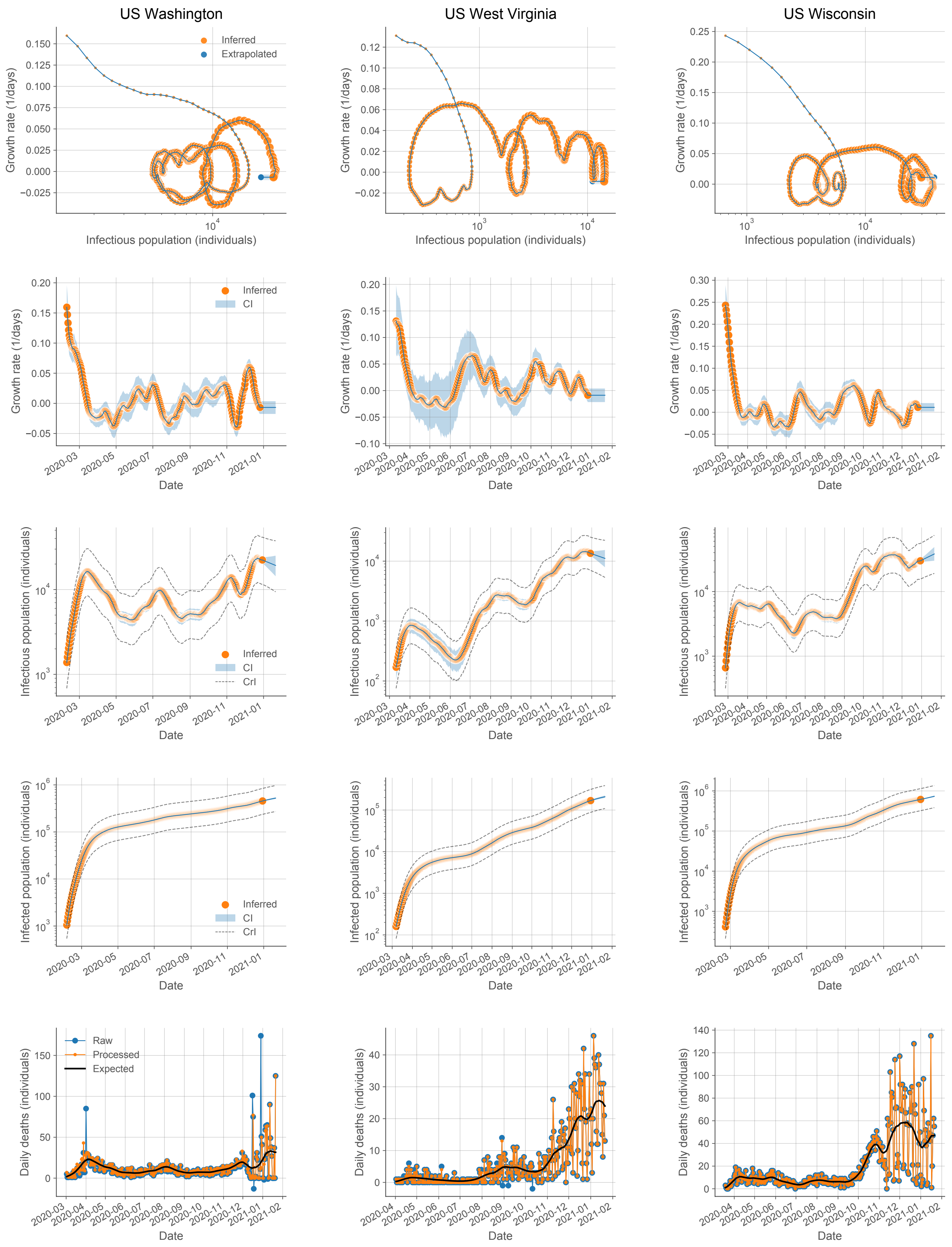
US Washington
US West Virginia
US Wisconsin
Growth rate (1/days)
Infectious population (individuals)
Infected population (individuals)
Daily deaths (individuals)
Date
Inferred
Extrapolated
CI
CrI
Raw
Processed
Expected

Figure S1.66

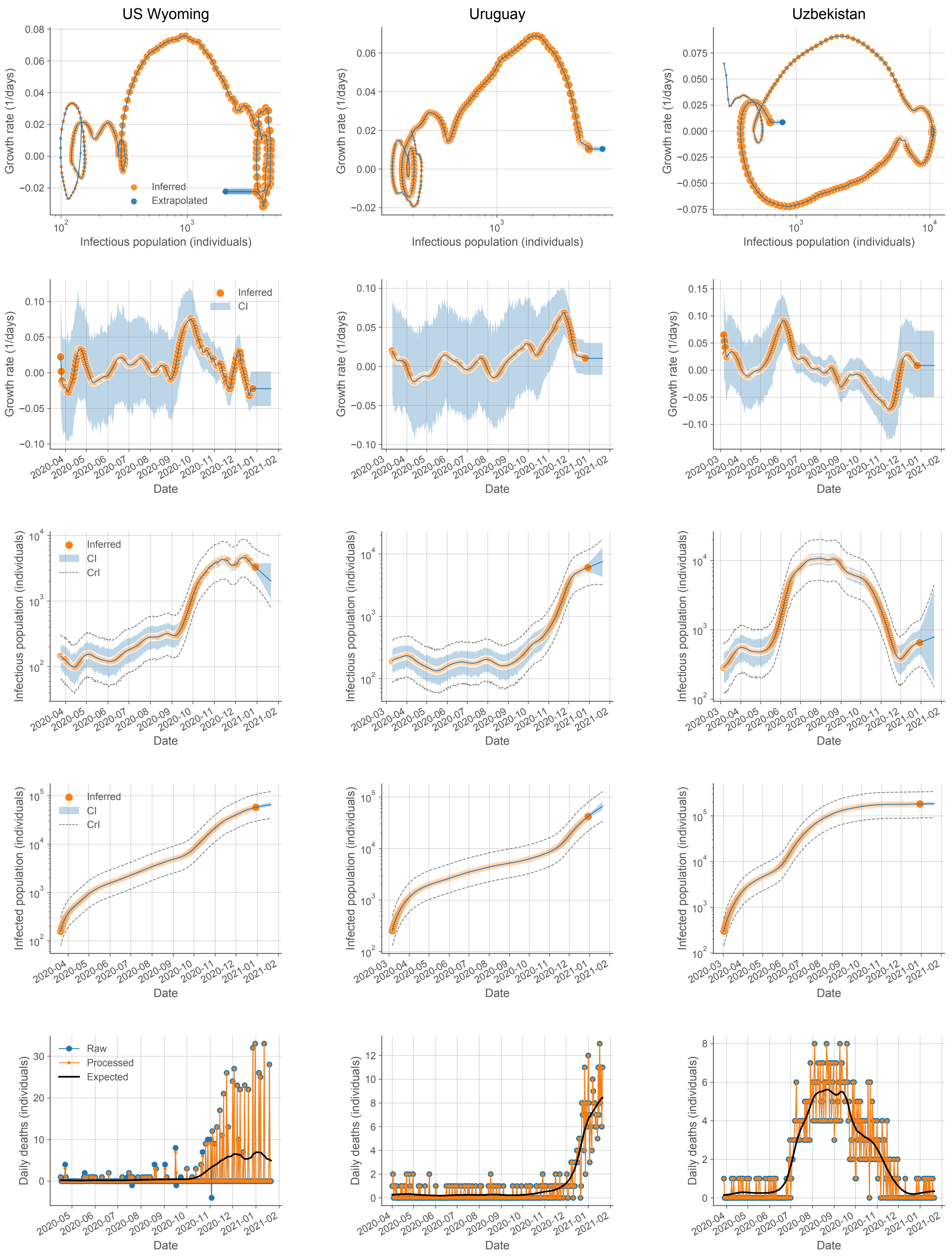

US Wyoming
Uruguay
Uzbekistan
Growth rate (1/days)
Infectious population (individuals)
Inferred
Extrapolated
CI
CrI
Date
Infected population (individuals)
Daily deaths (individuals)
Raw
Processed
Expected

Figure S1.67

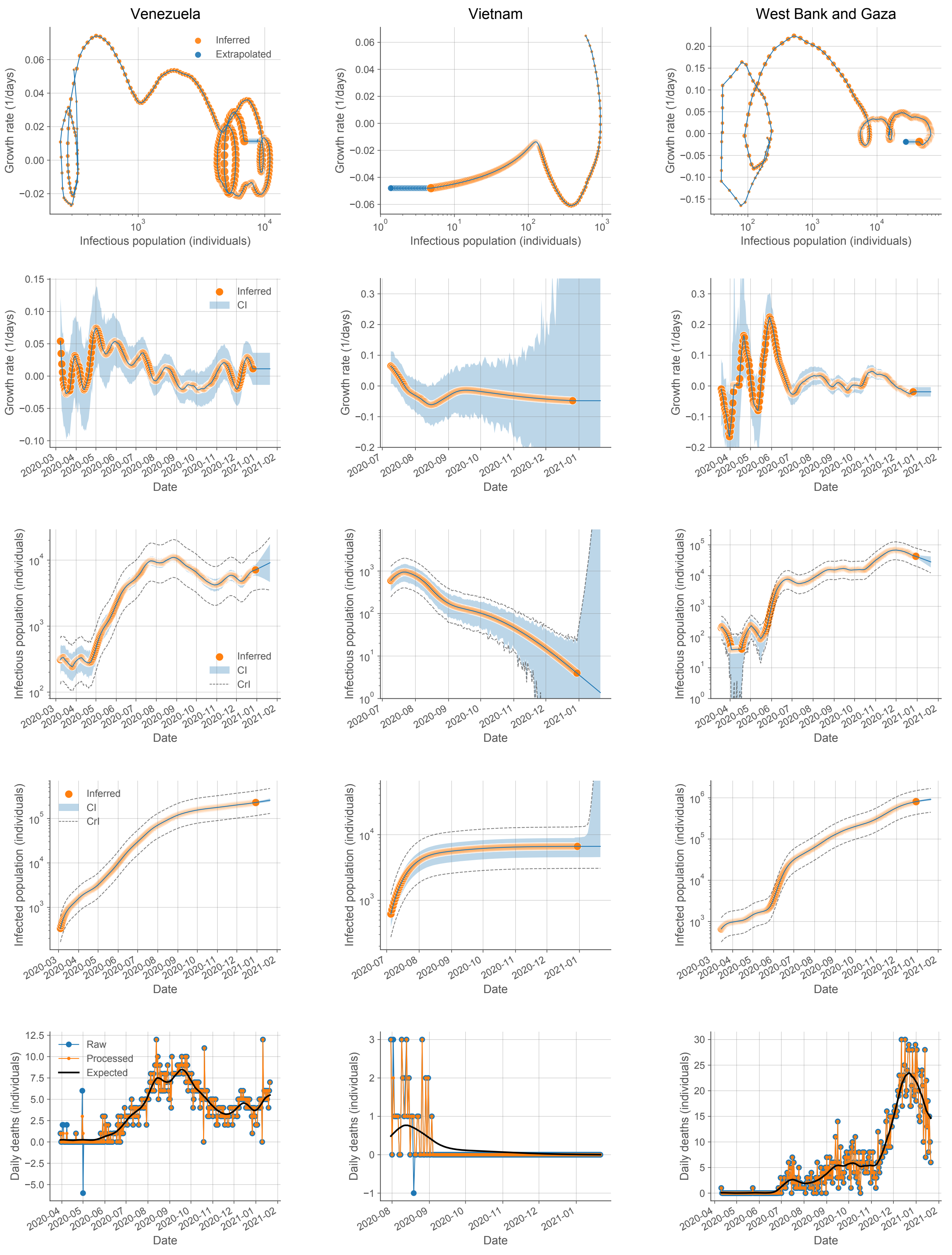

Venezuela
Vietnam
West Bank and Gaza
Growth rate (1/days)
Infectious population (individuals)
Inferred
Extrapolated
CI
CrI
Date
Infected population (individuals)
Daily deaths (individuals)
Raw
Processed
Expected

Figure S1.68

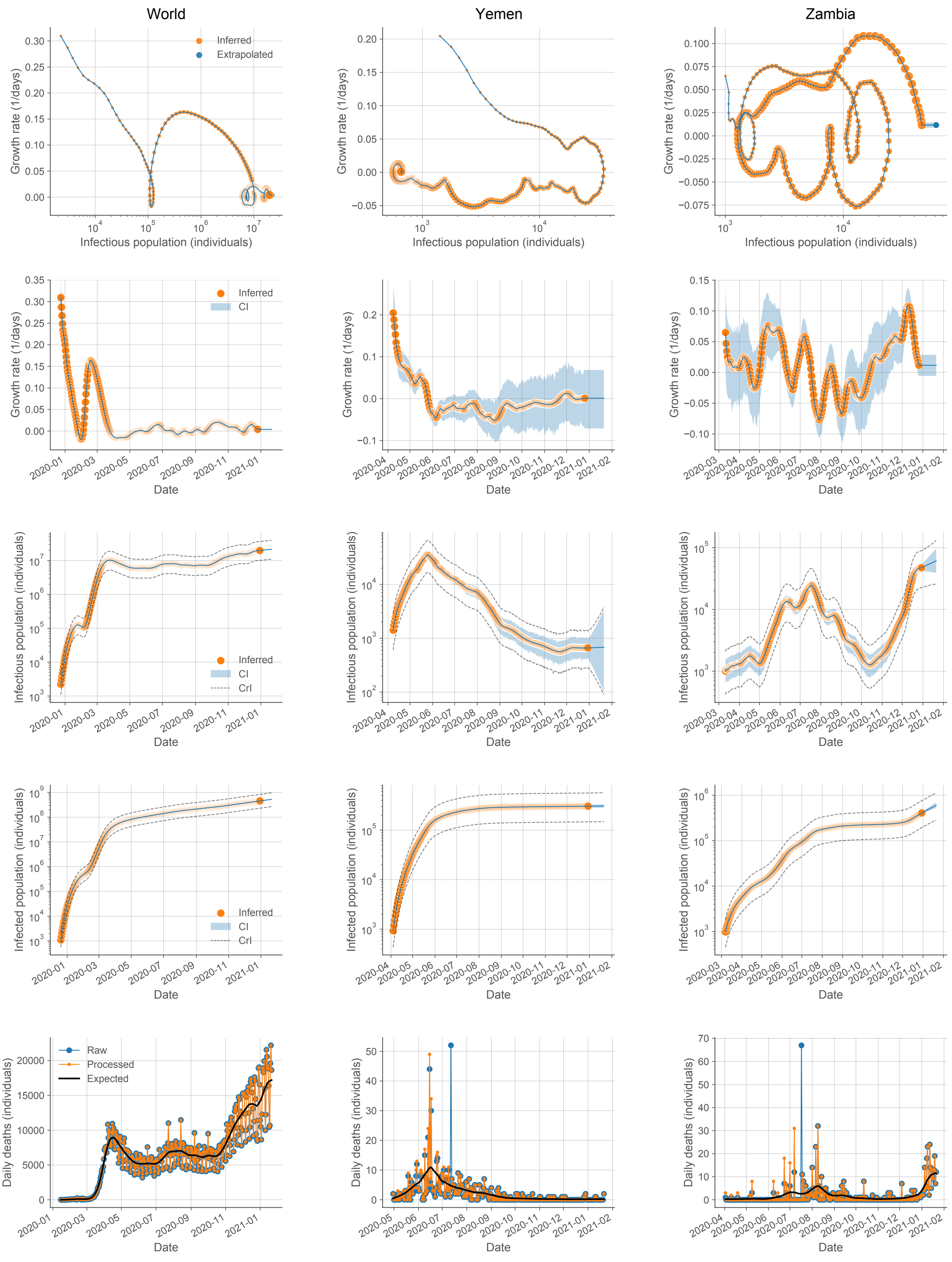
World
Yemen
Zambia
Growth rate (1/days)
Infectious population (individuals)
Inferred
Extrapolated
CI
CrI
Date
Infected population (individuals)
Daily deaths (individuals)
Raw
Processed
Expected

Figure S1.69

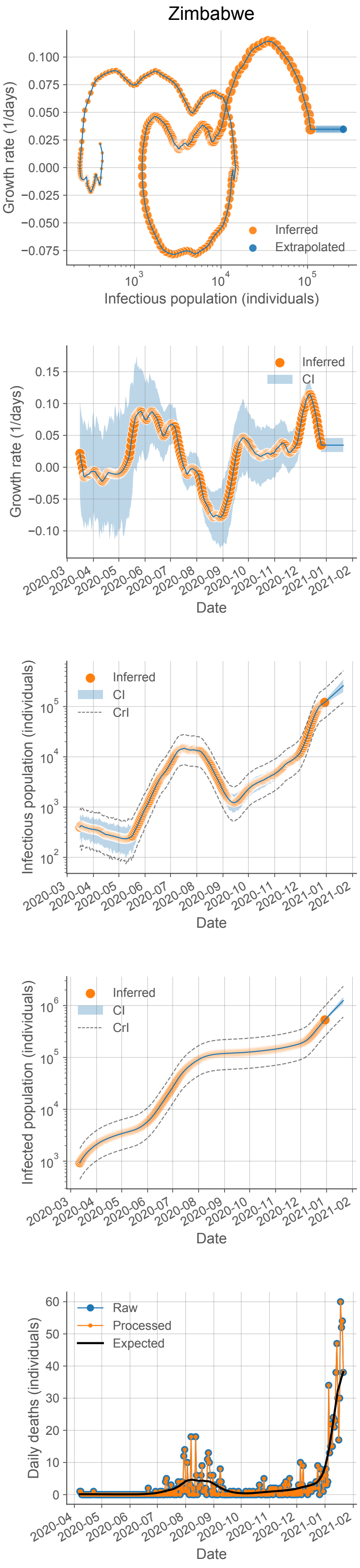
Zimbabwe
Growth rate (1/days)
Infectious population (individuals)
Inferred
Extrapolated
CI
CrI
Date
Infected population (individuals)
Daily deaths (individuals)
Raw
Processed
Expected

Figure S1.70
